# Socio-economic analysis of short-term trends of COVID-19: modeling and data analytics

**DOI:** 10.1186/s12889-022-13788-4

**Published:** 2022-08-29

**Authors:** Mostapha El Jai, Mehdi Zhar, Driss Ouazar, Iatimad Akhrif, Nourddin Saidou

**Affiliations:** 1 Euromed Center of Research, Euromed Polytechnic School, Euromed University of Fes, Fes, Morocco; 2 Ecole Nationale Supérieure d’Arts & Métiers, Moulay Ismail University, Meknes, Morocco; 3 IMS Team, SIME Lab, ENSIAS, Mohammed V University, Rabat, Morocco; 4 Mohamadia School of Engineers, Mohamed V University, Rabat, Morocco; 5 Euromed Center of Research, INSA-Euromed, Euromed University of Fes, Fes, Morocco

**Keywords:** Socio-economic analysis, COVID-19, SIR model, Short term propagation, Data analytics, Supervised machine learning

## Abstract

**Background:**

COVID-19 caused a worldwide outbreak leading the majority of human activities to a rough breakdown. Many stakeholders proposed multiple interventions to slow down the disease and number of papers were devoted to the understanding the pandemic, but to a less extend some were oriented socio-economic analysis. In this paper, a socio-economic analysis is proposed to investigate the early-age effect of socio-economic factors on COVID-19 spread.

**Methods:**

Fifty-two countries were selected for this study. A cascade algorithm was developed to extract the R0 number and the day J*; these latter should decrease as the pandemic flattens. Subsequently, R0 and J* were modeled according to socio-economic factors using multilinear stepwise-regression.

**Results:**

The findings demonstrated that low values of days before lockdown should flatten the pandemic by reducing J*. Hopefully, DBLD is only parameter to be tuned in the short-term; the other socio-economic parameters cannot easily be handled as they are annually updated. Furthermore, it was highlighted that the elderly is also a major influencing factor especially because it is involved in the interactions terms in R0 model. Simulations proved that the health care system could improve the pandemic damping for low elderly. In contrast, above a given elderly, the reproduction number R0 cannot be reduced even for developed countries (showing high HCI values), meaning that the disease’s severity cannot be smoothed regardless the performance of the corresponding health care system; non-pharmaceutical interventions are then expected to be more efficient than corrective measures.

**Discussion:**

The relationship between the socio-economic factors and the pandemic parameters R0 and J* exhibits complex relations compared to the models that are proposed in the literature. The quadratic regression model proposed here has discriminated the most influencing parameters within the following approximated order, DLBL, HCI, Elderly, Tav, CO2, and WC as first order, interaction, and second order terms.

**Conclusions:**

This modeling allowed the emergence of interaction terms that don’t appear in similar studies; this led to emphasize more complex relationship between the infection spread and the socio-economic factors. Future works will focus on enriching the datasets and the optimization of the controlled parameters to short-term slowdown of similar pandemics.

**Supplementary Information:**

The online version contains supplementary material available at 10.1186/s12889-022-13788-4.

## Background

Modeling pandemic propagation is one of the most complicated subjects that are studied as dynamic systems or stochastic problems. Several approaches have been developed in order to enhance the understanding of the pandemic kinetics through a population. Phenomenological models like the basic SIR model (Susceptible, Infected, Recovered) and the related upgraded versions try to simulate the way a pandemic evolves [1]. The SIR models are described by a system of Ordinary Differential Equations (ODEs) for which the initial conditions depend on the space and time considerations, according to the characteristics of each country [2–4].

Researchers developed number of approaches in order to estimate the different characteristics of the outbreaks evolution; Vizi et al. adopted pair-wise models with Markovian infection and arbitrary recovery processes that vary, so that the effect of recovery process choice is estimated [5]; while other introduced additional SIR compartments to quantify different aspects on the propagation mechanisms and disease transmission. For instance, Maier and Brockmann proposed a new symptomatic-quarantined infected population compartment, [6], while Nadim et al. incorporated additional compartments such as quarantined, asymptomatic, and isolated compartments to simulate and catch the short-term behavior of COVID-19 and to discuss the preventive strategies against it [7]. Other studies built up physical-inspired approaches like the recrystallization Ostwald Growth theory to study different containment scenarios; the containment strategies were proved to slow down the kinetics of the pandemic as well as the wall boundaries should do for kinetics of crystal’s growth [8]. Samely, Bouchnita and Jebrane used the physics of particles dynamics to study the dynamics of pair-wise contact models between individuals that belong to a closed population. The characteristics of the closed region and the population that were studied are included as main features of the simulation so that it was possible to quantify the effect of the demographic characteristics on the outbreak propagation in closed regions [9]. Other researchers were more interested by the mathematical structure of the SIR models; the existence of the solution of the problem and the different scenarios are built up by varying the input of the simulations. That is why, based on the SIR model, Katriel studied the seasonality of the pandemic and proved the existence of the return period of a given pandemic while R0 is higher than 1 [10].

Furthermore, other researchers tried to figure-out the eventual relationships that might exist between socio-economic characteristics of countries and the disease kinetics. Most of these researches handle systemic models in terms of time series modeling [11, 12], statistical analysis [13, 14], stochastic and dynamic analysis using epidemiological modeling [1–4]. For instance, Nader et al. [15] used non-parametric machine learning model to estimate the Non-Pharmaceutical Interventions (NPI) effects on COVID-19 propagation; based on the simulations, the authors summarized numerous conclusions related to short-term pandemic propagation in schools or according to business activities in 176 countries (that was expressed by means of GDP per capita). Symmetrically, Lee et al. [16] studied different scenarios of schools re-opening in Shanghai in terms of pandemic propagation regarding the age-structure and different contact patterns. Within the same scope, Arachchi and Managi [17] associated the death rate of COVID-19 to the social behavior for different countries; this statistical analysis included the social capital based on multidimensional analysis as community attachment, social trust, family bonds, and security. The study figured-out interesting observations of death increase according to population density and ageing, while it is the inverse as the number of hospital beds increases and lockdown policy is applied [17]. Similar results were produced by Kaufman et al. [18] proving that social distancing mandates the spread of the pandemic to decrease in USA, supporting the fact that NPI are as mostly importance even in case of vaccination.

In sum, it is remarkable that the approaches adopted in literature regarding COVID-19 spread description and modeling can be grouped into three main categories: stochastic processes, epidemiological models, physics inspired, and socio-economic approaches.

Recent works were more dedicated to socio-economic factors that should impact the disease propagation, but to a less extent they were focused on first order features, even for models involving logarithmic or exponential terms. In fact, the authors of this paper could not find publications that proposed higher order or interaction terms in their modeling to figure-out higher complexity analysis. Hence, a synthetic reading of these articles within the benchmark section of this paper will enlighten this point. After all, it is worth recalling some major challenging obstacles especially regarding data availability and completeness; several developing countries do not exhibit standardized statistics or there is, in general, lack of them, while for the developed countries, data is well-classified and available on several official web sites and publications; that led the authors of this paper to reduce of the selected countries from around 200 to 52 countries. In addition, another main obstacle is that rare are the articles that details the technics that are adapted and adopted for SIR parameters identification, and by extension R0, and J* estimation. Consequently, we have proposed the cascade algorithm as it is introduced above and detailed in the next sections.

In this paper, it is proposed to carry-out a macroscale socio-economic investigation by evaluating the reproduction rate R0 and the period (or day) J* according to a set of standardized socio-economic indicators. The day J* expresses the first important decrease day (shift day) of the infection accounted from the declaration of the first day of infection by the authorities. J* is proposed in this paper as a damping performance time indicator related to the short term government’s policies that were adopted against the outbreak. Hence, this parametric socio-economic approach was designed to emphasize the most significant socio-economic factors that should influence somehow the pandemic evolution. To illustrate the methodology, 52 countries were selected regarding data availability and completeness.

For each country, the ratio R0 was computed according to Eq. (3) [1] as presented in the SIR model section; J* was computed according to an inferential-based algorithm that is developed in the next sections. The initial conditions of the system (1.1–1.6) (or 2.1 - 2.6) were estimated by means of least square formulation and computed using a gradient free algorithm developed in this paper. Subsequently, *R*_0_ and J* were modeled according to the socio-economic indicators by means of multilinear stepwise regression (SW-MLR). A multicollinearity assessment was conducted so that a minimization of the Variance Inflation factors (VIF) of the predictor’s factors and models terms was achieved. The four designed algorithms were implemented as a whole-integrated cascade algorithm to reach the objectives of this research.

The present paper will be organized as follows: the next section is dedicated to the adopted methodology; the mathematical formulation of the problem to be solved is detailed in the third section; the fourth section presents the results of the proposed approach and the corresponding socio-economic discussion; after that, a benchmark study is carried out to compare the results of the present study with other references in terms of similarities and contrast; this allowed positioning our work regarding the existing literature; and the last section exhibits conclusive remarks and the perspectives of this work.

## Methods

The methodology adopted consists in:

- Data collect, cleaning, analysis, and primary data scatter visualization;
- Dimensionless data normalization;
- Resolution of the inverse problem by identifying the SIR optimal parameters (*β*^∗^, *μ*^∗^) and *J*^∗^ for each country by means of the cascade algorithm;
- Identification of the initial values of SIR system using a minimization Randomized Gradient Free Algorithm (RGFA);
- Stepwise regression of the SIR parameters processed simultaneously with the multicollinearity analysis of the socio-economic input parameters;
- Factorial analysis and the corresponding benchmark study;

The socio-economic data constitute the input matrix of the stepwise regression procedures; they were selected from different databases as presented Datasets collect (steps 1 and 5) section. The dimensionality reduction of the SW-MLR models was ensured by means of the multicollinearity assessment that aimed to minimize the Variance Inflation Factors (VIF) of data, of the model’s predictors and terms. Concerning the SW-MLR, reasonable level of determination coefficients *R*^2^ (> 70%) was considered the regressive models. In addition, according to the large scale differences of the variables, dimensionless min-max normalization was applied on both input and output raw data.

### Workflow of the study

**Fig. 1:**
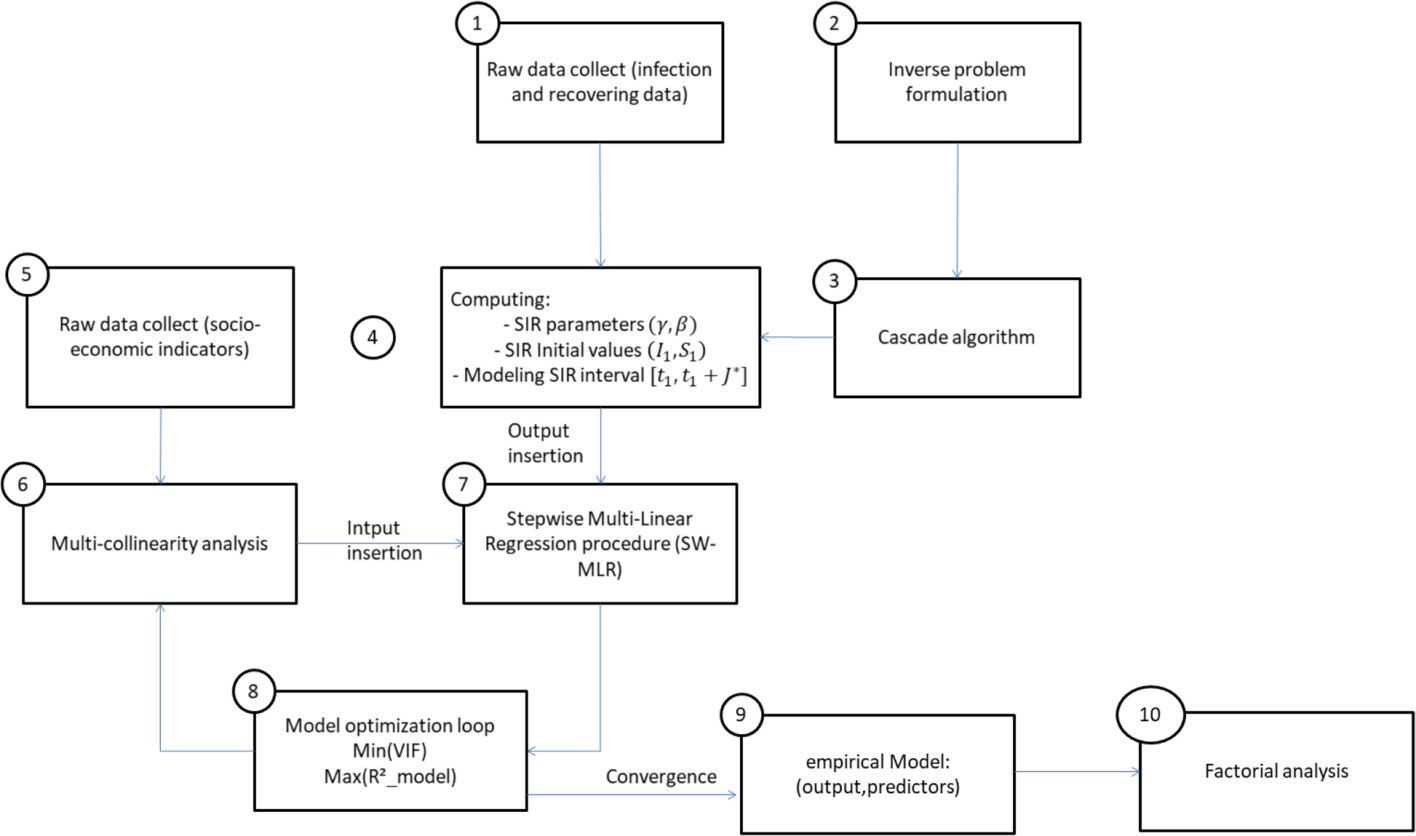
Architecture of the global analysis

Figure 1 presents the architecture of the global analysis and the flow chart that was drawn for this work.

The list below highlights the essential guidelines for understanding the global analysis of Fig. 1.

- **Step 1 and 5**: Data were loaded and collected from various databases as it is exhibited in Table 2;
- **Step 2**: The inverse problem formulation is developed in First phase: cascade algorithm results section in terms of the optimization problem (OP) to be solved;
- **Step 3**: According to the inverse problem developed in **step 2**, for each country, the cascade algorithm computes the SIR model parameters that are to be stored according to the **step 4**. The subroutines of the cascade algorithm are detailed in Second phase: Modeling the parameters according to socio-economic indices section and in the Additional file 1;
- **Steps 6 to 8:** coupled to multicollinearity analysis, SW-MLR algorithms were implemented as detailed in Summary of the findings section. The loop, constituted by the steps 6 to 8, expresses the multiobjective targets as the multicollinearity analysis aims to reduce the dimensionality by minimizing the VIF of the predictors; the maximization of the *R*^2^ ensures reliable models;
- **Step 9**: The convergence of the loop composed by steps 6–7-8 means that the VIF factor of each terms finally reaches the minimal value; the *R*^2^ of the regressive models also reaches the higher value. Hence, the models are returned and the significant predictors are maintained;

Finally, Results and discussion section displays the results of this work and discusses the main findings.

### Datasets collect (steps 1 and 5)

For this study, 52 countries were selected based on data availability and completeness; these latter are listed in the Table 1. The socio-economic features that are a-priori selected in this study are listed in the Table 2. They were selected so that the authors tried to group the most conventional socio-economic indicators that are in use in socio-economic analysis. COVID-19 statistics were collected from the references [19] (used also in [20]) and [21].

**Table 1:** List of countries selected for the analysis

Albania	Czech Republic	Japan	Russia
Algeria	Denmark	Kazakhstan	Serbia
Argentina	Egypt	Malta	Slovakia
Austria	Estonia	Morocco	South Africa
Belgium	Finland	Netherlands	Sri Lanka
Brazil	France	New Zealand	Sweden
Bulgaria	Georgia	Norway	Switzerland
Canada	Germany	Pakistan	Tunisia
Chile	Greece	Panama	Turkey
China	India	Philippines	Ukraine
Colombia	Indonesia	Poland	United Kingdom
Costa Rica	Israel	Portugal	Uruguay
Croatia	Italy	Romania	USA

**Table 2:** List of indices used in the study

Index	Index	Aspect	Data sources references^**a**^
GDP	Gross Domestic Product per capita	Economic	[22, 23]
HDI	Human Development Index	Economic, social	[24]
HCI	Health Care Index	Social	[25]
GSMI	Global Social Mobility Index	Economic, social	[26]
CO2	Carbone Dioxide emission	Economic	[27]
WC	Water Consumption	Economic	[28]
DBLD	Number of Days Before Lockdown	Non-pharmaceutical measure	**several websites**
Age	the elderly population more than 65 years old	Social, demographic	[19]
Tav	The temperature average of the countries that are considered	Observation index	From (https://en.wikipedia.org/wiki/List_of_countries_by_average_yearly_temperature) based on [29]

^a^All data sources were accessed on December, the 15rd 2020

### Mathematical approach and algorithms design

An inverse problem is a mathematical problem that deals with the determination of the parameters of Ordinary Differential Equations (ODEs) systems or Partial Differential Equations (PDEs) systems that should describe a set of functional data that are extracted from experiments or observations [30, 31]. Inverse problems are widely practiced in many industrial sectors (process, chemistry, biology, biotechnology, etc.) [31–33]. In 1982, James Ramsay developed a new concept in functional data analysis that is based on the minimization of a linear differential operator (LDO); Ramsay’s approach has been used in data classification and has been known, since this date, as Principal Data Analysis (PDA) [34]. The concept of PDA was introduced by Ramsay [34] instead of the Principal Component Analysis (PCA) which presents a general approach to the classical dimensionality analysis/reduction that could not be necessarily be modeled as smooth functions which is the case of functional data [35–38].

In this paper, a straightforward PDA approach for nonlinear system identification is developed. A specific formulation in the case of linear parameters ODEs system was drawn as well. The adaptation to SIR system was direct and the SIR parameters’ formulations were determined according to the procedure that is detailed in Additional file 1. This approach was applied for each country.

The following paragraphs details step-by-step the set of mathematical tools developed in this work.

#### SIR model

The system (1.1–1.6) and (2.1–2.6) display the (SIR) model that is adopted in this work as the basic form of the phenomenological models in epidemiology modeling [1, 2].

1.1 $$\frac{di(t)}{dt}=\beta\ i(t)s(t)-\mu\ i(t)$$

1.2 $$\frac{ds(t)}{dt}=-\beta\ i(t)s(t)$$

1.3 $$\frac{dr(t)}{dt}=\mu\ i(t)$$

1.4 $$i+s+r=1$$

1.5 $$\forall t\in \left[{t}_1,{t}_1+{J}^{\ast}\right]$$

1.6 $${i}_1+{s}_1+{r}_1=1\ at\ time\ {t}_1$$

2.1 $$\frac{dI(t)}{dt}=\frac{\beta }{N}\ I(t)S(t)-\mu\ I(t)$$

2.2 $$\frac{dS(t)}{dt}=-\frac{\beta }{N}\ I(t)S(t)$$

2.3 $$\frac{dR(t)}{dt}=\mu\ I(t)$$

2.4 $$N=I+S+R$$

2.5 $$\forall t\in \left[{t}_1,{t}_1+{J}^{\ast}\right]$$

2.6 $${I}_1+{S}_1+{R}_1=1\ at\ time\ {t}_1$$

s.t.

- i(t): is the normalized infection function reported to population unit, s(t): is the normalized susceptible function reported to population unit, r(t): is the normalized recovered function reported to population unit;
- I(t): is the estimated infection function, S(t): is the estimated susceptible function, R(t): is the estimated recovered function;
- i_1_, s_1_, r_1_: are the initial conditions respectively related to the functions i(t), s(t), and r(t);
- I_1_, S_1_, R_1_: are the initial conditions respectively related to the functions I(t), S(t), and R(t);
- 𝛽 and μ: are the stochastic parameters that must be computed to fit the model (1) (or (2)) to the observed data. In this study, the short-term and early-age analysis will be analyzed according to sole values of the couple (𝛽, μ) as it is detailed in the rest of the paper.

The reproduction number is to be computed by means of Eq. 3.

3 $${R}_0=\frac{\beta }{\mu }$$

#### Optimization problem definition (step 2)

The optimization problem (OP) is proposed in order to formulate the parameters identification problem and to derive the corresponding cascade algorithm of the OP problem.

4 $${\overrightarrow{\tilde x}}_1=\underset{\left(x_{1_1},\dots,x_{N_1}\right)}{\mathrm{argmin}}\left(\chi^2\right)$$

5.1 $${\chi}^2\left({\overrightarrow{x}}_1/\overrightarrow{\theta^{\ast }}\right)=\sum_{1\le i\le m}\sum_{1\le j\le N}{\epsilon}_{j_i}^2$$

5.2 $$\varepsilon_{j_i}={\tilde x}_{j_i}-x_{j_i}$$

5.3 $${\tilde x}_{j_i}={\tilde x}_{j}\left({t}_{i},\overrightarrow{\theta^{\ast }}\right)$$

6.1 $${\overrightarrow{\tilde x}}:= \arg \left( RK\left( ODES,{\overrightarrow{\tilde x}}_1,\left[{t}_1,{t}_1+{J}^{\ast}\right]\right)\right)$$

6.2 $$ODES:\overrightarrow{\dot{x}}=\overrightarrow{f}\left(\overrightarrow{x}(t),t,\overrightarrow{\theta^{\ast }}\right)$$

6.3 $${\overrightarrow{\tilde x}}={\left(\tilde{x}_1,\dots, \tilde{x}_N\right)}^t$$

6.4 $${\overrightarrow{x}}_1={\left({x}_{1_1},\dots, {x}_{N_1}\right)}^t$$

6.5 $$\left({x}_{1_1},\dots, {x}_{N_1}\right):= \left({x}_1\left({t}_1\right),\dots, {x}_N\left({t}_1\right)\right)$$

6.6 $${J}^{\ast }=\arg (PWP)$$

7.1 $$\overrightarrow{\theta^{\ast }}= argmi{n}_{\overrightarrow{\theta}}\left({\chi}_{PDA}^2\right)$$

7.2 $${\chi}_{PDA}^2\left(\overrightarrow{\theta}\right)=\sum_j\sum_i{Lx_j}_i^2$$

7.3 $${Lx_j}_i={x_j^{\prime}}_i-{f}_j\left({\overrightarrow{x}}_i,{t}_i,\overrightarrow{\theta}\right)$$

s.t.

- ${\overrightarrow{\tilde x}}_1\left(\tilde{x}_{1_1},\dots, \tilde{x}_{N_1}\right)$ is the optimal vector of the initial values of the solutions ${\overrightarrow{\tilde x}}\left(\tilde{x}_1,\dots, \tilde{x}_N\right)$ at the time t_1_;
- ${\overrightarrow{\tilde x}}$ is the solution of the Rang-Kutta (RK) algorithm of the ODEs system denoted by the expression (6.1);
- *ODES*: expression of the ODEs system to be solved (Eq. 6.2). The SIR model expressed by means of normalized variable was adopted as depicted by system (1.1–1.6);
- *J*^∗^ is the length of the interval of time of simulation [*t*_1_, *t*_1_ + *J*^∗^];
- *t*_1_ is the initial time of the interval of simulation [*t*_1_, *t*_1_ + *J*^∗^];
- $\overrightarrow{\theta}$ is the vector of parameters to be optimized according to PDA approach (system 7);
- $\overrightarrow{\theta^{\ast }}$ is the vector of optimal parameters;

Hence, the ultimate goal of OP resolution is to compute:

- the optimal SIR parameters $\overrightarrow{\theta^{\ast }}=\left({\mu}^{\ast },{\beta}^{\ast}\right)$ as detailed in Cascade algorithm: PDA approach and parameters identification section;
- the initial conditions ${\overrightarrow{\tilde x}}_1={\left(\tilde{x}_{1_1},\dots, \tilde{x}_{j_1},\dots, \tilde{x}_{N_1}\right)}^T$ of each functions $\tilde{x}_j$. Projected to the case of COVID-19, the functions $\tilde{x}_j$ are the Infection rate I(t) and the susceptible rate S(t). The, the initial conditions are denoted *i*_1_ and *s*_1_ respectively for infection and susceptible rates at the initial time *t*_1_. The RGFA algorithm of resolution is displayed in  Randomized gradient free algorithm for initial condition computing section;
- the parameter *J*^∗^ was computed using a point-wise procedure (PWP) that is developed in this work and that is detailed in Cascade algorithm: point-wise procedure (PWP) for J∗ determination section.

#### Cascade algorithm and the corresponding subroutines (step 3)

##### Cascade algorithm: algorithm structure

**Fig. 2:**
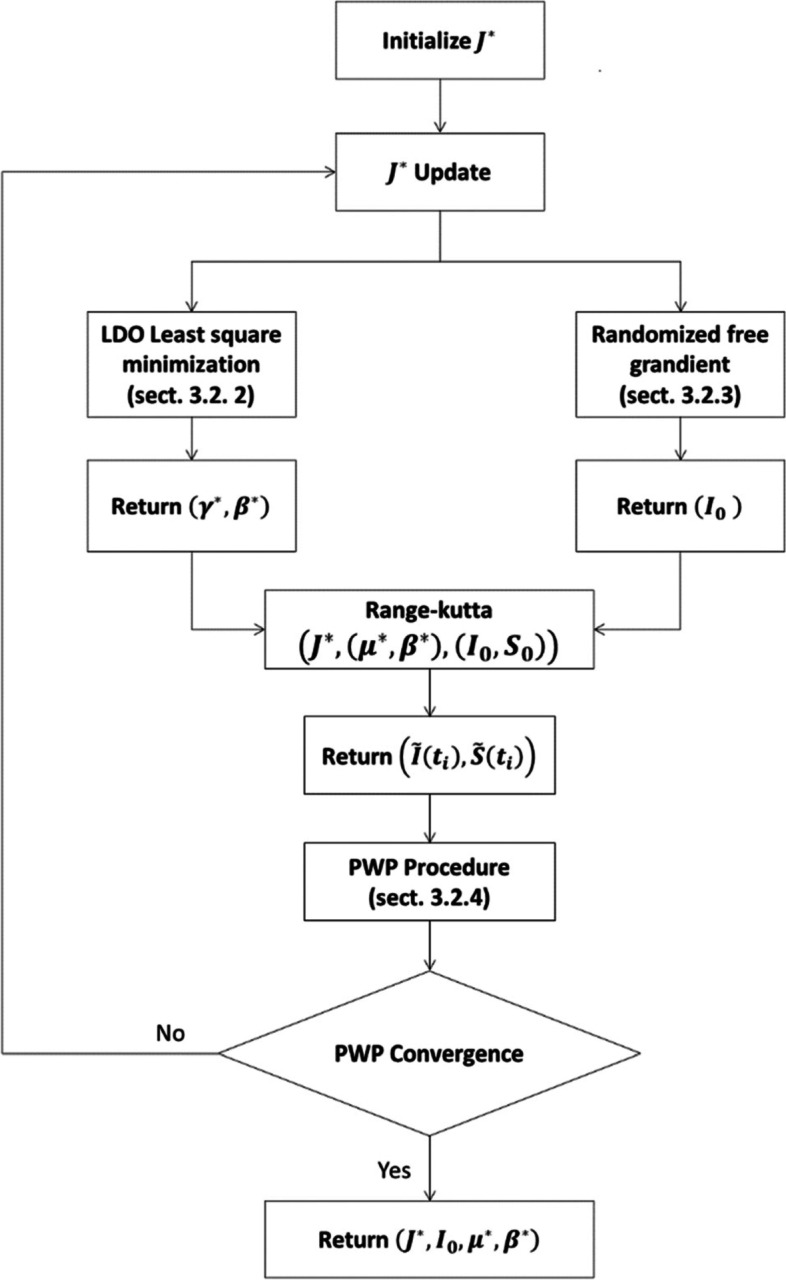
Cascade algorithm presentation

The cascade algorithm was designed to solve the OP problem and adapted to the SIR model. Figure 2 presents the main blocks of the cascade algorithm. Each step of this algorithm is detailed in the next sections (Stepwise regression and multicollinearity assessment, Modeling the shift day J*, Modeling the ratio R0 and Cascade algorithm: point-wise procedure (PWP) for J∗ determination).

##### Cascade algorithm: PDA approach and parameters identification

Let’s $\left({t}_i,{\overrightarrow{x}}_i\right)$ be a set of points of *ℝ*^*N* + 1^ that could be fitted by a set of first order ODEs as described by N equations of the system (8.1-...–8.N).

8.1 $${\dot{\tilde x}}_1(t)={f}_1\left({\overrightarrow{\tilde x}}(t),t,\overrightarrow{\theta}\right)$$

8.i $${\dot{\tilde x}}_i(t)={f}_i\left({\overrightarrow{\tilde x}}(t),t,\overrightarrow{\theta}\right)$$

8.N $${\dot{\tilde x}}_N(t)={f}_N\left({\overrightarrow{\tilde x}}(t),t,\overrightarrow{\theta}\right)$$

Where

- *t*_*i*_ is the times of observations as (1 ≤ *i* ≤ *m*);
- *t* is the continuous time parameter;
- ${\overrightarrow{x}}_i=\left({x}_{1_i},\dots, {x}_{N_i}\right)$ is the observed dataset at each time *t*_*i*_;
- ${\overrightarrow{\tilde x}}(t)={\left(\tilde{x}_1(t),\dots, \tilde{x}_N(t)\right)}^T$ is the vector of the fitting functions (estimated functions);
- ${f}_i\left({\overrightarrow{\tilde x}}(t),t,\overrightarrow{\theta}\right)$ represents the expression of the right term of the ODE (8.i);
- ${\dot{\tilde x}}_i(t)$ is the first order derivation of a function $\tilde{x}_i(t)$;
- $\overrightarrow{\theta}=\left({\theta}_1,\dots, {\theta}_p\right)$ is the vector of the estimated parameters of system (8.1–8.N);

It is to notice that the set of data observations ${\overrightarrow{x}}_i$ are to be normalized before proceeding to the computations. The classical least square method considers the error of estimation of the functions ${\overrightarrow{\tilde x}}={\left(\tilde{x}_1,\dots, \tilde{x}_N\right)}^T$ as described by the syst. 9 [39]:

9 $${\displaystyle \begin{array}{c}{\epsilon}_{1_i}=\tilde{x}_1\left({t}_i\right)-{x}_{1_i}\\ {}\vdots \\ {}{\epsilon}_{N_i}=\tilde{x}_N\left({t}_i\right)-{x}_{N_i}\end{array}}$$

As introduced above, it is proposed to study the inverse problem by means of the minimization of the sum of squares of the Linear Differential Operators (LDOs) that are denoted as *Lx* as exhibited in system 10 [36].

10 $${\displaystyle \begin{array}{c}{Lx}_{1_i}={\dot{x}}_{1_i}-{f}_1\left({\overrightarrow{x}}_i,{t}_i,\overrightarrow{\theta}\right)\\ {}\vdots \\ {}{Lx}_{N_i}={\dot{x}}_{N_i}-{f}_N\left({\overrightarrow{x}}_i,{t}_i,\overrightarrow{\theta}\right)\end{array}}$$

s.t.

- ${Lx}_{j_i}$ expresses the LDO operator related to the function *f*_*j*_ at the time *t*_*i*_;

The analytical development of the PDA Least Square Minimization (PDA-LSM) is detailed in Additional file 1. Hence, based on the PDA-LSM formulation, the optimal parameters (*β*^∗^, *μ*^∗^) of the SIR model (1.1–1.6) are to be computed according to Eqs. 11, 12.1, 12.2 and 12.3.

11 $$\left(\begin{array}{c}{\beta}^{\ast}\\ {}{\mu}^{\ast}\end{array}\right)={\left[A\right]}^{-1}\ \overrightarrow{b}$$

Where

12.1 $$\left[A\right]=\left(\begin{array}{cc}\sum_{1\le k\le m} \limits2\ {\left({i}_k{s}_k\right)}^2& \sum_{1\le k\le m}\limits-{i}_k^2{s}_k\\ {}\sum_{1\le k\le m}\limits-{i}_k^2{s}_k& \sum_{1\le k\le m}\limits{i}_k^2\end{array}\right)$$

12.2 $$\overrightarrow{b}=\left(\begin{array}{c}\sum_{1\le k\le m-1}\limits\left(\frac{i_{k+1}-{i}_k}{\tau }{i}_k{s}_k-\frac{s_{k+1}-{s}_k}{\tau }{i}_k{s}_k\right)\\ {}\sum_{1\le k\le m-1}\limits\left(-\frac{i_{k+1}-{i}_k}{\tau }{i}_k\right)\end{array}\right)$$

12.3 $$\tau =1$$

These parameters are valid within the time interval [*t*_1_, *t*_1_ + *J*^∗^]. As written in the summation symbol “*Σ*”, “*m*” denotes the size of the discretized interval of time [*t*_1_, *t*_1_ + *J*]. For each update of *J*^∗^, (*μ*^∗^, *β*^∗^) are also updated.

##### Randomized gradient free algorithm for initial condition computing

In general, Free gradient algorithms have been designed in order to solve optimization problems regardless the need of computing the objective function’s gradient [40]. A Randomized Gradient Free Algorithm (RGFA) is designed in this paper in order to compute the initial conditions of the SIR problem. In other words, RGFA aims to solve the system (4–5.3) of OP problem.

It is worth mentioning to recall that the initial conditions of the basic SIR system are coupled by the equation of the mass *N* of population conservation, in its normalize version (13).

13 $${i}_1+{s}_1+{r}_1=1$$

s.t.

- *i*_1_*: is the initial number of the infected population*
- *s*_1_*: is the initial number of the susceptible population*
- *r*_1_*: is the initial number of recovered people*

It is assumed here that, at the initial time *t*_1_, the number of recovered population is null since the immunity of the population could not be reached at the first instants of the pandemic. This statement allows directly setting *r*_1_ to 0 as a first assumption.

This allows correcting the initial conditions Eq. (13) that becomes (14):

14 $${i}_1+{s}_1=1$$

One can write the definition of the optimal initial condition ${i}_1^{\ast }$ as (Eq. 15):

15 $${i}_1^{\ast }=\underset{\left({i}_1\right)}{\mathrm{argmin}}\left({\chi}^2\right)$$

For each country, the real initial conditions $\left({I}_1^{\ast },{S}_1^{\ast}\right)$ are to be computed according to the system (16.1 and 16.2).

16.1 $${I}_1^{\ast }=N\ {i}_1^{\ast }$$

16.2 $${S}_1^{\ast }=N-{I}_1^{\ast }$$

Equation (15) corresponds to a constraints-free problem; for each country, the optimal value ${i}_1^{\ast }$ was computed by means of the RGFA algorithm as displayed below. This algorithm details the univariate unconstrained optimization problem in case of a unimodal function to be minimized (convex). The algorithm was then applied to the sum of the squared errors of the ODEs fitting. In other terms, the function f(x) that is considered in the expression (17) corresponds to the sum of the squared errors of estimation *χ*^2^ of the Infection rate function *I*; the argument *x* of Eq. (17) expresses the initial condition *i*_1_ as depicted in the expression (15).

17 $$(15)\iff {x}^{\ast }= argmin\left(f(x)\right)$$

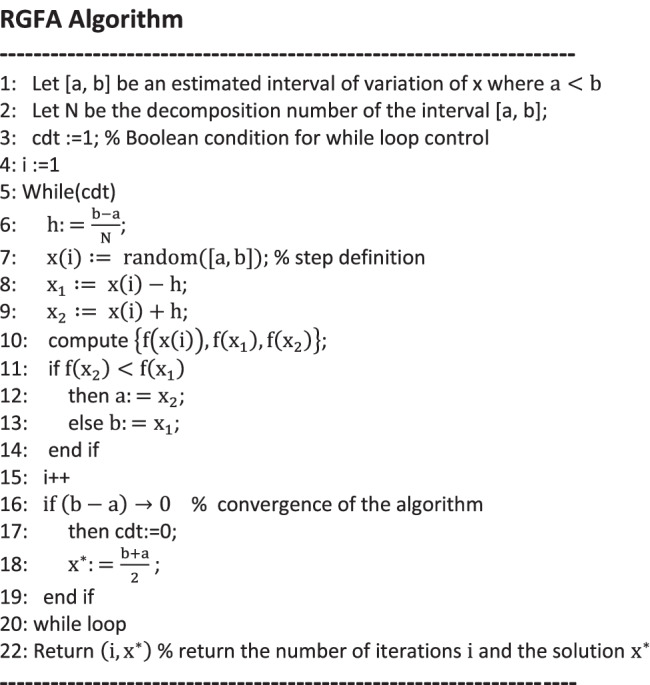


RGFA uses dynamic borders {{*a*}, {*b*}}. The current position x(i) is generated randomly within the current iterative interval [a,b] by means of uniform probability distribution. The detection of the decreasing directions are performed by adding a step ±*h* to the current position x(i); the functions values {f(x(i)), f(x(i))-h, f(x(i)) + h} are then computed. In consequence, the interval borders *a* and *b* are updated according to the evolution directions. The convergence of the algorithm is reached when the interval is reduced to a “supposed point of accumulation” denoted {*x*^∗^}. This solution can be seen as the convergence point of the series *a*_*n*_ and *b*_*n*_ that express dynamic borders of the pre-defined interval of the optimization problem. The algorithm returns the number of iterations “*i”* and the corresponding solution “*x*^∗^”.

##### Cascade algorithm: point-wise procedure (PWP) for *J*^∗^ determination

A PWP procedure was developed for a point-by-point insertion of an observed point (*t*_*i*_, *I*_*i*_) to a pre-existing fit using inferencial statistics. General speaking, the algorithm is based on a pre-determined set of points of which an interpolation is valid in terms of errors centering and model accuracy. After that an extrapolation of the pre-computed model is carried out for a new point (*t*_*i+1*_, *I*_*i+1*_). The error of extrapolation of this point is then analyzed according to the global errors vector behavior. If this new observation presents a reasonable level of fitting error, it is systematically added to the existing set of points to fit, and the model is recomputed. The adding or the rejection of a given point is managed by the procedure displayed in the next paragraphs.

**Fig. 3:**
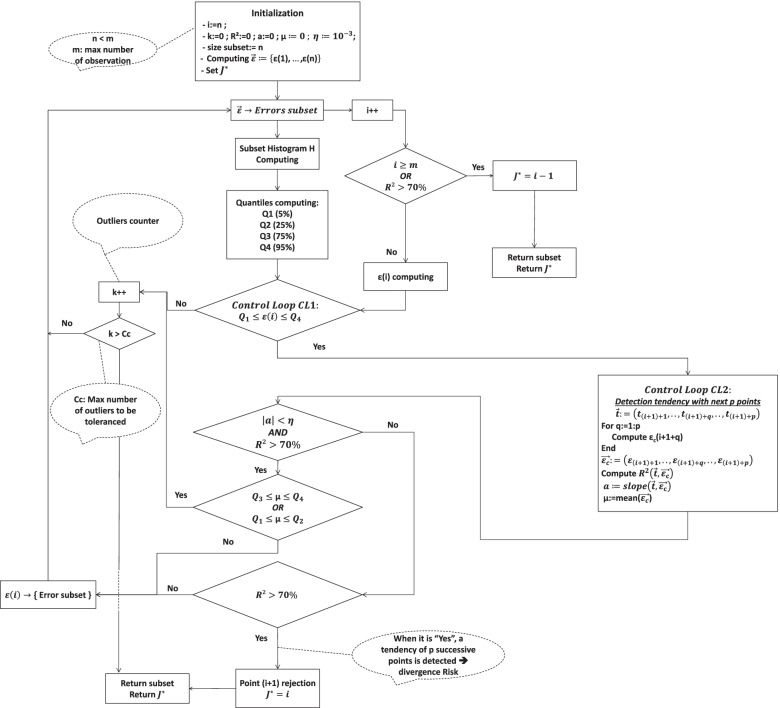
Point-Wise Procedure for optimal modeling interval detection in term of *J*^∗^ parameter

Thus, the PWP algorithm quantifies the likely trend of the error of estimation of I(t); indeed, this error must be centered on zero with a reasonable dispersion. The designed procedure was inspired by Statistical Process Control (SPC) in which the monitoring of a given property is carried out by means of control charts [41, 42]. Figure 3 depicts with details the PWP algorithm.

The PWP procedure starts by a minimal number of points (dataset of size N). The size N can be chosen according to each phenomenon and observer expertise. The assignment of the next point (i + 1) to the dataset is controlled by means of controlling the estimated error of extrapolation “ ${\varepsilon}_i=\overset{\sim }{y}\left({t}_i\right)-{y}_i$ ” as follows:

***For each iteration***

- Computing the histogram of the pre-defined dataset;
- Computing the quantiles {*Q*_1_(*α* = 5%), *Q*_2_(*α* = 25%), *Q*_3_(*α* = 75%), *Q*_4_(*α* = 95%)}. The quantiles are defined by the Eq. (18):

18 $$\alpha \%=P\left(e\le {Q}_i\right)$$

s.t.

“e” is the r.v. that is considered as the error of estimation at each time point t_i_;

- **1st control**: If the point (i + 1) is included in the range [*Q*_1_, *Q*_4_], the point (i + 1) is transferred to the 2nd control loop;
- **2nd control**: 7 next points are computed;
  - ° If a tendency of the (i + 1) with the 7 next points does not exist, it means that the estimated errors are fluctuating randomly around zero; else a tendency is caught which points-out the beginning of an important deviation from the zero line; the number 7 is fixed according to the SPC procedure [42]. This value can be changed to estimate the effect on the results of the PWP algorithm; this will be treated in future works;
  - ° The tendency behavior at a given point (i) is detected by computing *p* point after the current analyzed point (i). The slope and the coefficient of determination of the linear line $\left(\overrightarrow{t},\overrightarrow{\varepsilon}\right)$ are computed at these points; therefore, tests of tendency and deviation position to the zero line are to be performed by the algorithm. The tendency is estimated according to the slope “a” and the accuracy *η*:
    - ▪ If |*a*| < *η*: the tendency line is supposed horizontal and then a test of limits distance is leached in order to obtain errors that are not too distant from the zero;
    - ▪ If |*a*| > *η*: the tendency line is supposed evolving through a non-horizontal direction, increasing or decreasing, so that the error of estimation is actually shifting from the zero line; the centering of “e” on zero is no longer ensured;
    - ▪ Moreover, if the fluctuation of “e” is detected between the quantiles {*Q*_3_, *Q*_4_} or {*Q*_1_, *Q*_2_}, it means that the errors are positioned near the extreme error lines *Q*_1_ or *Q*_4_; this highlights a potential change of error average that could shift from the zero line;
- **Outliers detection and management:**
  - ° In case of outlier detection, in other term a point that does not fit the existing fitting curve, a counter “k” is incremented. If “k” exceeds a critical value “Cc”, the algorithm is forced to break and it returns the values of the errors subset and the optimal modeling time parameter *J*^∗^. The present algorithm proceeded by a setting “Cc = 20”. Other values of the “Cc” can be proposed. The effect of Cc choice will be discussed in future works;
  - ° Otherwise if no outlier is detected, neither out of range [*Q*_1_, *Q*_4_] nor caused by a tendency, the current computed point (i) is assigned to the subset of valid points; the algorithm loops;
  - ° If the index k exceeds the maximal number of outliers, the algorithm breaks and returns the parameter *J*^∗^ and then the errors subset $\overrightarrow{\varepsilon}=\left\{{\varepsilon}_1,\dots {\varepsilon}_N,\dots, {\varepsilon}_{J^{\ast }}\right\}$.

Figure 3 details the PWP algorithm that was implemented and applied on the error of estimation of the infection function.

#### Modeling using SW-MLR procedure (step 7)

Stepwise regression procedure is an automated procedure that is applied to find out the most influencing variables of a model in the case of important number of decision variables. The stepwise regression approach was firstly proposed by Efroymson [43]; it is an iterative procedure that works by adding and removing independent variables terms until reaching the targeted precision or reaching the minimal mean square error (MSE). The entrance or the exit of a model term is conditioned by the estimation of F-statistics, *p*-value or other valid statistics of the corresponding term. If the F-statistics is higher than a threshold value, the variable is maintained in the model, else it is eliminated [44].

Stepwise regression is applied for both linear and non-linear models [45]. For non-linear modeling, statisticians prefer to denote the model as generalized linear models in order to tackle wider range of data with different types of response variables [46]. The vector of variables or vector of predictors is denoted X and the model terms are denoted Z, where:

19 $${Z}_i=f\left(\overrightarrow{X}\right)$$

After stepwise algorithm convergence, the set of predictors are composed by the best input vectors X and the corresponding model terms *Z*.

Finally, the models could be written in two equivalent ways (20) or (21):

20 $$\overset{\sim }{Y}=f\left(\overrightarrow{X}\right)$$

Or

21 $$\overset{\sim }{Y}=f\left(\overrightarrow{Z}\right)$$

It is worth mentioning that the Step-wise regression algorithms belong to the set of Supervised Machine Learning procedures. For more details on the stepwise regression algorithm and the corresponding statistical tests, readers are referred to [44].

In addition, the modeling procedures should fit two main objectives that could be sensitively conflictual [43–45]:

- Minimizing the predictor’s number to avoid over-learning;
- Minimizing the bias of the model by selecting the necessary predictors variables, knowing that the elimination of a predictor could cause the loss of information;

##### Mathematical form of the adopted models (step 9)

In this study, general quadratic models were adopted for both R0 and J* as it is exhibited by the Eq. (22). General quadratic models were adopted due to the weakness of the first order and interactions models that were tested before proceeding to the current modeling.

22 $$y\left(\overrightarrow{x}\right)=\frac{1}{2}{\overrightarrow{x}}^TA\overrightarrow{x}+{\overrightarrow{b}}^T\overrightarrow{x}+\gamma$$

Where

- A is the symmetric matrix associated to the quadratic and interaction terms of the model y;
- $\overrightarrow{b}$ is the vector of constants associated to the first order terms of the function y;
- *γ* is the constant term of the function y;

After performing the hypothesis test for acceptance or rejection of the series of models, the “best” models can be selected according to the following equivalent indicators [44]:

- Coefficient of determination *R*^2^
- Adjusted coefficient of determination adjusted of *R*^2^,
- Mallows Cp statistics,

Moreover, structural multicollinearity was assessed and tested. This was ensured in this work by means of the Variance Inflation Factor (VIF) [47]. The modeling was applied for training countries and verified by the test dataset.

##### Multicollinearity assessment (step 6)

In the case of structural multicollinearity, prediction bias or overfitting should be eliminated. Hence in this paper, the stepwise procedure was constrained by VIF minimization that aims to reduce the inflation of bias due to an eventual structural and data multicollinearity. Since the VIF minimization causes the reduction of the dimensionality of the regressive models, the corresponding *R*^2^ were also verified and are tended to be maximized to produce models with reasonable accuracy and error. This procedure was programmed on matlab.

For each term “j” of a regressive model, the corresponding *VIF*_*j*_ is given by expression (23) [48]:

23 $$VI{F}_j=\frac{1}{1-{R}_j^2}$$

General speaking, the minimization of VIF and maximization of the precision of a model should be considered as a multi-objective optimization problem as well. Thus, one can transform this statement into mathematical form as it is expresses by expression (24).

24 $$mode{l}^{\ast }=\mathit{\arg}\left\{\begin{array}{c}\max (R^2)\ \\ {}{\min}_{1\le i\le q}\left( VI{F}_i\right)\end{array}\right.$$

s.t.

- “*q*” is the number of the model terms

Belslay [48] reported: VIF “measure is therefore of some use as an overall indication of collinearity. Its weaknesses, like those of the coefficient of determination, lie in its inability to distinguish among several coexisting near dependencies and in the lack of a meaningful boundary to distinguish between values of VIF that can be considered high and those that can be considered low” [48]; hence, we are proposing the interval [5, 10] as an acceptable variation range of the VIF factors [49, 50]. In addition, all normalized inputs were centered before proceeding to stepwise regression programming [48].

## Results and discussion

As it can be remarked from the methodology, the results of the proposed approach should consist in two main phases; the first is dedicated to the fitting of the SIR parameters, initial conditions computation, and the validity interval in terms of the day J*. the second phase corresponds to the modeling of these parameters according to the set of socio-economic parameters using SW-MLR procedure. A last sub-section of the discussion is dedicated to the benchmark study which led to position our study according to the existing references, and then to exhibit the similarities and differences with other specialized literature in terms of the more relevant socio-economic factors that are involved in COVID-19 spread.

### First phase: cascade algorithm results

**Fig. 4:**
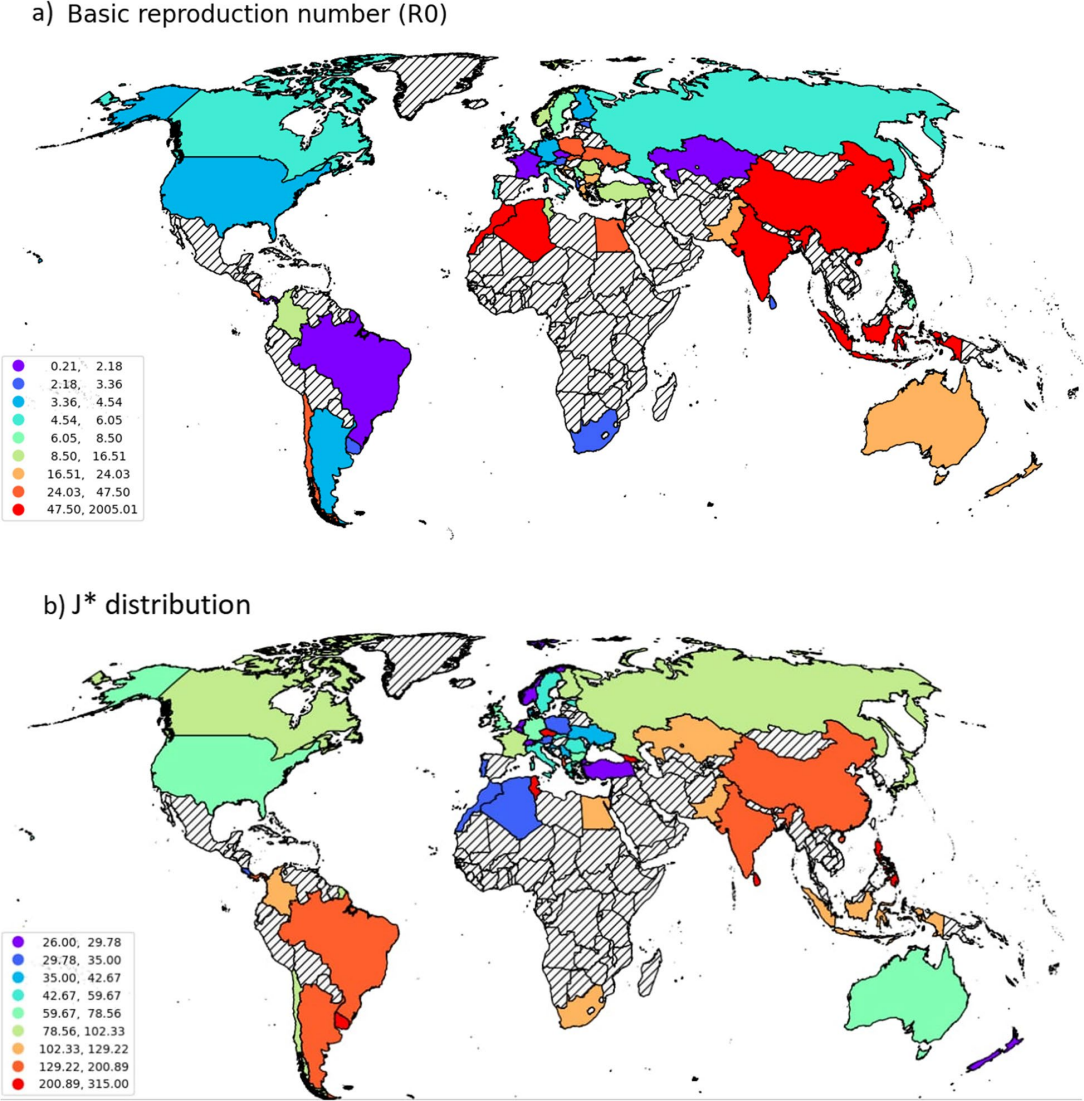
**a** Scatter plot of the basic reproduction number R0. **b** Scatter plot of the day J* (this vector map data is made with Natural Earth^(1)^ and programed with geopanda^(2)^; these two programs are public access). ^(1)^https://www.naturalearthdata.com/downloads/10m-cultural-vectors/ (Accessed on 15 January 2022). ^(2)^https://geopandas.org/en/stable/ (Accessed on 15 January 2022)

The application of the cascade algorithm results in a set of optimal parameters $\left({\beta}^{\ast },{\mu}^{\ast },{I}_0^{\ast },{J}^{\ast}\right)$ that are reported in the Table 3. The table is displayed according to a descendent sort of R0. Figure 4a and b display a colored scatter plot of R0 and J* per country.

**Table 3:** SIR parameters (*β*^∗^, *μ*^∗^), the initial value ${I}_0^{\ast }$, and the shift time point *J*^∗^

Country	β*	μ*	R0	***I***_0_*	RGFA iterat.^**a**^	J*^b^	PWP iterat.^**c**^
‘China’	0,042	2,09E-05	2009,569	4	10	100	75
‘Morocco’	0,0962	6,19E-04	155,412	5	11	33	8
‘Algeria’	0,0974	8,38E-04	116,229	5	13	35	10
‘Japan’	0,0721	8,14E-04	88,575	2	8	84	59
‘Indonesia’	0,0272	3,32E-04	81,928	63	17	111	86
‘India’	0,0466	6,81E-04	68,429	345	9	71	46
‘Costa Rica’	0,0548	1,32E-03	41,515	6	13	33	8
‘Poland’	0,0905	2,39E-03	37,866	26	12	33	8
‘Chile’	0,1404	3,75E-03	37,440	3	10	35	10
‘Ukraine’	0,0741	2,17E-03	34,147	27	11	42	17
‘Slovakia’	0,0477	1,86E-03	25,645	7	14	47	22
‘Egypt’	0,041	1,70E-03	24,118	25	10	105	80
‘New Zealand’	0,1378	5,75E-03	23,965	3	11	27	2
‘Pakistan’	0,0419	2,01E-03	20,846	60	9	113	88
‘Greece’	0,0455	2,22E-03	20,495	14	17	45	20
‘Australia’	0,0923	4,99E-03	18,492	3	11	67	8
‘Bulgaria’	0,0302	1,67E-03	18,084	8	12	67	42
‘Croatia’	0,0978	5,48E-03	17,847	3	6	36	11
‘Tunisia’	0,0617	4,46E-03	13,834	2	10	113	88
‘Colombia’	0,0392	3,44E-03	11,395	48	15	103	78
‘Turkey’	0,107	1,12E-02	9,554	270	15	27	2
‘Romania’	0,0486	5,19E-03	9,364	37	11	55	30
‘Denmark’	0,0699	8,08E-03	8,651	27	18	39	14
‘Norway’	0,0852	9,92E-03	8,589	31	11	29	4
‘Netherlands’	0,1171	1,51E-02	7,755	45	17	29	4
‘Malta’	0,0635	8,30E-03	7,651	3	16	36	11
‘Serbia’	0,0824	1,09E-02	7,560	22	17	38	13
‘Philippines’	0,027	3,78E-03	7,143	22	16	204	179
‘Sweden’	0,0698	1,03E-02	6,777	34	10	44	19
‘United Kingdom’	0,0996	1,67E-02	5,964	70	13	45	20
‘Israel’	0,1164	2,10E-02	5,543	15	16	35	10
‘Russia’	0,0671	1,23E-02	5,455	18	12	98	73
‘Portugal’	0,1183	2,32E-02	5,099	30	7	30	5
‘Italy’	0,106	2,27E-02	4,670	78	13	43	18
‘Canada’	0,0505	1,09E-02	4,633	127	12	59	34
‘Belgium’	0,1385	3,08E-02	4,497	33	16	29	4
‘Germany’	0,0849	1,96E-02	4,332	31	8	66	41
‘Argentina’	0,0363	8,62E-03	4,211	35	10	132	107
‘USA’	0,0974	2,78E-02	3,504	542	8	30	5
‘Finland’	0,0224	6,42E-03	3,489	26	11	79	54
‘Switzerland’	0,1375	4,06E-02	3,387	41	16	26	1
‘Austria’	0,0932	2,80E-02	3,329	40	7	35	10
‘South Africa’	0,0463	1,56E-02	2,968	38	11	127	102
‘Sri Lanka’	0,0157	5,64E-03	2,784	6	9	314	289
‘Albania’	0,0184	7,01E-03	2,625	7	8	178	153
‘Estonia’	0,0224	9,80E-03	2,286	11	9	59	34
‘Uruguay’	0,0378	1,72E-02	2,198	4	7	146	121
‘Kazakhstan’	0,0474	2,26E-02	2,097	11	13	116	91
‘France’	0,0681	4,43E-02	1,537	344	17	55	30
‘Georgia’	0,0623	4,16E-02	1,498	7	12	92	67
‘Brazil’	0,028	3,06E-02	0,915	1285	20	133	108
‘Panama’	0,022	3,86E-02	0,570	65	11	141	116
‘Czechia’	0,0421	2,01E-01	0,209	14	8	171	146

^a^Number of iteration of RGFA computed at the convergence of the algorithm

^b^The initial value of *J*^∗^ was set to 25 days

^c^Equal to the number of loops of PWP procedure

It is remarkable that the China shows an acute level of disease reproduction which is higher than 2000. A preliminary Weighted Sum of Squares (WSS)-elbow clustering according to R0 was performed on data of Table 3 showing that China constitutes one-class at each cluster number; it means that China should be considered as an outlier and must be eliminated from the rest of the regressive modeling.

**Fig. 5:**
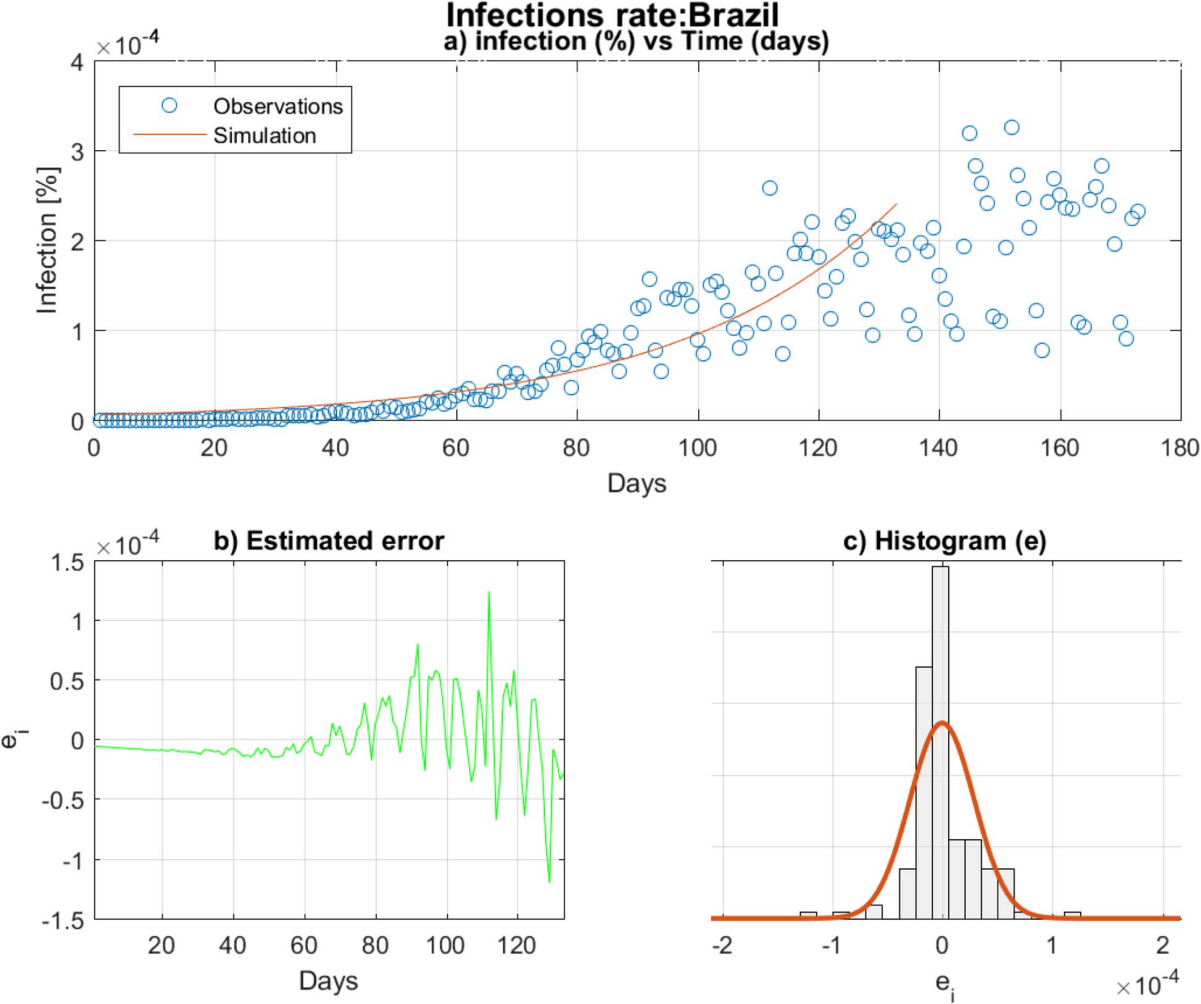
**a** Estimated infection function i(t) computed by cascade algorithm on the interval of time [1, 133]. **b** Plot of the errors of estimation. **c** Histogram of errors

To illustrate the results of the cascade algorithm, an example of estimated infection function i(t) of Brazil is exhibited in Fig. 5a, the shift day J* was estimated to be equal to 133 days. After that day, the observed infections drop so that the infection behavior changes for the first time from the infection declaration; the infection behavior is no longer exponential, this corresponds so to the early age of infection propagation in Brazil. As explained in the cascade algorithm of Fig. 2, the estimated parameters $\left({\beta}^{\ast },{\mu}^{\ast },{I}_0^{\ast}\right)$ are to be updated at each *J*^∗^ update. The convergence of the PWP procedure determines the final value of $\left(\ {\beta}^{\ast },{\mu}^{\ast },{I}_0^{\ast}\right)$.

The error of estimation is centered on zero as it is depicted in Fig. 5b and c; while the initial value *I*_0_ was numerically computed by the RGFA algorithm. Thus, for Brazil, *I*_0_ was estimated to be around 1285 infections after 20 iterations. Finally, the optimal SIR parameters (*β*^∗^, *μ*^∗^) are estimated to be equal to (0.025, 3.06 E-02). Additional file 2 groups the resulting plots of all countries that have been selected for this study.

### Second phase: modeling the parameters according to socio-economic indices

#### Stepwise regression and multicollinearity assessment

The second main phase of this article is to model the resulting parameters (*β*^∗^, *μ*^∗^), *R*_0_, and *J*^∗^ in term of the socio-economic parameters that are listed in Table 2; the set of input parameters are {GDP, HDI, HCI, GSMI, CO2, WC, DBLD, AGE, Tav}. To assess structural multicollinearity, VIF factors were computed in all steps of the backward stepwise regression procedure coupled to the quadratic model. Concerning the infection rate *β*^∗^, 137 models were generated by this procedure, while for the recovering rate *μ*^∗^, 37 models were generated. For the shift day J*, 346 models were generated. It is worth mentioning that all the corresponding *p*-values of these models are less than 5%, number of them are on the scale of 10^−6^. Nevertheless, although this procedure generated models with excellent *p*-values and *R*-squared, the high VIF levels, sometimes of the order of tens and mostly in the order of millions, lead to conclude that high multicollinearity bias are involved by different model terms in the produced models. This results in imprecise and insignificant models due to the elimination of the terms with high VIF values.

**Fig. 6:**
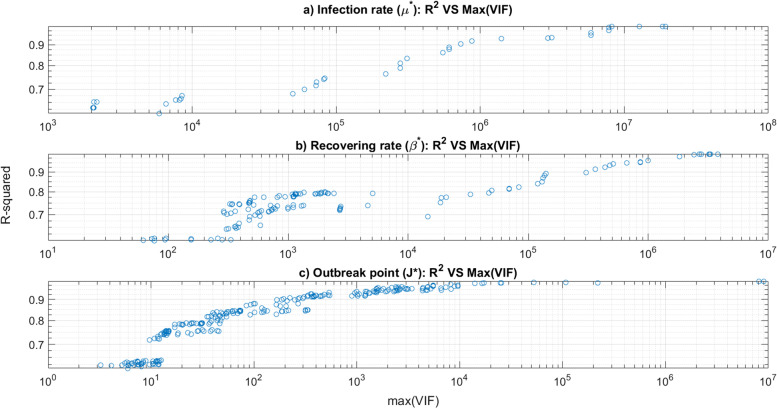
Stepwise indicators analysis: max(VIF) VS *R*-squared **a** for the infection rate *β*^∗^**b** for the recovering rate *μ*^∗^**c** for the shift point J*

Figure 6a to c depict 2D-plots of the maximal “VIF factors of the models” versus “the corresponding *R*-squared” in log-log space. One can conclude the increasing behavior of the *R*^2^ according to the VIF values. That is to say, the dimensionality reduction related to the decrease of structural multicollinearity by means of the minimization of VIF factors of the terms should enhance model’s *R*^2^. Furthermore, the max(VIF) factors of the terms of the modeled *μ*^∗^ and *β*^∗^ starts resp. around 2000 and 60 (resp. Figs. 5b and 6a).

In order to remedy multicollinearity, data were centered [40]; the interaction and the quadratic terms were then recomputed. Subsequently, the VIF factors were computed and multicollinearity was avoided by a stepwise elimination of the terms that generate high VIF values. Table 4 displays the resulting independent terms and the corresponding VIF factors. Hence, the stepwise regressions were carried out resulting in significant models of the reproduction number R0 and the shift day J* as it is detailed in the two next sections.

**Table 4:** Independent terms and the corresponding VIF

**Model’s term**	**CO2**	**DBLD**	**age**	**Tav**	**GDP_GDP**	**HDI_HDI**	**HCI_HCI**	**GSMI_GSMI**	**DBLD_DBLD**
**Term’s VIF**	4.852	2.514	3.720	5.779	3.973	4.712	4.341	7.265	5.320
**Model’s term**	**age_age**	**Tav_Tav**	**GDP_CO2**	**GDP_DBLD**	**GDP_Tav**	**HCI_GSMI**	**HCI_WC**	**HCI_DBLD**	**HCI_age**
**Term’s VIF**	5.320	7.300	3.883	4.504	9.593	5.519	3.665	4.583	5.565
**Model’s term**	**HCI_Tav**	**CO2_DBLD**	**CO2_Tav**	**WC_DBLD**	**DBLD_age**	**DBLD_Tav**	**age_Tav**		
**Term’s VIF**	6.072	6.300	4.649	9.452	9.297	6.112	6.808		

#### Modeling the shift day J*

**Fig. 7:**
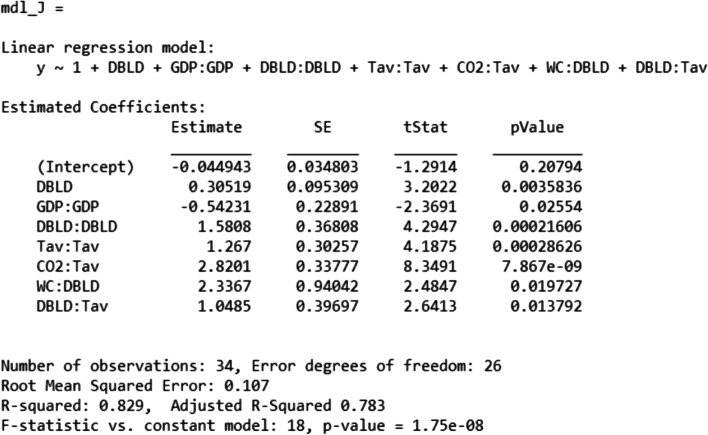
Best model of J*

The model of the shift day J* reached a reasonable accuracy in term of *p*-value, *R*^2^, and VIF factors as it is exhibited in Fig. 7; all the model terms and the model itself have significant *p*-values. Moreover, *R*^2^ is highly significant reaching more than 80% while the *R*^2^-adj reached more than 75%. It is noticeable that the intercept term is not significant since the corresponding *p*-value is higher than 5%. This is not quite important because this analysis especially deals with the variation of the infection parameters according to the socio-economic parameters. The model of Fig. 7 was computed using the training set composed by 36 countries.

**Fig. 8:**
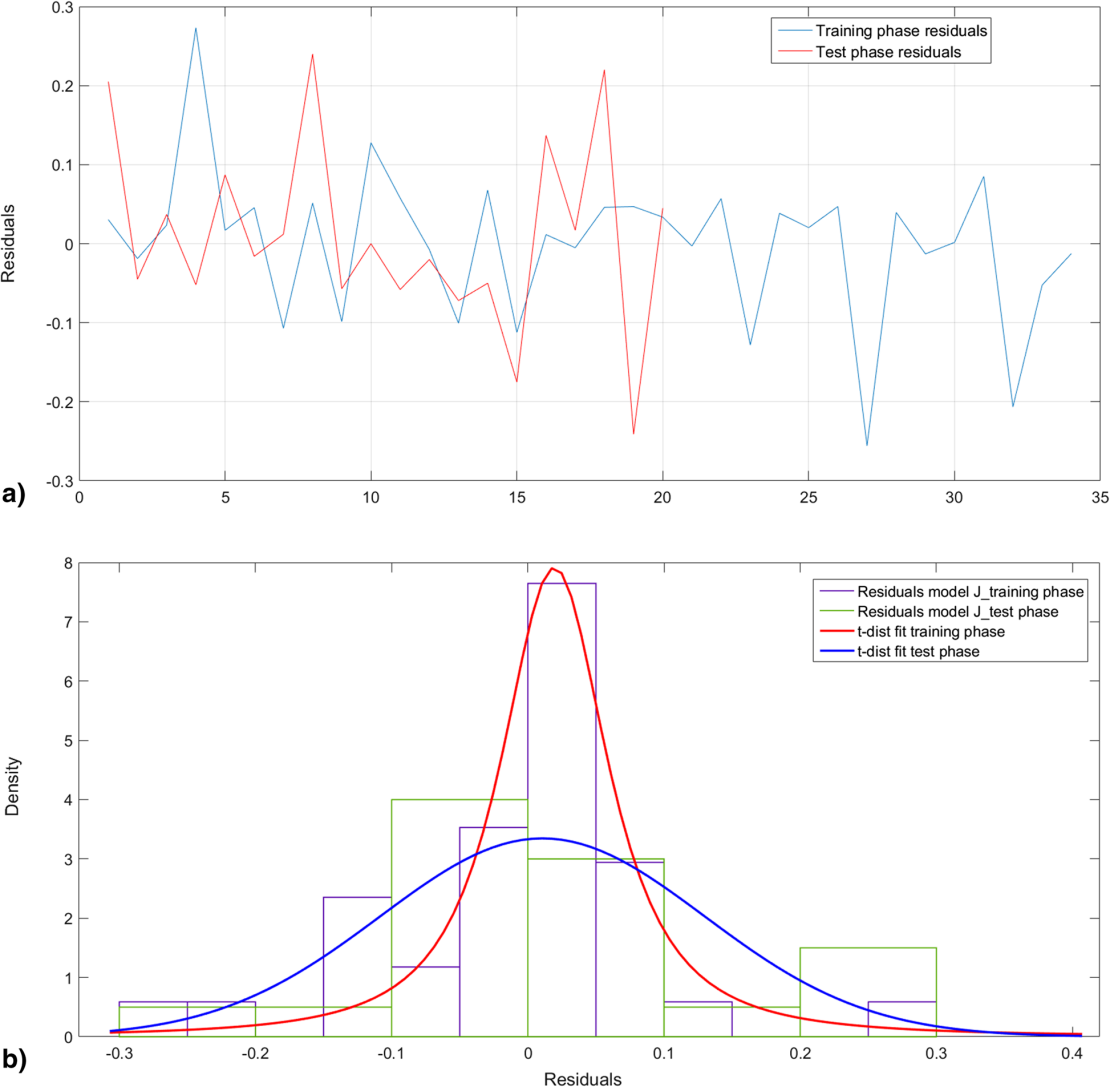
Error of estimation of J*. **a** Error of estimation **b** the corresponding histograms and t-distribution fitting curves

Figure 8 plots the histogram of the error of estimation and the corresponding t-distribution fitting. Figure 8a shows clearly that the test phase errors are quite included in the same range of variation of the error resulting from the training phase. Chi-squared test was carried out showing that training and testing population belong to the same population that is t-distributed centered on zero.

**Fig. 9:**
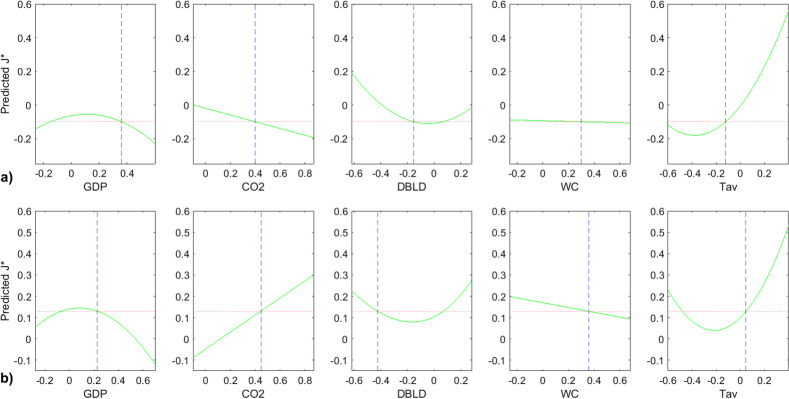
J* model slices of first order effects: “J* VS Predictors” **a** at a given position of Tav **b** at another position of Tav

Figure 9 displays the slices of the J* best model according to the predictors selected in the stepwise regression that are “DBLD, GDP, CO2, Tav, WC”. Based on this figure, Table 5 summarizes the behavior of J* according to each predictor.

**Table 5:** Behavior of J* according to the predictors

Predictor	Behavior of J*	Comment
GDP	Parabola	Concave
CO2	Linear	Decreasing slope
DBLD	Parabola	Convex
WC	Linear	Increasing or decreasing slope depending on Tav value
Tav	Parabola	Convex

**Fig. 10:**
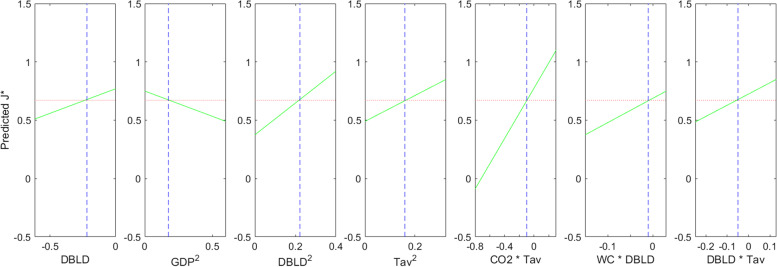
J* model slices: “J* VS model terms”

During the simulations, it was observed that the variations of J*, according to CO2 and WC, were very sensitive to the variation of Tav as it could be concluded from Fig. 9a and b comparison; according to Fig. 9a, the value of Tav forces the variation of J* according to CO2 to behave as a negative slope line, while it is totally the opposite for higher values of Tav as shown on the Fig. 9b. Subsequently, Fig. 10 is exhibited in order to highlight the second order interactions effects on J*. It should be mentioned that, according to Fig. 10, the shift day J* presents a stable behavior according to both first order and second order terms.

In general, it is clear that during a pandemic, the only parameter that can be handled in the short term is the DBLD that expresses the number of days before lockdown. Based on Fig. 10, the DBLD predictor affects the shift day J* as:

- a first order term increasing the J*;
- A second order term by increasing the J*
- with interactions with other predictors: water consumption parameter WC, and Tav parameter that corresponds to the “last 20 years” temperature average.

According to the Fig. 9, it is noticeable that J* behaves like a convex parabola according to the DBLD predictor. In addition, the minimum of this parabola depends on the current values of the other predictors. In contrast, J* behaves like a concave parabola according to GDP. Both peak’s position and value intensity of J*(GDP) parabola depend on the other parameters values.

It is important to recall that one of the major objectives of each country is to enforce the decreasing of the pandemic, which can somehow be expressed as the minimization of the shift day J*. Since, all the predictors are descriptive variables and are not easy to handle in the short term, the only way to reach this objective is to tune the DBLD in order to minimize the value of J*. For each country, this latter can be ensured by solving the Eq. (25). This point will be detailed and discussed in future works.

25 $$DBL{D}^{\ast }= argmin\left({{J}^{\ast }}\!\left/ \!{\left( GDP, CO2, WC, Tav\right)}\right.\right)$$

#### Modeling the ratio R0

**Fig. 11:**
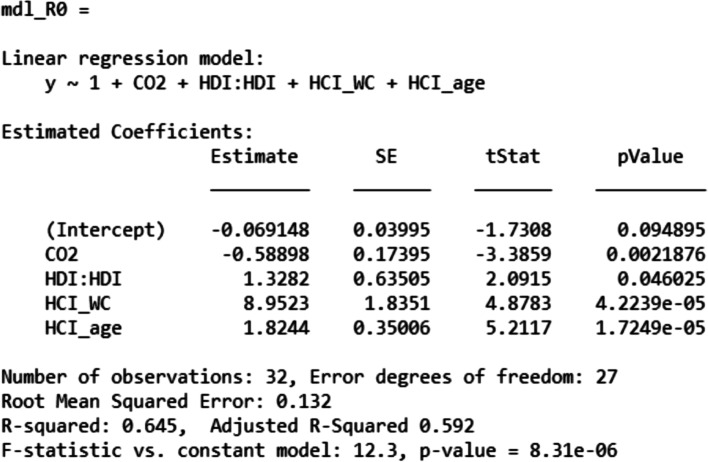
Best model of R0

The reproduction number R0 was modeled by the same procedure like J*; it was also computed using the same training set. Figure 11 details the model of R0 expressed by the corresponding set of socio-economic predictors. Based on the *p*-values, it was noticeable that the model and the predictors are significant except the intercept term (which is not of great importance for the factorial analysis). Moreover, *R*-squared is ranged between 60 and 70%, that is to say, the model can be used to have a preliminary insight of the behavior of R0 according to the socio-economic predictors.

**Fig. 12:**
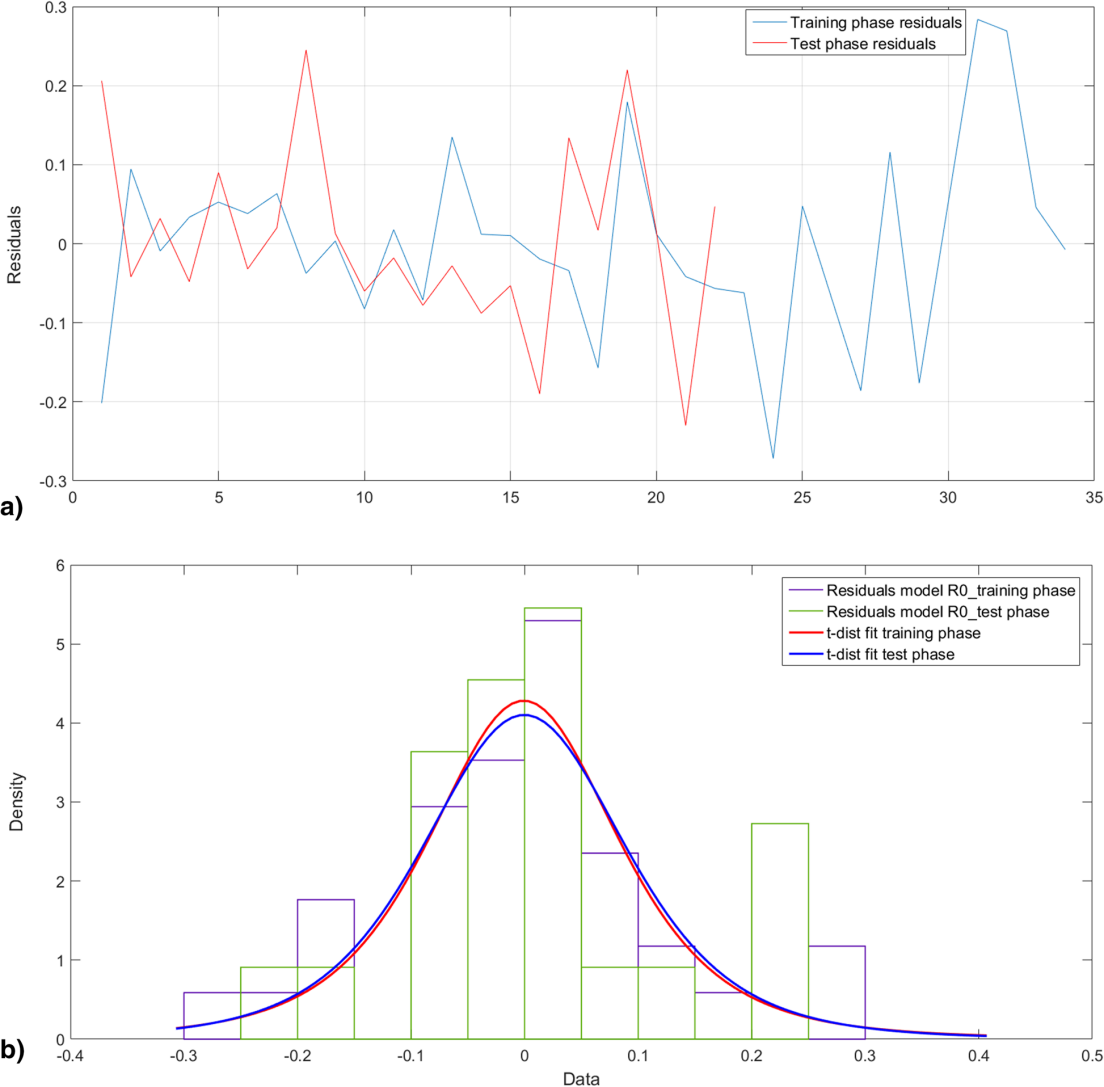
Error of estimation of R0. **a** Error of estimation **b** the corresponding histograms and t-distribution fitting curves

Test phase was assessed using the test set. Figure 12a displays the error of estimation of both training (blue) and test phases of the modeling. Figure 12b shows that the error of estimation is centered on zero for both training and test sets. Chi squared tests were carried and proved that both sets belong to the same.

**Fig. 13:**
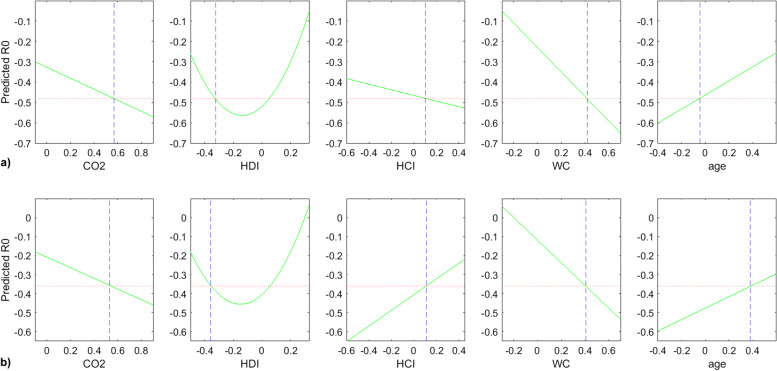
R0 model slices of first order effects: “R0 VS Predictors” **a** at a given position of age **b** at another position of age

Figure 13 displays the slices of the R0 best model according to the predictors “CO2, HDI, HCI, WC, age”. Based on this figure, Table 6 was produced to summarize the behavior of R0 according to each predictor. By comparing Fig. 13a and b, it is remarkable that the variation of R0 according to HCI (Health Care Index) is very sensitive to the age predictor variation. For instance, on the Fig. 13a, at a low elderly parameter (age), one can observe that R0 is decreasing according to HCI; that to say that smaller is the elderly, the smaller the R0 is, and thus, the number of infection is decreasing. In contrast, at higher values of elderly, as it is exhibited in the Fig. 13b, the health system is no longer able to ensure either the pandemic damping or the infection/death kinetics deceleration regardless the HCI level. The other parameters stably affect the ratio R0, in other words, the R0’s evolution according to {CO2, HDI, WC} shows a stable profile as depicted in Fig. 13a and b, and reported in Table 6.

**Table 6:** Behavior of R0 according to the predictors

Predictor	Behavior of R0	Comment
CO2	Linear	Decreasing slope
HDI	Parabola	Convex
HCI	Linear	Increasing or decreasing slope depending on age predictor
WC	Linear	Decreasing slope
age	Linear	Increasing slope

Other remarks are included in the Conclusion section in order to discuss more generally the R0 and J* behaviors according to socio-economic parameters involved in this study.

### Summary of the findings

Based on the simulations, number of conclusions can be depicted:

Related to shift day J*:

- the most important parameter that can be handled for J* reduction is the DBLD, and hopefully, this is the only parameter that can be tuned in the short term. It is worth mentioning that the reduction of J* leads to an early-age flattening of the infection curves;
- The other parameters that are involved in J* evolution are GDP and CO2:
  - ° J* behaves as a concave quadratic (parabola) of GDP which, at a given GDP level, J* is maximized, leading to a delay in pandemic damping. This behavior can be seen as an economic issue for which the human activity causes the increase of the disease propagation;
  - ° The variation $\frac{\partial {J}^{\ast }}{\partial \left(C{O}_2\right)}$ highly depends on the value of Tav: J*(CO2) is a decreasing line for low values of Tav; in contrast, J*(CO2) is an increasing line at higher values of Temperature average Tav;
- Related to J*(Tav) variation, it is noticeable that the increasing of the value of Tav implies the increase of J* independently of the other predictors values. Furthermore, as it was noticed about CO2 and WC predictor, Tav variation also affects the behavior of J* according to the other predictors; i.e. higher is the average temperature of countries, lower is the infection damping in term of higher values of J*;

Hence, in order to exploit the remarkable J* behavior, it would be interesting to simulate the likely values of J* for each county at the correspondent socio-economic parameters levels. This should allow determining the optimal values of the DBLD that might be adopted by each country in case of similar diseases, since DBLD is the only parameter that can be controlled by the authorities in the short term.

Concerning the reproductive number R0, it was shown that the parameters involved in its evolution are CO2, HDI, HCI, WC, and the elderly parameter. Furthermore, by varying the predictors positions in the simulation, it was highlighted that:

- the age parameter plays a crucial role in the R0 evolution according to HCI index; the slope $\frac{\partial (R0)}{\partial (HCI)}$ mainly depends on the elderly level. This variation is either negative or positive for respectively lower or higher values of age predictor;
- this behavior can inform the decision makers about the usefulness of a given health system (expressed by HCI index). In the present investigation, an elderly of 65 years old was implemented, but similar studies should produce very useful data by varying the elderly in order to detect deeper effect of this parameter on the infection propagation; this can be expressed by an “age structure” relationship;
- For high values of elderly, it was remarkable that the health care system could not control nor dampen the pandemic propagation since the variation $\frac{\partial (R0)}{\partial (HCI)}$ becomes positive for all values of socio-economic parameters; in other terms, corrective measures would not result in the expected effects. In consequence, the confinement, lockdown, and other preventive procedures would be more appreciable;
- In contrast, low elderly led to negative variation $\frac{\partial (R0)}{\partial (HCI)}$ which means that the health care system can participate to the decrease of R0 and then the deceleration of the disease propagation;

### Benchmark study

This benchmark study is dedicated to compare the findings of recent existing literature that treated almost similar analysis in terms of socio-economic factors’ implication in pandemic spread. This section also draws the similarities and the contrast between our findings and the literature. Table 7 summarizes the features (socio-economic indicators) that are used in the papers that were selected for this benchmark. It is possible that the papers displayed in Table 7 involved additional factors, but in order not to disturb the comparison with the current work, only common indicators were selected herein.

**Table 7:** The references used in the benchmark analysis and the associated socio-economic factors

Ref N°^a^	DBLD^b^	HCI	HDI	Age	Tav	GDP	CO2	WC
[1]	x		x					
[11]	x^d^	x^e^						
[15]	x							
[16]	x							
[18]	x^d^							
[51]	x							
[52]	x							
[53]	x							
[54]	x							
[55]	x							
[56]				x				
[57]	x			x				
[58]				x				
[59]				x				
[60]				x				
[61]			x					
[62]			x	x		x		
[63]			x					
[64]			x		x			
[65]		x			x	x		
[66]				x		x	x^c^	x^c^
[67]			x			x		
[68]					x	x		
[69]		x						
[70]		x						
[71]		x						
[72]	x	x						

^a^Reference N° in the present article

^b^Related to lockdown strategies

^c^Some articles involve the ecological footprint that can also be considered somehow in terms of CO2 emission and consumption in general, including water consumption parameter (WC)

^d^Analysis in terms of Non-Pharmaceutical Interventions (NPIs) and/or social distancing

^e^Analysis in terms of ICU-beds capacity

The references listed in the Table 7 analyzed the infection and deaths statistics cases according to different socio-economic indicators. Different approaches were proposed by the authors, including simple or composite indicators. In the majority of those papers, regressive models were considered for structural analysis. However and technically speaking, two main remarks emerged after analyzing these references:

- The majority of the papers adopted first order features
- Multicollinearity was not assessed nor studied

Nevertheless, the signs of the constants of the models that are related to the different variables (features) constitute the most interesting information that should demonstrate the tendency relationship between the infection state (dependent variable) and the descriptive variables. In the present article, the infection tendency was studied according to the reproduction number R0, and the day J* that gives an idea of the infection flattening in time.

The next paragraphs extend the benchmark discussion regarding the most important factors selected in the modeling phase of this research.

#### DBLD variable: the effect of lockdown policies

According to the analysis of the references [1, 51–55, 57, 69], it was remarkable that the application of lockdown policies enhanced the slow-down of the spread of the pandemic in space and time. For instance, based on real-time statistics in Libanon, Kharroubi et Saleh [52] demonstrated the success of the lockdown measures on the containment of the disease. Same results were attested by [51] who grouped time series data corresponding to 202 countries; their modeling emphasizes the negative and statistically significance of the lockdown contribution on the infection rates. The authors also proved that the infection curves flattening takes place 7 to 20 days from the rigorous lockdown implementation [51]. Similar results were found by Padhi et al. [54] in the case of India using SIRD (SIR+Death) modeling, and by [55] in case of USA. Other researchers simulated the results of lockdown application by the countries [1], and others, forecasted the COVID-19 propagation after school re-opening including the effect of age structure in Shangai by means of an adapted SEIR model [16]. Indeed, Lee et al. [16] proved that the re-opening of all children should maintain a baseline R0 of 3.3 and reducing the daily contact among children of 10–19 years old should decrease R0 to 33% from the baseline. Contrariwise, Born and coauthors [53] tried to understand the counterfactual case of Sweden, since Sweden did not apply the lockdown as for the other countries of EU. The outcome of this research showed a decrease of the infection and death curves by about 75 and 38% respectively. The NPIs should also reduce death by about 95%; SIR model was adopted for the simulations [53]. The other studies, reported in the Table 7, which involve lockdown in their analysis exhibit similar conclusions in terms of infection and death reduction after or while applying lockdown measures, based on statistics or simulations [1, 18, 57, 69].

Based on the above, it is noticeable that this research can provide similar findings, especially if we consult the curves of Fig. 10 that are related to DBLD parameter as a second order parameter “DBLD*DBLD” or as interactions terms “WC*DBLD” and “DBLD*Tav”. But in fact, the feature of our study is that the damping time J*(DBLD) behave like a convex parabola so that the analysis could not be independent of the other socio-economic factors, and it will depend on each country. We conclude here that, indeed, the reduction of the DBLD, which is the number of days before lockdown, should accelerate the infection flattening by reducing the time J*; but according to our modeling (Fig. 9), this is true above a given DBLD value, that depends on the other socio-economic factors. Under this critical value (the minimum of the parabola), the effect is inversed and the reduction of the early application of the lockdown will have no effect on the pandemic decrease.

#### HCI and age variables: the coupled effect of health care system and elderly

According to the literature, the health care system was considered by means of different indicators such as ICU level [11], Prevention and Control (P&C) capacity [71], the number of hospitals and the 4 T’s (tracing, tracking, testing and treating) [69]. In this work, and as previously introduced, HCI index was adopted as a public health policy indicator.

In the existing literature, it was notified that the enhancement of the previous heath policy indices (ICU, P&C, Hospitals number, and the 4 T’s) implies the reduction of the pandemic infection and death count. Even in the case of multidimensional analysis, the health policy was not speculated nor discussed from a wider perspective that could include the other socio-economic factors [62–68]. The age structure was also identified to have significant effect on the infection evolution, while elderly is assumed to be highly correlated to death cases; an exponential relation was denoted [57, 59]. Thus, for older adults, social distancing remains the appreciable and the well-encouraged strategy for risk prevention [58]. However, [56] showed how delicate is to assess the age-specific number of COVID-19 death associated with regards to seroprevalence statistics. In addition, contact patterns are then discussed, by the authors leading to a systematic explanation of the excess of death especially in nursing homes. This shows how age-structure can be robustly exploited to reconstitute the level of transmission [56].

Considering the above, it is remarkable that the previous references did not include interactions between the elderly (or the age-structure) and the health care system at all. Consequently, the proposed model of R0 herein proves the need of a higher order multidimensional insight (order higher than “1”) in terms of socio-economic predictors; this statement is justified in "Modeling the ratio R0" section. Hence, the health care system effectiveness cannot be assessed nor quantified without including the interactions with other parameters such as population elderly. Indeed, in the present work, it was proved and concluded that the infection spread, expressed by the R0 ratio, tightly depends on the interaction of HCI and the elderly parameter so that it is neither obvious nor logical to interpret the level of the health care system efficiency of a country independently on the age structure of the corresponding population. Hence, deeper analysis should be performed to draw a reliable picture of the correlation between the public health system and the infection state of a country. In consequence, the authors assume that the models that do not integrate interactions or higher order terms should present modeling bias or missing links in infection interpretation according to public health effectiveness even if they could show significant fitting parameters.

#### HDI, GDP, and Tav variables: a multidimensional analysis

Liu et al. (2021) presented one of the earlier papers in the literature that exhibited the unexpected positive correlation between HDI and the risk of infection and deaths of COVID-19. The infection rate and fatality rate of seven regions in Italy was modeled by means of HDI index, as a composite factor, but also according to the sub-components of the HDI such as the Average Annual Gross Salary. Liu et al. models statically proved the positive correlation between both infection and fatality rates with the HDI [61]. Identical conclusions were notified by Troumbis (2021) [66], and Thazhathedath Hariharan et al. [64]; in order to well interpret these findings, a particular attention was given to the high level of life expectancy of populations in the richest countries that have high HDI which can cause the increase of more death according the corresponding high elderly [63, 64]. Moreover, Thazhathedath Hariharan et al. [64] tried to find out an eventual multidimensional explanation of the infection by coupling socio-economic factors to environmental ones such as temperature, temperature anomaly, and humidity. The results of the corresponding simulations showed that the low temperature could allow the proliferation of the viruses but also temperature may cause host shifts (denoted as temperature anomalies) for viruses and increases the susceptibility of more susceptible species. Nevertheless, after including HDI in the models, the environmental factors lost their effects and temperature becomes meaningless compared to HDI and could not describe COVID-19 transmission [64]. In another hand, Ahmed et al. [65] included GDP and life expectancy among numerous other environmental factors (temperature, humidity…). The study concluded that the infection decreases with GDP but could not propose reliable remarks regarding temperature-infection relationship [65]. Muraniya and Varga [66] exhibited same conclusions on the GDP-infection infection in the case of rich countries; In contrast, for low income countries the infection spread is correlated to population density and health care conditions [66]. A biological explanation of the GDP-infection relation in rich countries, this relation is likely associated to the unbalanced ecological milieu and to the perturbation of the natural immunity (in terms of micro-organisms) that could be caused by the industrial stress and by pollution emission in such developed countries [66]. In the same way, Varotsos et al. [67] associated the death increase to HDI and the infection evolution to the GDP per capita. Anam et Shor [68] statistically found that COVID-19 infection decreases with temperature but increases according to GDP as proposed by the previous references.

According to the previous reading, the main remark is that the models already used in the literature did not present neither interaction terms nor high order parameters in terms of temperature, HDI, and GDP dependency; the authors based their analysis on one order parameters, while neither discussion nor analysis of multicollinearity verification or assessment is introduced apart Ahmed and coauthors [65] who reported that multicollinearity of the parameters vectors of his study will not bring perturbation to the interpretation of his models. This latter remark is fundamental in this comparison paragraph. For instance, Thazhathedath Hariharan [64] found that the effect of temperature could be negligible if the HDI parameter is introduced in the analysis. Furthermore, GDP parameter was found in the literature to be a increaser factor of infection in the case of richest countries, but for the other countries, its effect vanishes in presence of demographic factors [66]. According to our findings, reported in Fig. 9, the simulations proved that the profile of the infection-slow down (in terms of J* day) according to GDP parameter is associated to the temperature, and the GDP-infection relation is not as linear as it appears; as mentioned in Summary of the findings section, J*(GDP) is a concave curve, that depends on the other socio-economic factors. Temperature also affects heavily the profile of J*(CO2) where it is either increasing or decreasing according to temperature level. Hence, the key-point of modeling proposed in this paper is that the order of modeling should be higher than those proposed in the literature and the relationships between the infection and death rates or cases must be analyzed deeper in a multidimensional perspective.

## Conclusion

In this paper, it is proposed to draw up a macro-scale approach for understanding the pandemic propagation of COVID-19 according to socio-economic indicators. For the disease description, two main indicators were adopted; the critical shift day J*, that was proposed for the first time in this work which characterizes the first important decrease of the disease, and the reproduction number R0 that summarizes the macroscale infection time-kinetics. The study focused then on the early age of the pandemic. The methodology adopted was presented in general, and the case of SARS-COV-2 pandemic was analyzed by means of the basic SIR model. Fifty-two countries were selected according to data availability and completeness. Then, R0 and J* constituted the dependent variables to be modeled according to the socio-economic factors. Concerning the stepwise regression procedure, 2/3 of the countries were selected for the training phase and the last 1/3 served for the test phase.

The first phase of this research was achieved by means of the cascade algorithm that is composed by four sub-algorithms that were designed and implemented for each selected country. First, *β*^∗^ and *μ*^∗^ were computed by adapting PDA analysis to linear parameters problem. Consequently, the reproduction number R_0_ was estimated according to *β*^∗^ and *μ*^∗^. Injecting these results in the RGFA algorithm, the optimal initial input ${I}_0^{\ast }$ was determined, and the critical shift day J* was selected using the PWP algorithm that was developed in this paper. In the second phase of modeling, a series of SW-MLR were launched to model J* and R_0_. The candidate independent variables selected initially are the socio-economic parameters. Furthermore, data and structural multicollinearity were taken into consideration, treated, and eliminated within the stepwise regression procedures leading to reliable and accurate general quadratic models.

A detailed comparative study was conducted by means of a benchmark which focused on the multidimensionality of COVID-19 spread in association to the adopted socio-economic vision. This comparative study allowed pointing out the main novelties brought by our research in term of interactions and higher order models terms instead of first order parameters; in fact, this should reinforce the understanding of the pandemic spread in a wider window of the public health and to avoid neglecting the likely weak relationships between the infection statistics and some socio-economic or environmental factors. Indeed, in a first order analysis, some factors appear to be not significant, but by implementing higher order or interaction terms, this insignificance relation seems to be not negligible as the infection profile varies significantly after increasing the factors modeling order.

Future works will focus on the effect of the elderly-based age analysis on the outbreak’s propagation and its dependence to other socio-economic factors; other works will be dedicated to the computing of the optimal DBLD parameters for each country and comparative scenarios are to be developed in the case of optimal DBLD factor. In addition, the authors are working on an extension of the SIR model to a new Opened-SIR (O-SIR) model that will be coupled to socio-economic factors for a deeper understanding of the pandemic spread in a meso-scale perspective. Further works will focus on to the mathematical analysis of the algorithms that were proposed in this paper in term if complexity formulation, computation, convergence analysis, and applicability to other case of study related to epidemiological modeling and industrial applications.

Finally, the authors invite the readers to share their comments and critics in order to widen the perspectives of this analysis.

## Supplementary Information

**Additional file 1.** SIR parameters identification by Least square minimization and PDA approach [73, 74].

**Additional file 2.** Plot of the observed and the simulated infection rates.

## Data Availability

Data were gotten from online resources as it is exhibited in Table 2.

## Abbreviations

COVID-19: Coronavirus disease 2019
GDP: Gross Domestic Product per Capita
HDI: Human Development Index
HCI: Health Care Index
GSMI: Global Social Mobility Index
CO2: CO2 emission in millions of tons
WC: Water Consumption in millions of m^3^
DBLD: Number of Days Before LockDown
Age: Elderly of population (that are more than 65 Years old)
Tav: Average temperature in °C (computed for the last 20 years)
r.v.: Random variable
MSE: Mean Square Error
RMSE: Root Mean Square Error
SIR: Susceptible-infectious-recovered model
SIRD: Susceptible-infectious-recovered-death
P&C: Prevention and Control
