# Supplementary material for: Socio-economic analysis of short-term trends of COVID-19: modeling and data analytics
Source: BMC Public Health. 2022 Aug 29;22:1633. doi: 10.1186/s12889-022-13788-4 (PMC9421639; doi:10.1186/s12889-022-13788-4)
Supplement: Supplementary file 1 — Additional file 1. SIR parameters identification by Least square minimization and PDA approach [73, 74]. [file 12889_2022_13788_MOESM1_ESM.docx]

**Additional file 1: Least square minimization with PDA approach, adaptation to linear parameters and SIR Model identification**

1. **Least square expression**

Given the Linear Differential Equation $L{x_{j}}_{i}$ defined by the system (A.1), that is:

- related to each equation (A.1.j)
- at each time step $t_{i}$

$$\left( A.1 \right)\left\{ \begin{aligned} {Lx}_{1_{i}}=\dot{x}_{1_{i}}-f_{1}\left( \vec{x}_{i},t_{i},\vec{\theta} \right) \left( A.1.1 \right) \\ \vdots\\ {Lx}_{j_{i}}=\dot{x}_{j_{i}}-f_{j}\left( \vec{x}_{i},t_{i},\vec{\theta} \right) \left( A.1.j \right) \\ \vdots\\ {Lx}_{N_{i}}=\dot{x}_{N_{i}}-f_{N}\left( \vec{x}_{i},t_{i},\vec{\theta} \right) \left( A.1.N \right) \end{aligned} \right.$$

According to the syst. (A.1), the principal quadratic error $\chi_{PDA}^{2}$ to be minimized is given by the expression (A.2).

$$\chi_{PDA}^{2}=\sum_{1\leq i\leq m} \sum_{1\leq j\leq N} \left( {Lx}_{j_{i}} \right)^{2} (A.2)$$

The optimal vector of parameters $\vec{\theta}^{*}$ identification is to be performed by the least square approach as depicted by the expression (A.3).

$$\vec{\theta^{*}}=argmin\left( \chi_{PDA}^{2} \right) (A.3)$$

This statement is classically derived as result of the resolution of the syst. (A.4) [73]:

$$\left( A.4 \right)\left\{ \begin{aligned} \vec{\theta^{*}}=\arg\left( \nabla_{\vec{\theta}}\left( \chi_{PDA}^{2}(\vec{\theta^{*}}) \right)=\vec{0} \right) (A.4.1) \\ \det\left( H_{\vec{\theta}}\left( \chi_{PDA}^{2}\left( \vec{\theta^{*}} \right) \right) \right)\geq0 (A.4.2) \end{aligned} \right.$$

Where

$\nabla_{\vec{\theta}}$ is the gradient operator according to $\vec{\theta}$ components

$H_{\vec{\theta}}$ is the Hassian matrix according to $\vec{\theta}$ components

The derivative of $\chi_{PDA}^{2}$ according to the k^th^ component $\theta_{k}$ is expressed as $\nabla_{\theta_{k}}$:

$$\nabla_{\theta_{k}}=\frac{\partial\chi^{2}}{\partial\theta_{k}}=\sum_{1\leq i\leq m} \sum_{1\leq j\leq N} 2 \epsilon_{j_{i}}\frac{\partial{Lx}_{j_{i}}}{\partial\theta_{k}} \left( A.5 \right)$$

And according to (A.1):

$$\frac{\partial\chi_{PDA}^{2}}{\partial\theta_{k}}=-2\sum_{1\leq i\leq m} \sum_{1\leq j\leq N} \left( \dot{x}_{j_{i}}-f_{j}\left( \vec{x}_{i},t_{i},\vec{\theta} \right) \right)\frac{\partial\left( f_{j}\left( \vec{x}_{i},t_{i},\vec{\theta} \right) \right)}{\partial\theta_{k}} (A.6)$$

The Hessian matrix component $H_{kl}$ of $\chi_{PDA}^{2}$ according to the k^th^ and *l*^th^ components $\theta_{k}$and $\theta_{l}$ (resp.) is expressed as:

$$H_{kl}=\frac{\partial^{2}\chi_{PDA}^{2}}{\partial\theta_{l}\partial\theta_{k}}=\sum_{1\leq i\leq m} \sum_{1\leq j\leq N} \left( \frac{\partial\epsilon_{j_{i}}}{\partial\theta_{l}}\frac{\partial\epsilon_{j_{i}}}{\partial\theta_{k}}+\epsilon_{j_{i}}\frac{\partial^{2}\epsilon_{j_{i}}}{\partial\theta_{l}\partial\theta_{k}} \right) \left( A.7 \right)$$

Thus

$$H_{kl}=\frac{\partial^{2}\chi_{PDA}^{2}}{\partial\theta_{l}\partial\theta_{k}}=\sum_{1\leq i\leq m} \sum_{1\leq j\leq N} \left( \frac{\partial f_{j}\left( \vec{x}_{i},t_{i},\vec{\theta} \right)}{\partial\theta_{l}}\frac{\partial f_{j}\left( \vec{x}_{i},t_{i},\vec{\theta} \right)}{\partial\theta_{k}}-2\left( x_{j_{i}}^{'}-f_{j}\left( \vec{x}_{i},t_{i},\vec{\theta} \right) \right)\frac{\partial^{2}f_{j}\left( \vec{x}_{i},t_{i},\vec{\theta} \right)}{\partial\theta_{l}\partial\theta_{k}} \right) \left( A.8 \right)$$

1. **Case of linearity of the functions according to** $\left( \boldsymbol{\theta}_{\boldsymbol{k}} \right)_{\boldsymbol{1\leq}\boldsymbol{k}\boldsymbol{\leq}\boldsymbol{p}}$

In this section, it is proposed to consider the case of the linearity of the functions $\left( f_{j} \right)_{1\leq j\leq N}$ according to the components of the parameters vector $\vec{\theta}$. Consequently, the functions $f_{j}$ can be written as linear combinations of fitting parameters $\left( \theta_{r} \right)_{1\leq r\leq p}$ and some functions $z_{j_{r}}$ as expressed by the equation (A.9).

$$f_{j}\left( \vec{x},t,\vec{\theta} \right)=\sum_{r=1}^{p} \theta_{r} z_{j_{r}}\left( \vec{x},t \right) (A.9)$$

Where

$z_{j_{r}}\left( \vec{x},t \right)$ are functions of $\left( \vec{x},t \right)$ and are independent from the parameters $\theta_{k};$

In the case of linear least square problem, the error of estimation is convex [74], in other words:

- the next paragraphs detail the development of the least square equation according to the null gradient of the quadratic sum of errors of estimation,
- the hessian matrix is not treated.

1. **Least square equation**

Considering the independence between the $\vec{\theta}$ components, the first order derivative of $f_{j}$ according to a parameter $\theta_{k}$ is expressed as:

$$\frac{\partial f_{j}}{\partial\theta_{k}}\left( \vec{x},t,\vec{\theta} \right)=z_{j_{k}}\left( \vec{x},t \right) (A.10)$$

Hence, the expression (A.6) becomes:

$$\frac{\partial\chi_{PDA}^{2}}{\partial\theta_{k}}=-2\sum_{1\leq i\leq m} \sum_{1\leq j\leq N} \left( x_{j_{i}}^{'} z_{j_{k}}\left( \vec{x}_{i},t_{i} \right) \right)+2\sum_{1\leq i\leq m} \left( z_{j_{k}}\left( \vec{x}_{i},t_{i} \right)\sum_{1\leq r\leq p} \theta_{r}z_{j_{r}}\left( \vec{x}_{i},t_{i} \right) \right) \left( A.11 \right)$$

Considering the optimality criterion that is presented by the equation (6.1) at the points $\vec{\theta}=\left( \theta_{1}^{*},\ldots,\theta_{p}^{*} \right)^{t}$:

$\forall k\in\left\{ 1,\ldots,p \right\}$

$$\frac{\partial\chi_{PDA}^{2}}{\partial\theta_{k}}\left( \left( \theta_{1}^{*},\ldots,\theta_{p}^{*} \right)^{t} \right)=0$$

$$\Leftrightarrow\sum_{1\leq i\leq m} \sum_{1\leq j\leq N} \left( z_{j_{k}}\left( \vec{x}_{i},t_{i} \right)\sum_{1\leq r\leq p} \theta_{r}^{*} z_{j_{r}}\left( \vec{x}_{i},t_{i} \right) \right)=\sum_{1\leq i\leq m} \sum_{1\leq j\leq N} \left( x_{j_{i}}^{'} z_{j_{k}}\left( \vec{x}_{i},t_{i} \right) \right)$$

by matrix representation:

$$\left( \theta_{1}^{*},\ldots,\theta_{p}^{*} \right)\binom{\sum_{1\leq i\leq m} \sum_{1\leq j\leq N} \left( z_{j_{k}}\left( \vec{x}_{i},t_{i} \right)z_{j_{1}}\left( \vec{x}_{i},t_{i} \right) \right)}{\begin{aligned} \vdots\\ \sum_{1\leq i\leq m} \sum_{1\leq j\leq N} \left( z_{j_{k}}^{2}\left( \vec{x}_{i},t_{i} \right) \right) \\ \vdots\\ \sum_{1\leq i\leq m} \sum_{1\leq j\leq N} \left( z_{j_{k}}\left( \vec{x}_{i},t_{i} \right)z_{j_{p}}\left( \vec{x}_{i},t_{i} \right) \right) \end{aligned}}=\sum_{1\leq i\leq m} \sum_{1\leq j\leq N} \left( x_{j_{i}}^{'} z_{j_{k}}\left( \vec{x}_{i},t_{i} \right) \right) \left( A.12 \right)$$

So for all $k\in\left\{ 1,\ldots,p \right\}$, the assembly matrix of the optimality criterion is to be expressed by the expression (A.13).

$$\left( \begin{matrix} \sum_{1\leq i\leq m} \sum_{1\leq j\leq N} z_{j_{1}}^{2}\left( \vec{x}_{i},t_{i} \right) & \cdots& \sum_{1\leq i\leq m} \sum_{1\leq j\leq N} \left( z_{j_{1}}\left( \vec{x}_{i},t_{i} \right)z_{j_{p}}\left( \vec{x}_{i},t_{i} \right) \right) \\ \vdots& \ddots& \vdots\\ \sum_{1\leq i\leq m} \sum_{1\leq j\leq N} \left( z_{j_{1}}\left( \vec{x}_{i},t_{i} \right)z_{j_{p}}\left( \vec{x}_{i},t_{i} \right) \right) & \cdots& \sum_{1\leq i\leq m} \sum_{1\leq j\leq N} z_{j_{p}}^{2}\left( \vec{x}_{i},t_{i} \right) \end{matrix} \right)\left( \begin{aligned} \theta_{1}^{*} \\ \vdots\\ \theta_{k}^{*} \\ \vdots\\ \theta_{p}^{*} \end{aligned} \right)=\left( \begin{aligned} \sum_{1\leq i\leq m} \sum_{1\leq j\leq N} \left( x_{j_{i}}^{'} z_{j_{1}}\left( \vec{x}_{i},t_{i} \right) \right) \\ \vdots\\ \sum_{1\leq i\leq m} \sum_{1\leq j\leq N} \left( x_{j_{i}}^{'} z_{j_{k}}\left( \vec{x}_{i},t_{i} \right) \right) \\ \vdots\\ \sum_{1\leq i\leq m} \sum_{1\leq j\leq N} \left( x_{j_{i}}^{'} z_{j_{p}}\left( \vec{x}_{i},t_{i} \right) \right) \end{aligned} \right) (A.13)$$

Hence, the least square criteria is equivalent to the resolution of the equation (A.14):

$$\left( A.14 \right)\left\{ \begin{aligned} \left[ A \right]\vec{\theta}=\vec{b} \\ or \\ a_{kl} \theta_{k}=b_{k} \end{aligned} \right.$$

Where

$$\left( A.15 \right)\left\{ \begin{aligned} a_{kl}=\sum_{1\leq i\leq m} \sum_{1\leq j\leq N} \left( z_{j_{k}}\left( \vec{x}_{i},t_{i} \right) z_{j_{l}}\left( \vec{x}_{i},t_{i} \right) \right) \\ b_{k}=\sum_{1\leq i\leq m} \sum_{1\leq j\leq N} \left( x_{j_{i}}^{'} z_{j_{k}}\left( \vec{x}_{i},t_{i} \right) \right) \end{aligned} \right.$$

The matrix [A] is symmetrical and its reversibility depends on the problem; in other term, the uniqueness of the parameters vector $\vec{\theta}$ depends on the resolution of the system (A.14) in term of [A] matrix singularity, reversibility, over-determined solution, etc.

1. **Adaptating to the SIR model**

In this section, the equation (A.13) is adapted to the SIR model as it is described by system (1) referred in section 1 of this paper.

$$\left( 1 \right)\left\{ \begin{aligned} \frac{di\left( t \right)}{dt}=\beta i\left( t \right)s\left( t \right)-\mu i\left( t \right) (1.1) \\ \frac{ds\left( t \right)}{dt}=-\beta i\left( t \right)s\left( t \right) (1.2) \end{aligned} \right.$$

According to the notation of the system (1), the equations (46.1) and (46.2) should be written in the following form:

$$(A.16)\left\{ \begin{aligned} \dot{x_{1}}\left( t \right)=f_{1}\left( \left( i\left( t \right),s\left( t \right) \right)^{T},t,\left( \beta,\mu\right)^{T} \right) (A.16.1) \\ \dot{x_{2}}\left( t \right)=f_{2}\left( \left( i\left( t \right),s\left( t \right) \right)^{T},t,\left( \beta,\mu\right)^{T} \right) (A.16.2) \end{aligned} \right.$$

Where

$$\left( A.17 \right)\left\{ \begin{aligned} \vec{x}\left( t \right)=\left( x_{1}\left( t \right) \right)^{T}=\left( i\left( t \right),s\left( t \right) \right)^{T} \\ \left\{ \begin{aligned} f_{1}\left( \vec{x}\left( t \right),t,\vec{\theta} \right)=\beta x_{1}\left( t \right)x_{2}\left( t \right)-\mu x_{1}\left( t \right) \\ f_{2}\left( \vec{x}\left( t \right),t,\vec{\theta} \right)=-\beta x_{1}\left( t \right)x_{2}\left( t \right) \end{aligned} \right. \\ \vec{\theta}=\left( \beta,\mu\right)^{T} \end{aligned} \right.$$

According to the notation (A.9) we consider the following parameterization:

- The vector of parameters of the ODE system (A.16):

$$\left( A.18 \right)\left\{ \begin{aligned} \left( A.18.1 \right)\left\{ \begin{aligned} \theta_{1}=\beta\\ \theta_{2}=\mu\end{aligned} \right. \\ \left( A.18.2 \right)\left\{ \begin{aligned} z_{1_{1}}\left( t \right)=x_{1}\left( t \right)x_{2}\left( t \right)=i\left( t \right)s\left( t \right) \\ z_{1_{2}}\left( t \right)=-x_{1}\left( t \right)=-i\left( t \right) \\ z_{2_{1}}\left( t \right)=-x_{1}\left( t \right)x_{2}\left( t \right)=-i\left( t \right)s\left( t \right) \\ z_{2_{2}}\left( t \right)=0 \end{aligned} \right. \end{aligned} \right.$$

The limits of the indices are:

$$\left( A.19 \right) \left\{ \begin{aligned} N=2 \\ p=2 \end{aligned} \right.$$

Thus,

$$\left( \begin{matrix} \sum_{1\leq k\leq m} z_{{1_{1}}_{k}}^{2}+z_{{2_{1}}_{k}}^{2} & \sum_{1\leq k\leq m} \left( {z_{1_{1}}}_{k}{z_{1_{2}}}_{k}+{z_{2_{1}}}_{k}{z_{2_{2}}}_{k} \right) \\ \sum_{1\leq k\leq m} \left( {z_{1_{1}}}_{k}{z_{1_{2}}}_{k}+{z_{2_{1}}}_{k}{z_{2_{2}}}_{k} \right) & \sum_{1\leq k\leq m} z_{{1_{2}}_{k}}^{2}+z_{{2_{2}}_{k}}^{2} \end{matrix} \right)\left( \begin{aligned} \theta_{1}^{*} \\ \theta_{2}^{*} \end{aligned} \right)=\left( \begin{aligned} \sum_{1\leq k\leq m} \left( x_{1_{k}}^{'}{z_{1_{1}}}_{k}+x_{2_{k}}^{'}{z_{2_{1}}}_{k} \right) \\ \sum_{1\leq k\leq m} \left( x_{1_{k}}^{'}{z_{1_{2}}}_{k}+x_{2_{k}}^{'}{z_{2_{2}}}_{k} \right) \end{aligned} \right) \left( A.20 \right)$$

Where $x_{1_{k}}^{'}$ and $x_{2_{k}}^{'}$ are the numerical derivative of the observation, resp. $i_{k}$ and $s_{k}$, and they are to be computed using the expressions (A.21.1) and (A.21.2). The index k is related to the observation index.

$$\left( A.21 \right) \left\{ \begin{aligned} {\dot{x_{1}}}_{k}=\frac{i_{k+1}-i_{k}}{\tau} \left( A.21.1 \right) \\ {\dot{x_{2}}}_{k}=\frac{s_{k+1}-s_{k}}{\tau} \left( A.21.2 \right) \end{aligned} \right.$$

The time-step $\tau$ is to be chosen could be set according to the approach is each specialist or researcher. In this work, $\tau$ was set to 1 day and it is a dimensionless parameter.

Hence according to the expressions (A.18.2), the equation (A.20) becomes:

$$\left( \begin{matrix} \sum_{1\leq k\leq m} 2 \left( i_{k}s_{k} \right)^{2} & \sum_{1\leq k\leq m} -i_{k}^{2}s_{k} \\ \sum_{1\leq k\leq m} -i_{k}^{2}s_{k} & \sum_{1\leq k\leq m} i_{k}^{2} \end{matrix} \right)\left( \begin{aligned} \beta^{*} \\ \mu^{*} \end{aligned} \right)=\left( \begin{aligned} \sum_{1\leq k\leq m-1} \left( \frac{i_{k+1}-i_{k}}{\tau}i_{k}s_{k}-\frac{s_{k+1}-s_{k}}{\tau}i_{k}s_{k} \right) \\ \sum_{1\leq k\leq m-1} \left( -\frac{i_{k+1}-i_{k}}{\tau}i_{k} \right) \end{aligned} \right) \left( A.22 \right)$$

Hence, the optimized parameters are to be computed according to the expression (A.23).

$$\left( \begin{aligned} \beta^{*} \\ \mu^{*} \end{aligned} \right)=\left[ A \right]^{-1} \vec{b} (A.23)$$

Where

$$\left( A.24 \right)\left\{ \begin{aligned} \left[ A \right]=\left( \begin{matrix} \sum_{1\leq k\leq m} 2 \left( i_{k}s_{k} \right)^{2} & \sum_{1\leq k\leq m} -i_{k}^{2}s_{k} \\ \sum_{1\leq k\leq m} -i_{k}^{2}s_{k} & \sum_{1\leq k\leq m} i_{k}^{2} \end{matrix} \right) \left( A.24.1 \right) \\ \vec{b}=\left( \begin{aligned} \sum_{1\leq k\leq m-1} \left( \frac{i_{k+1}-i_{k}}{\tau}i_{k}s_{k}-\frac{s_{k+1}-s_{k}}{\tau}i_{k}s_{k} \right) \\ \sum_{1\leq k\leq m-1} \left( -\frac{i_{k+1}-i_{k}}{\tau}i_{k} \right) \end{aligned} \right) \left( A.24.2 \right) \\ \tau=1 \left( A.24.3 \right) \end{aligned} \right.$$

1. **Errors of estimation and PDA error expressions**

Two types of fitting errors were analyzed:

- the first corresponds to the error of estimation. As described by the system (2), the system (A.25) described the errors ${\epsilon_{1}}_{k}$ and ${\epsilon_{2}}_{k}$at each point $t_{k}$ that are to be computed for each of the functions $\tilde{i}$ and $\tilde{s}$.

$$(A.25)\left\{ \begin{aligned} {\epsilon_{1}}_{k}=\tilde{i}\left( t_{k} \right)-i_{k}=\tilde{x}_{1}\left( t_{k} \right)-{x_{1}}_{k} \\ {\epsilon_{2}}_{k}=\tilde{s}\left( t_{i} \right)-s_{k}=\tilde{x}_{2}\left( t_{k} \right)-{x_{2}}_{k} \end{aligned} \right.$$

After the errors computation, the normality test is to be performed on the random variables $\epsilon_{1}$ and $\epsilon_{2}$.
