# Supplementary figures and images for: Socio-economic analysis of short-term trends of COVID-19: modeling and data analytics
Source: BMC Public Health. 2022 Aug 29;22:1633. doi: 10.1186/s12889-022-13788-4 (PMC9421639; doi:10.1186/s12889-022-13788-4)

**Additional file 2: Plot of the observed** **and the simulated infection rates**


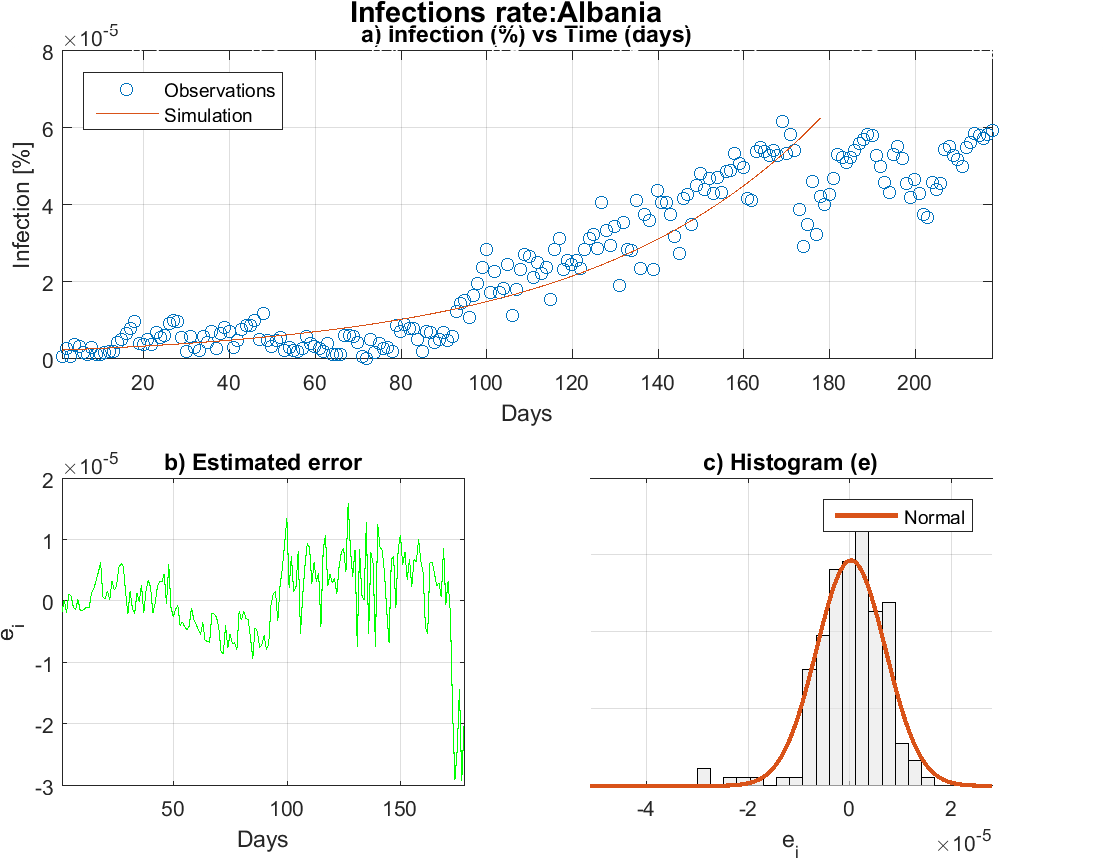


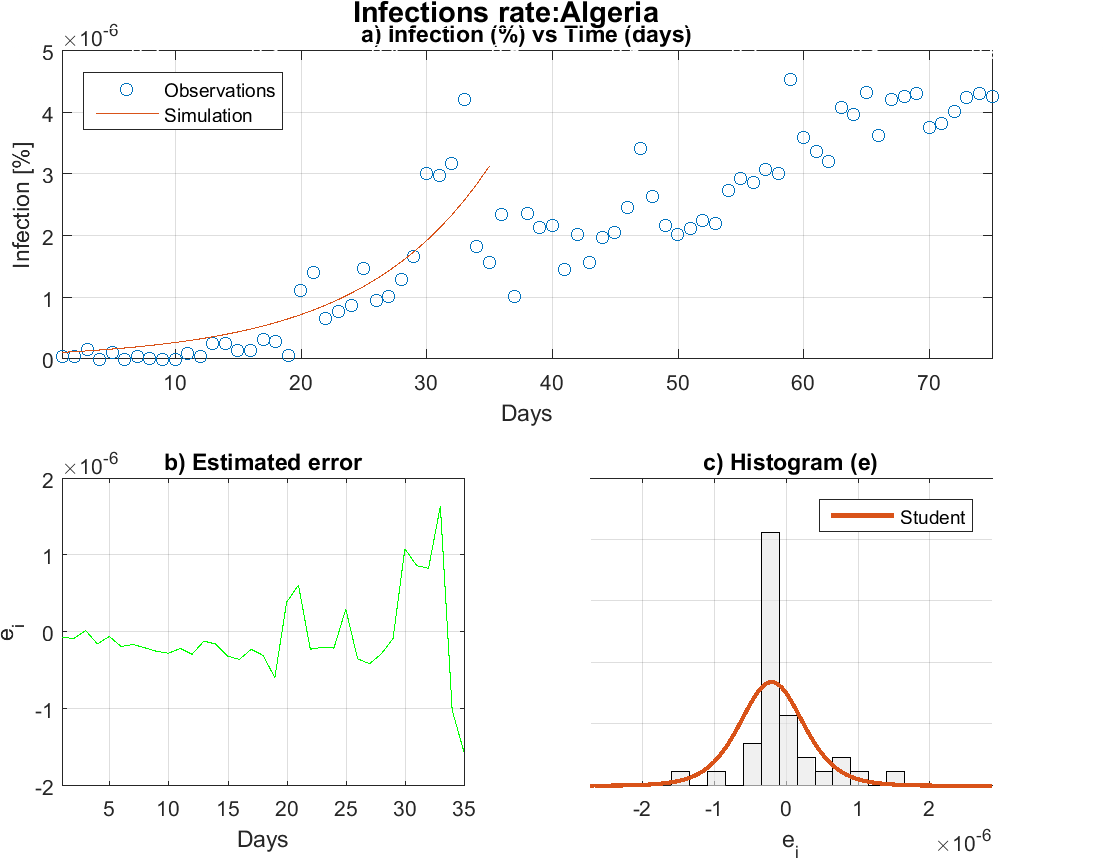


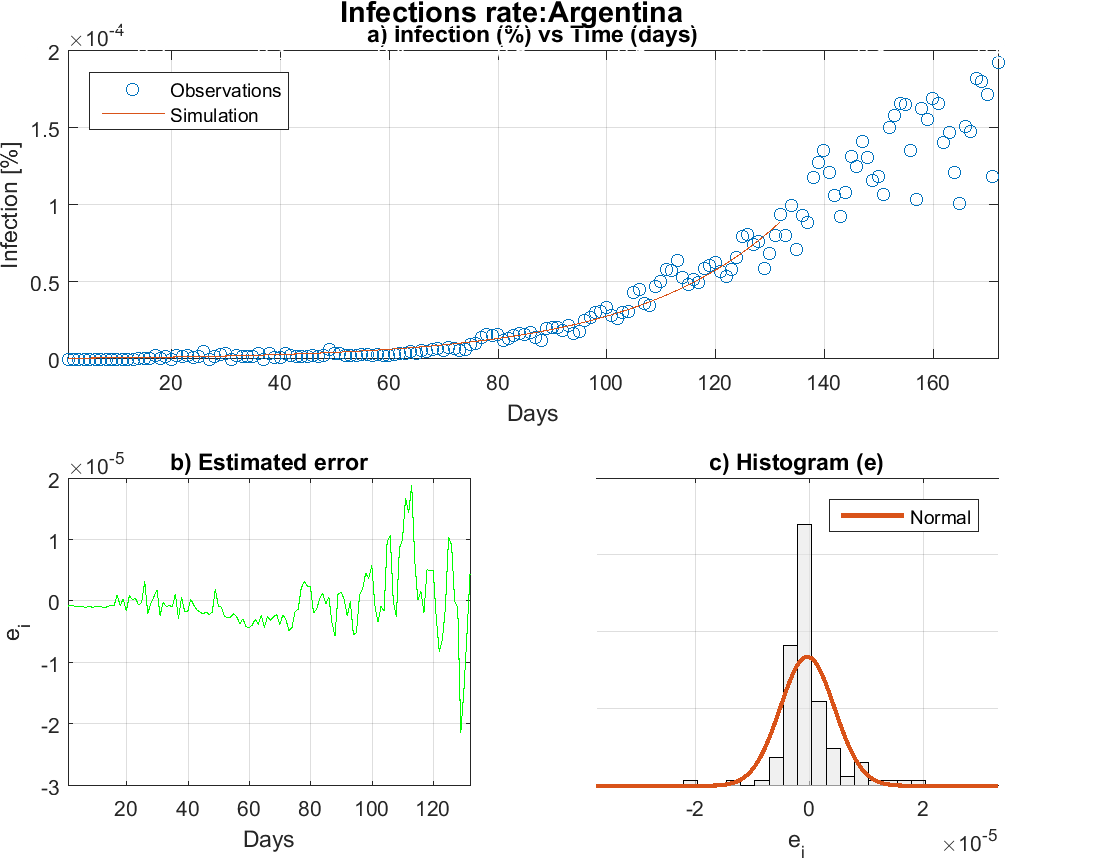


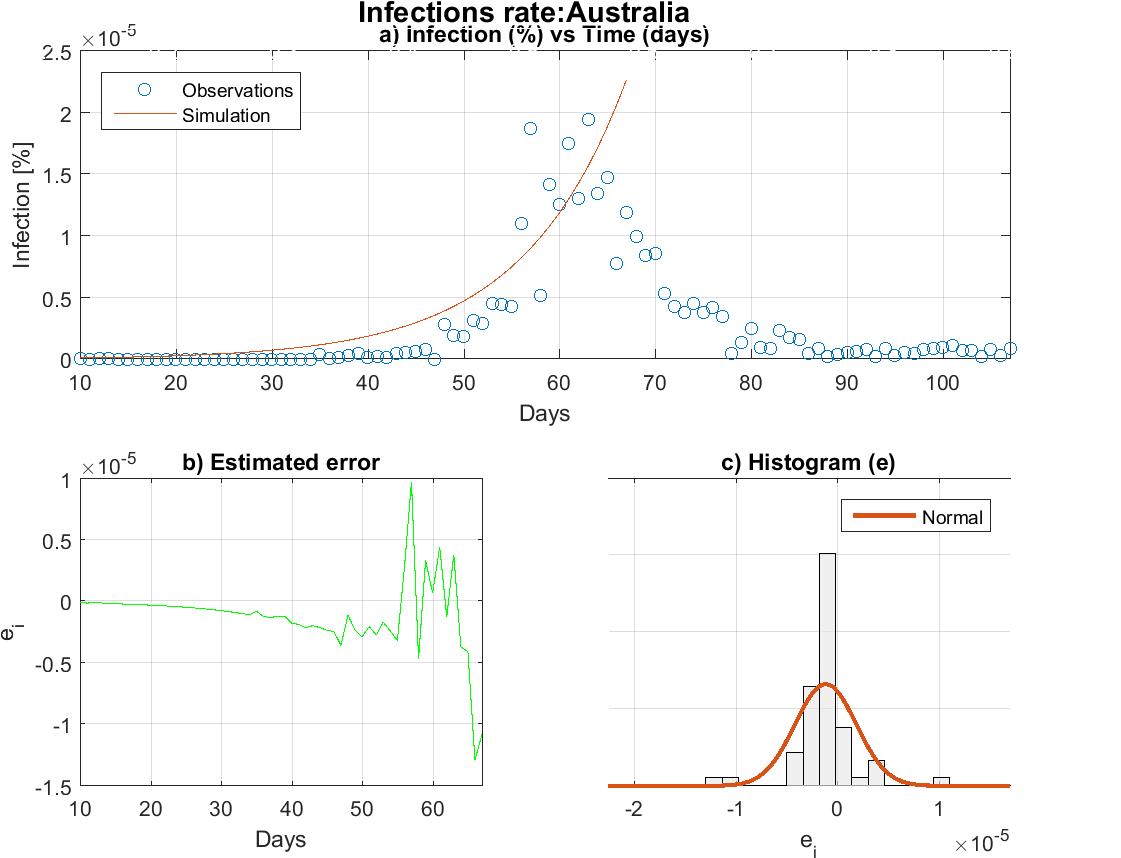


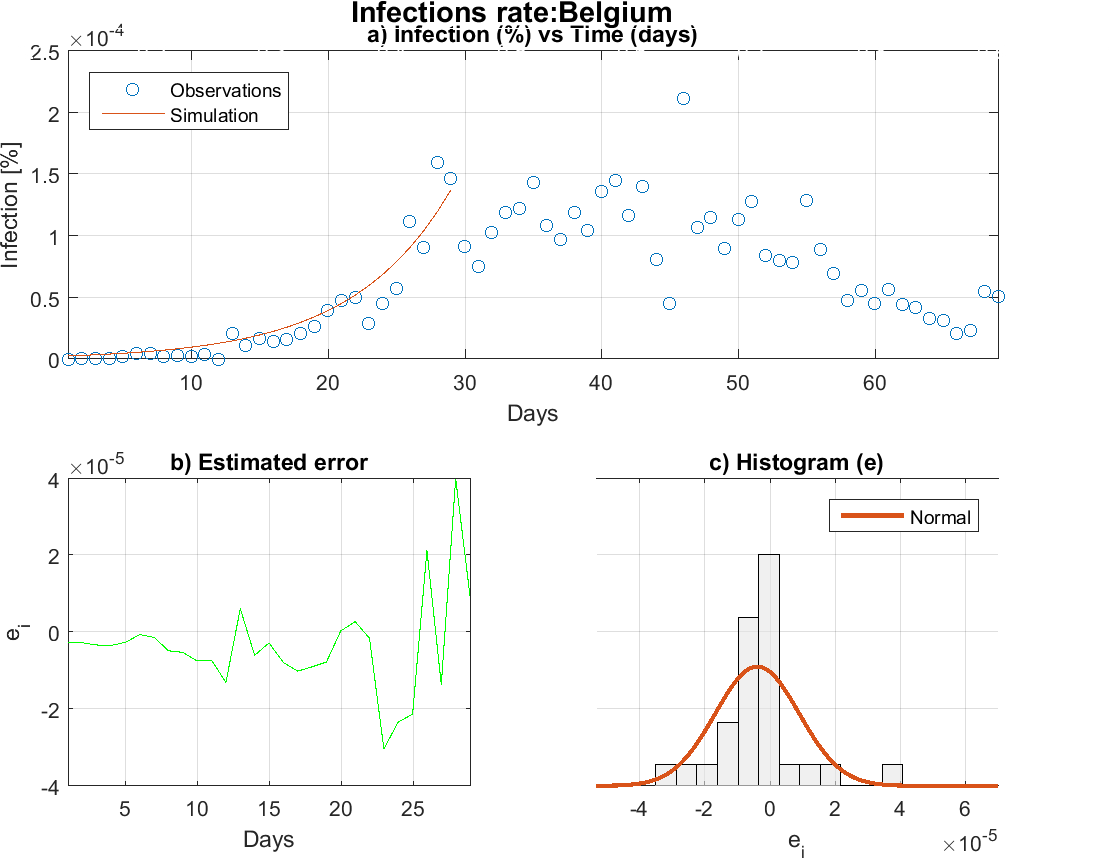


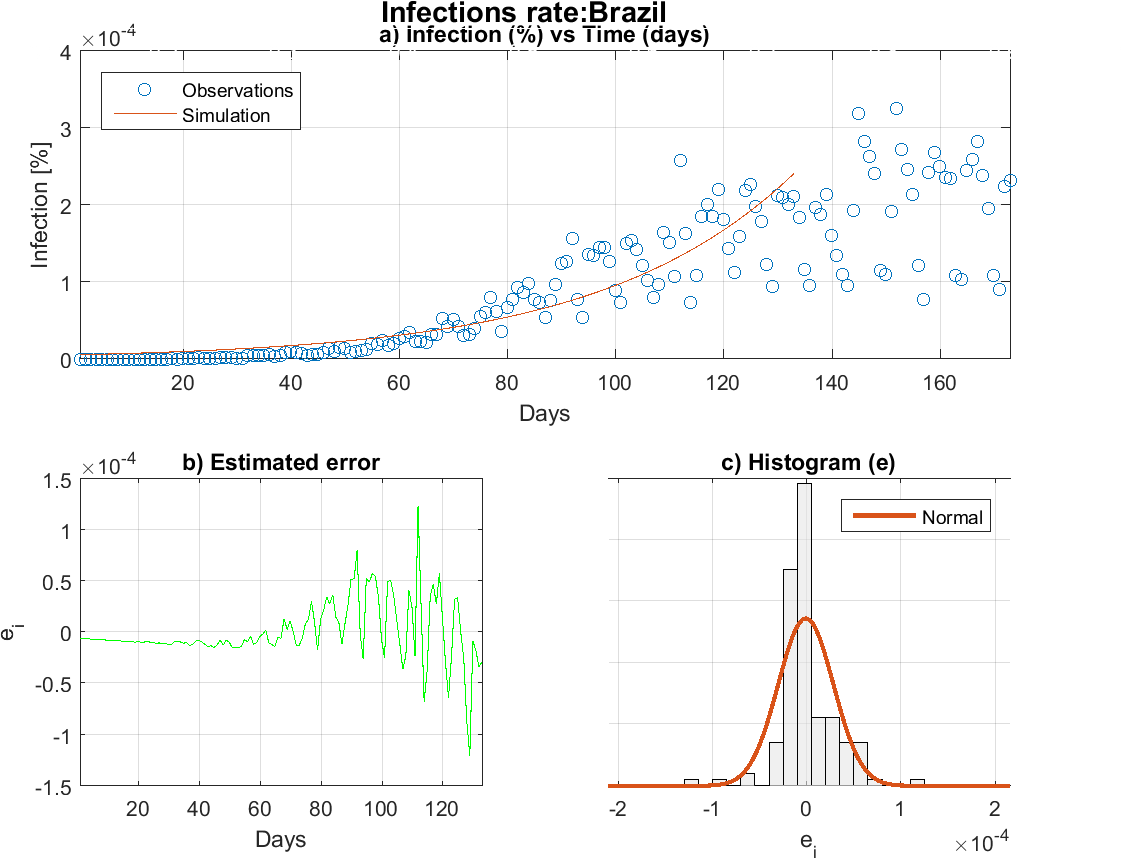


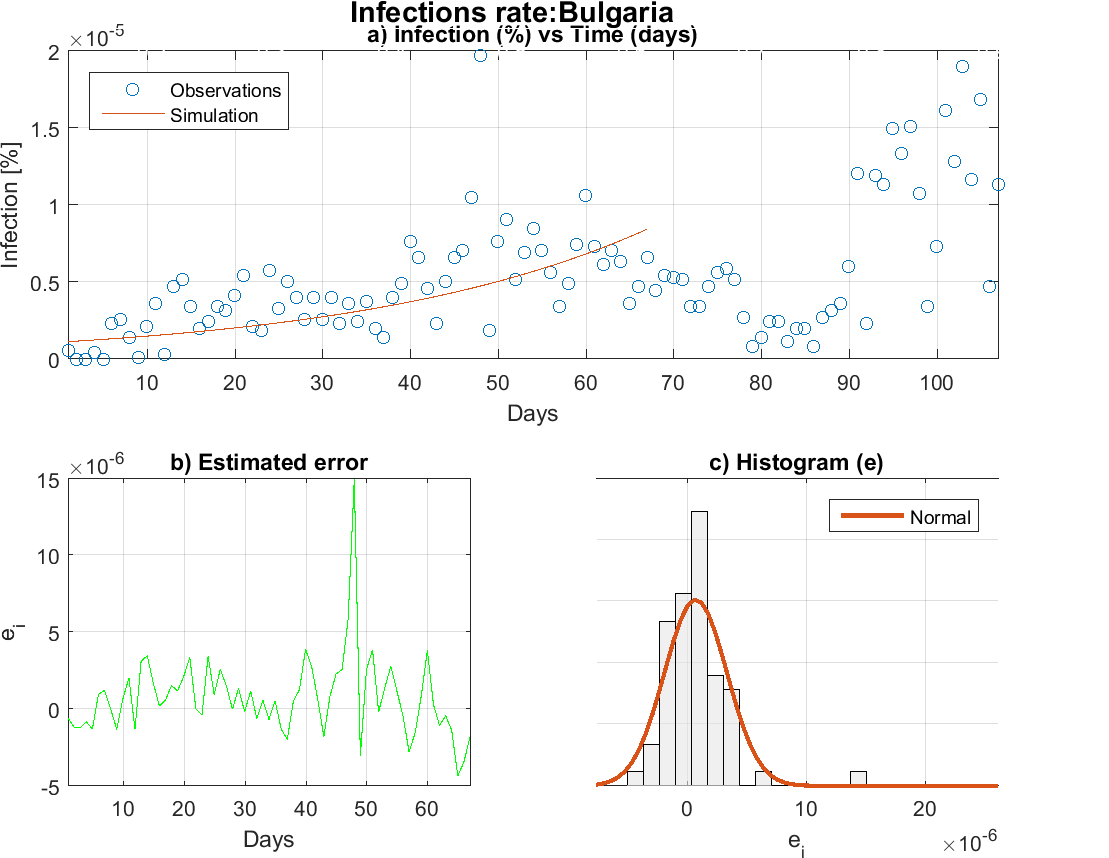


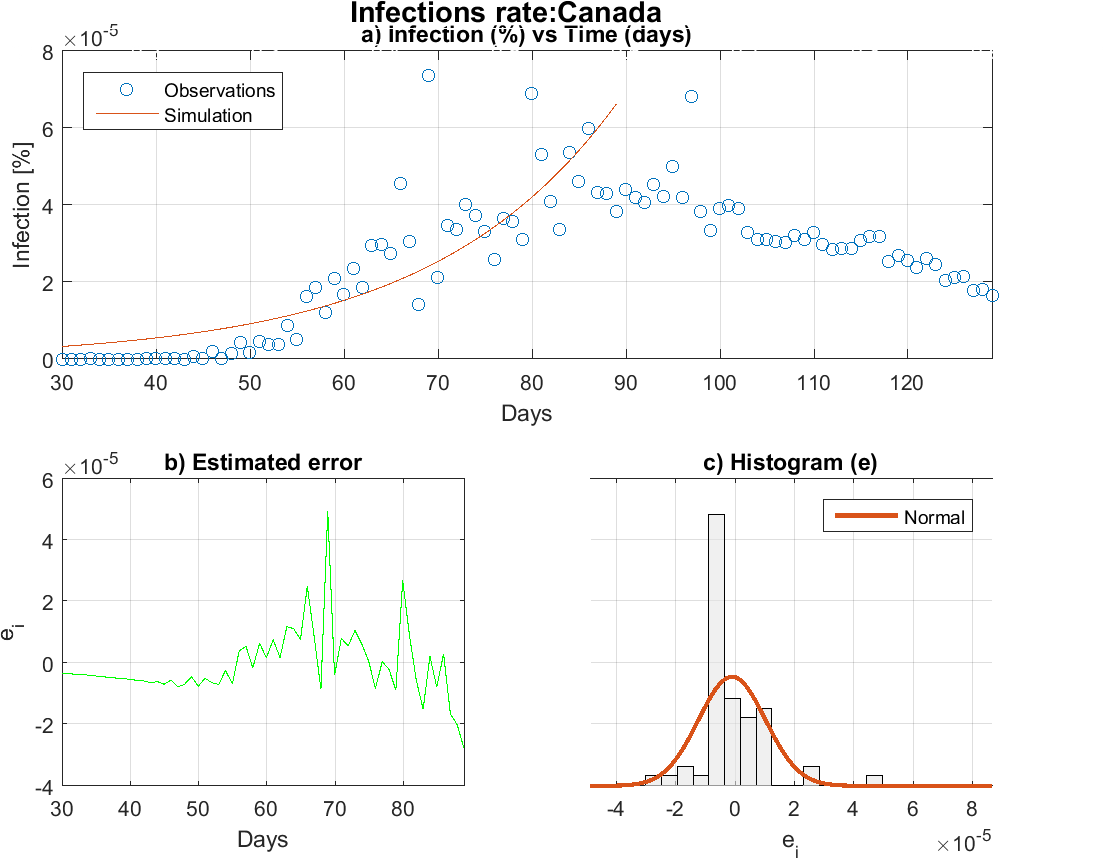


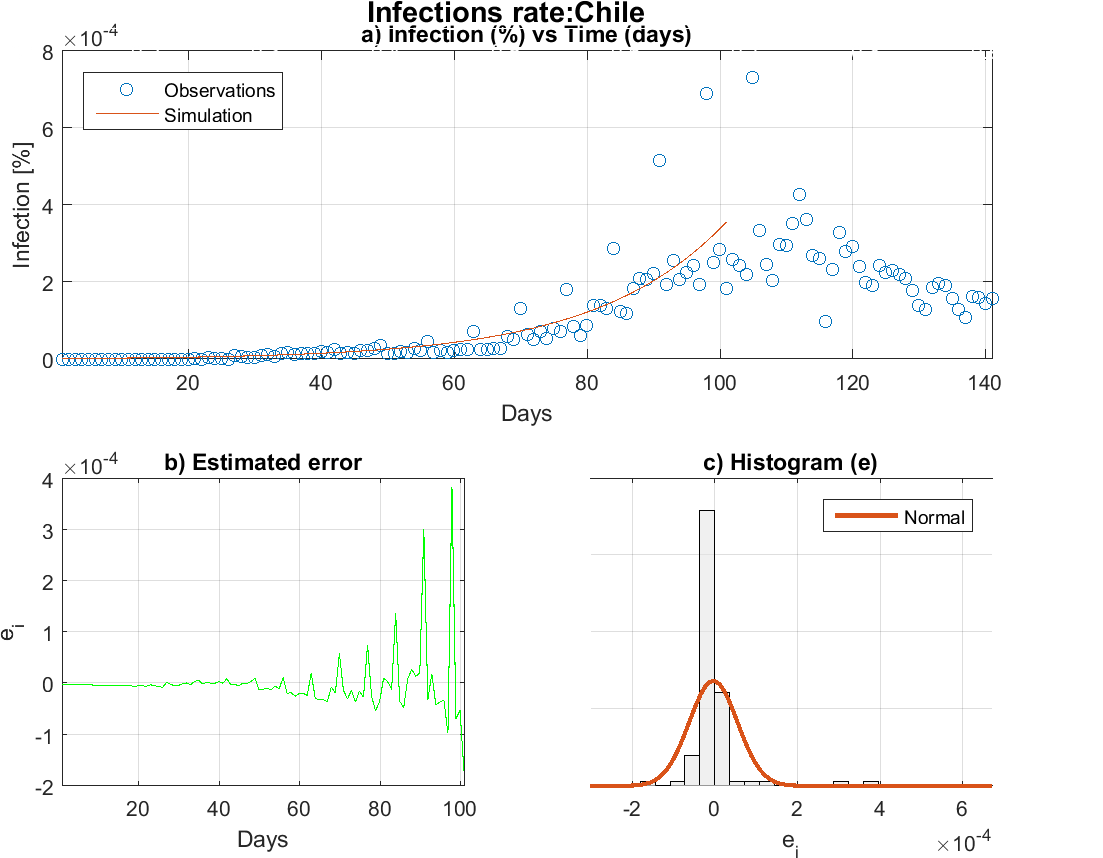


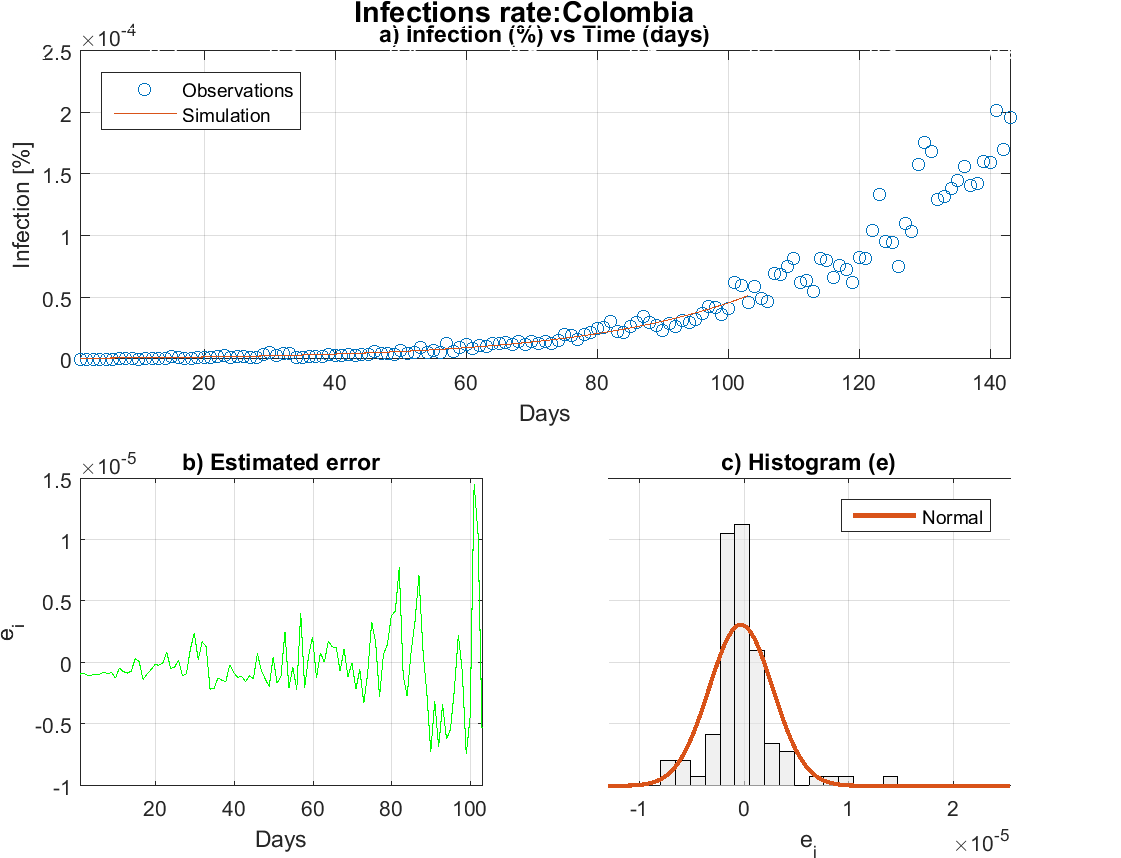


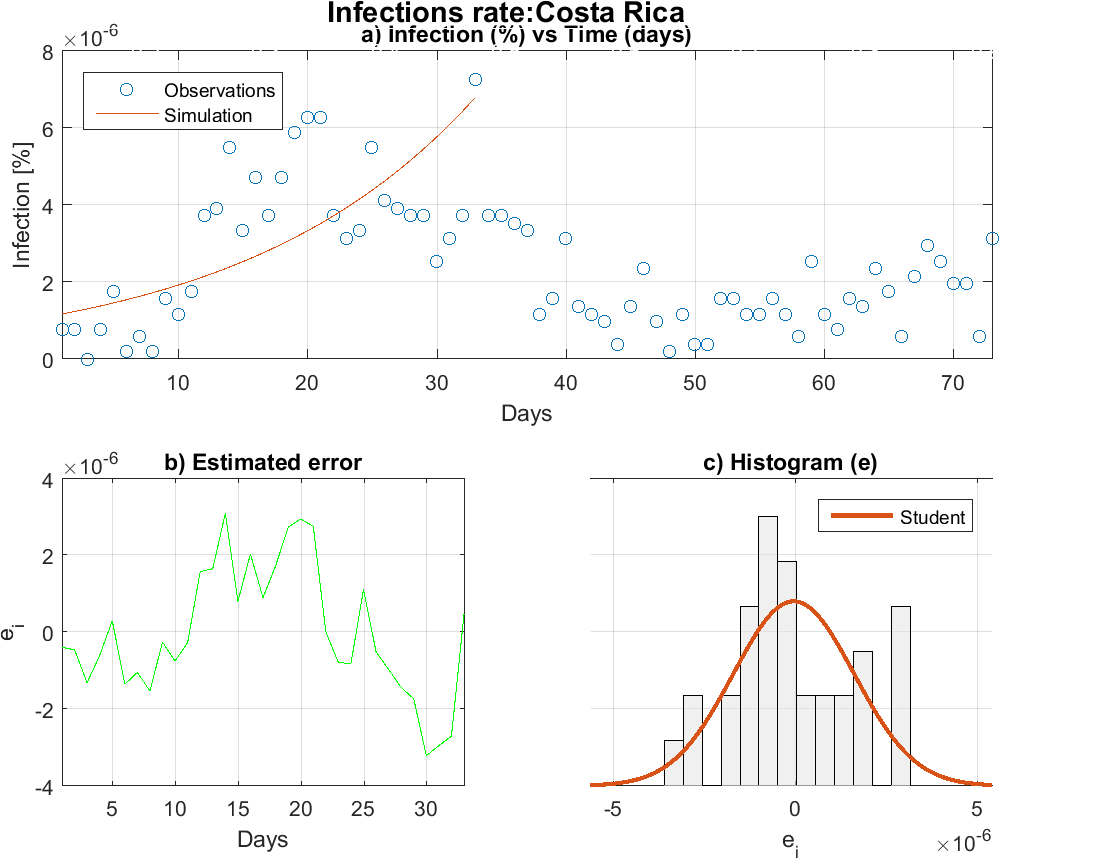


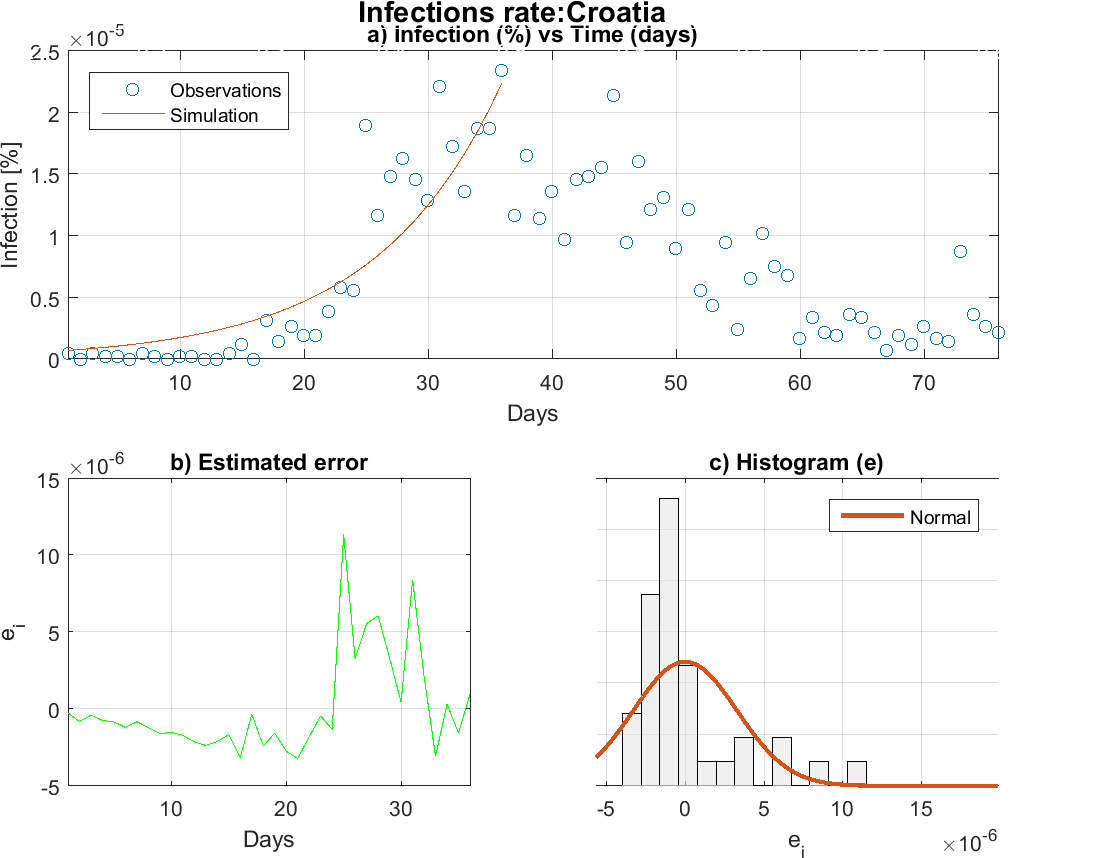


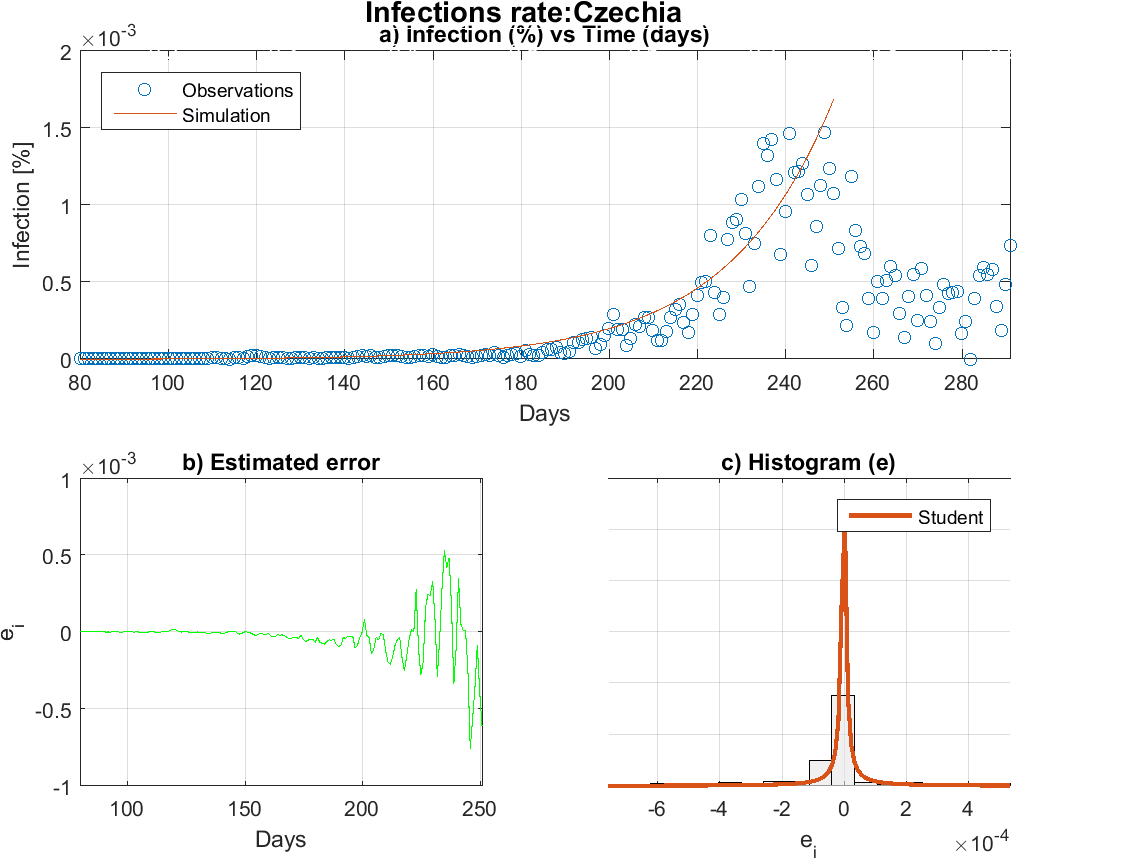


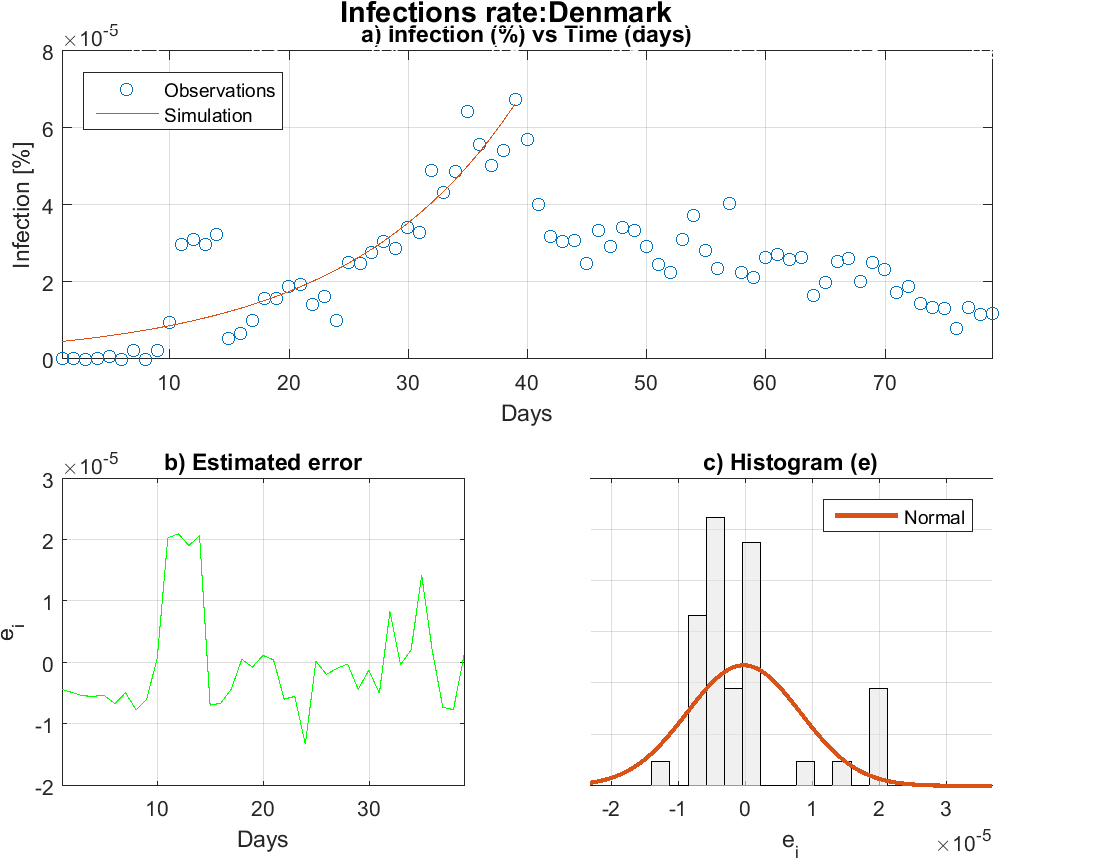


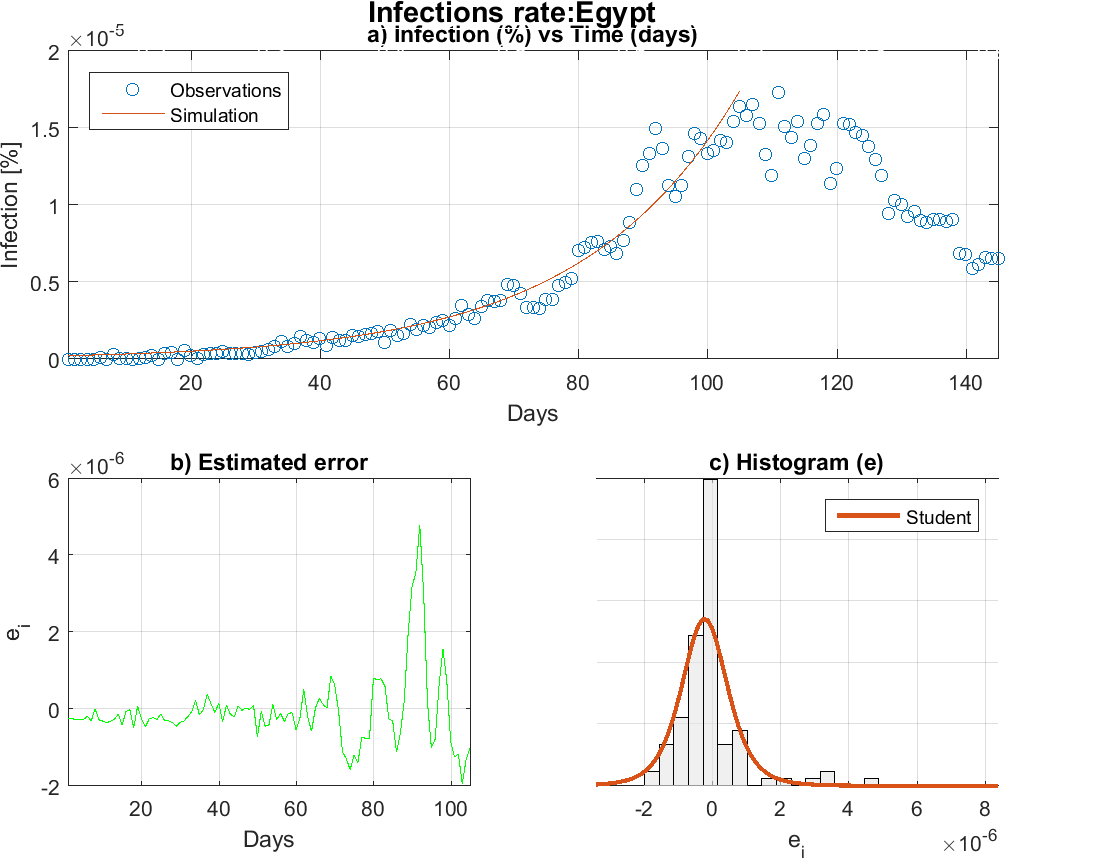


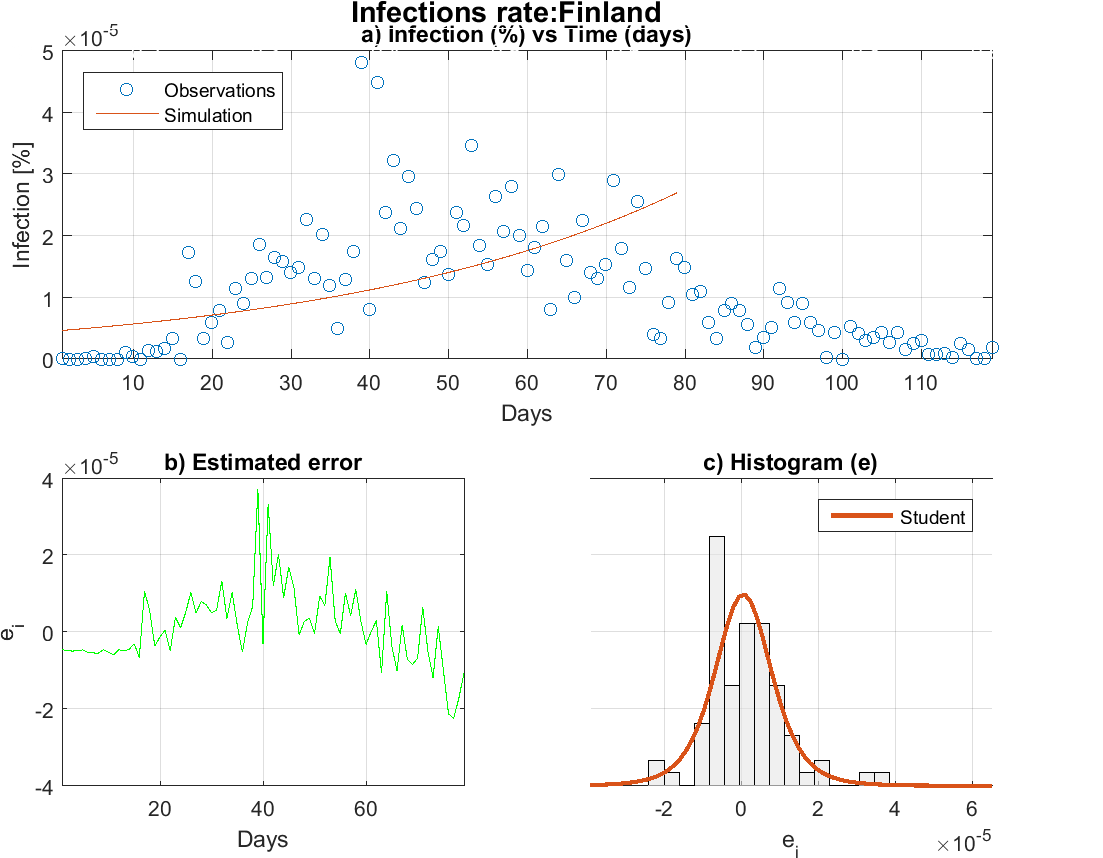


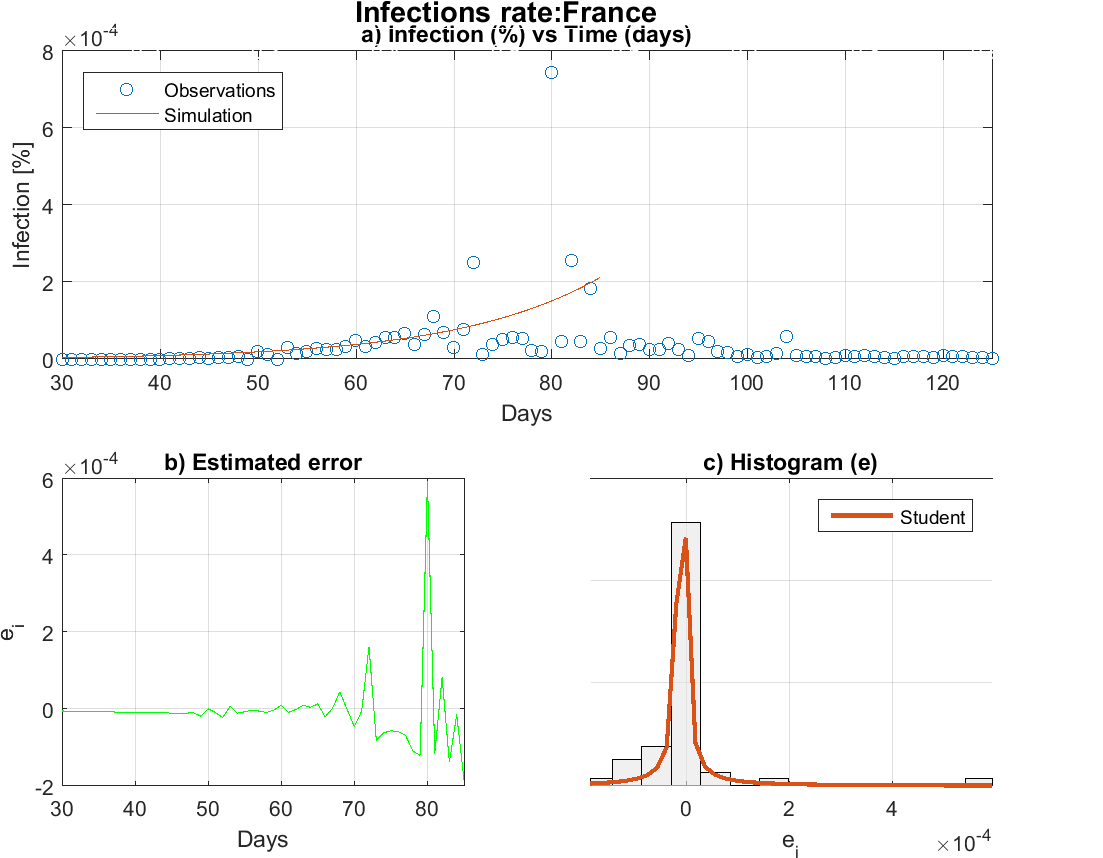


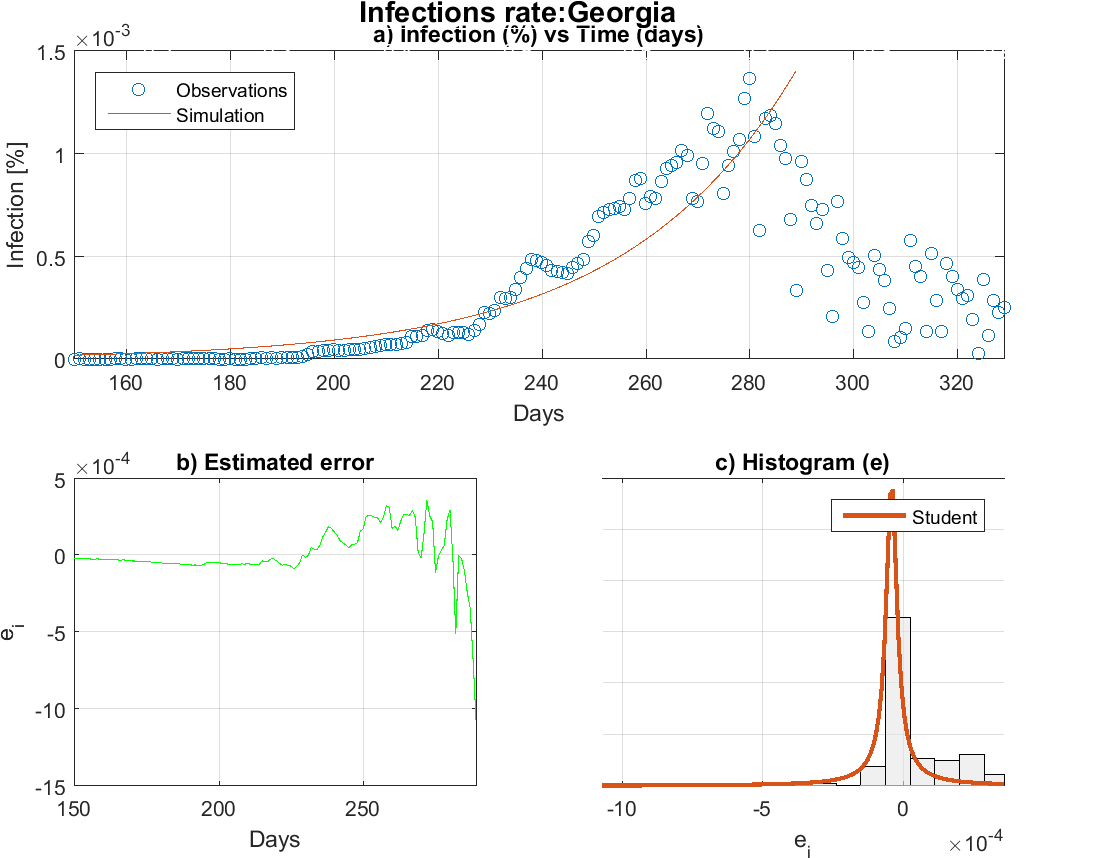


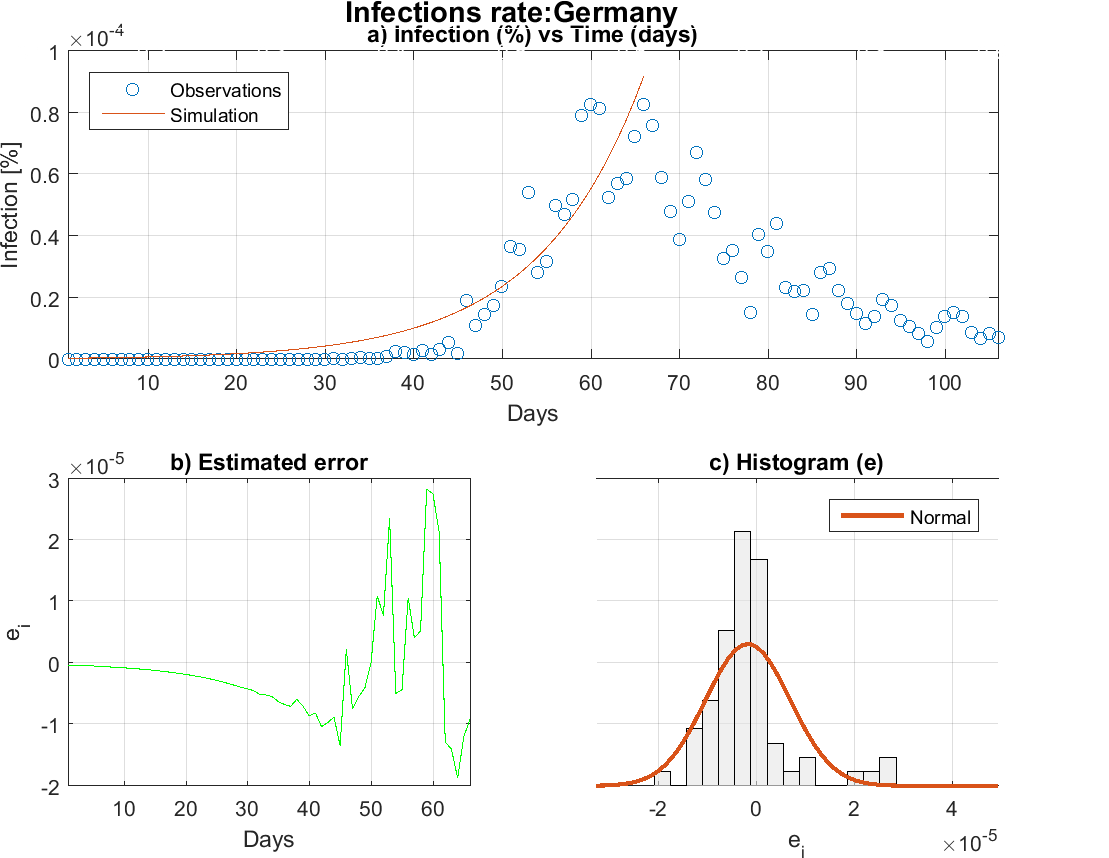


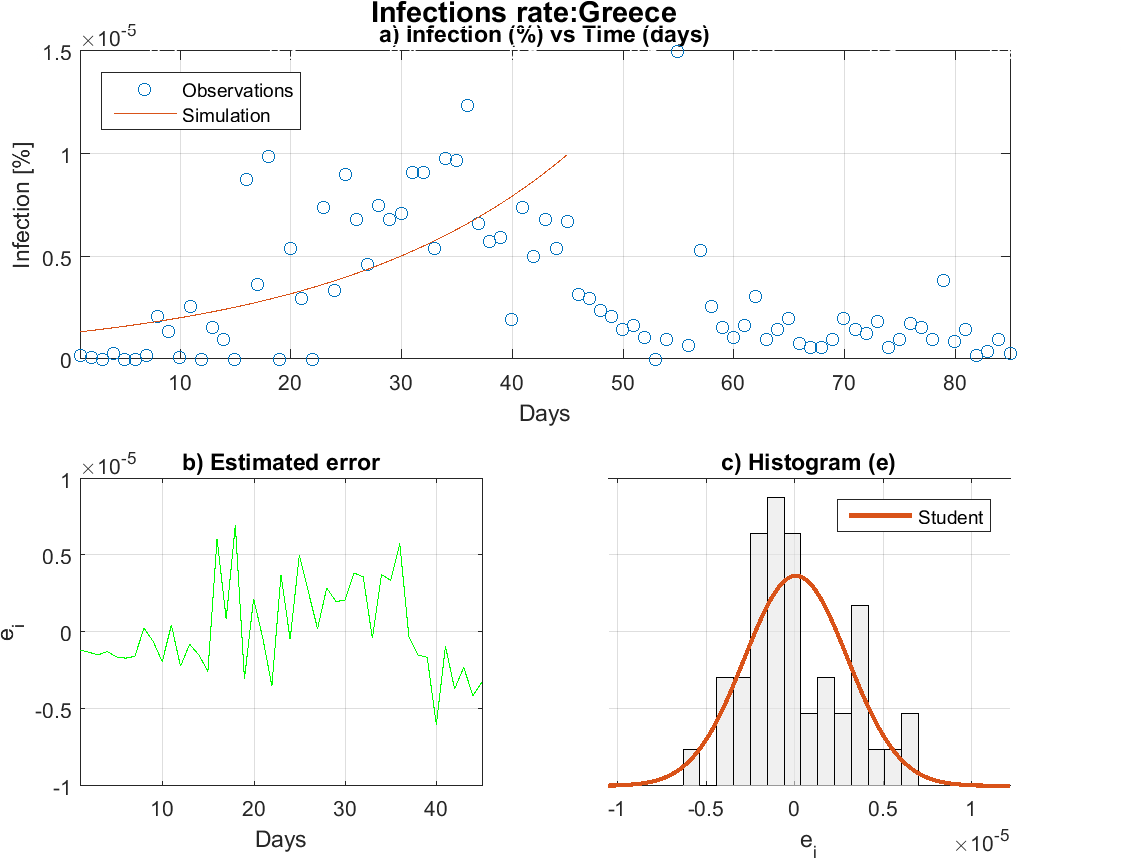


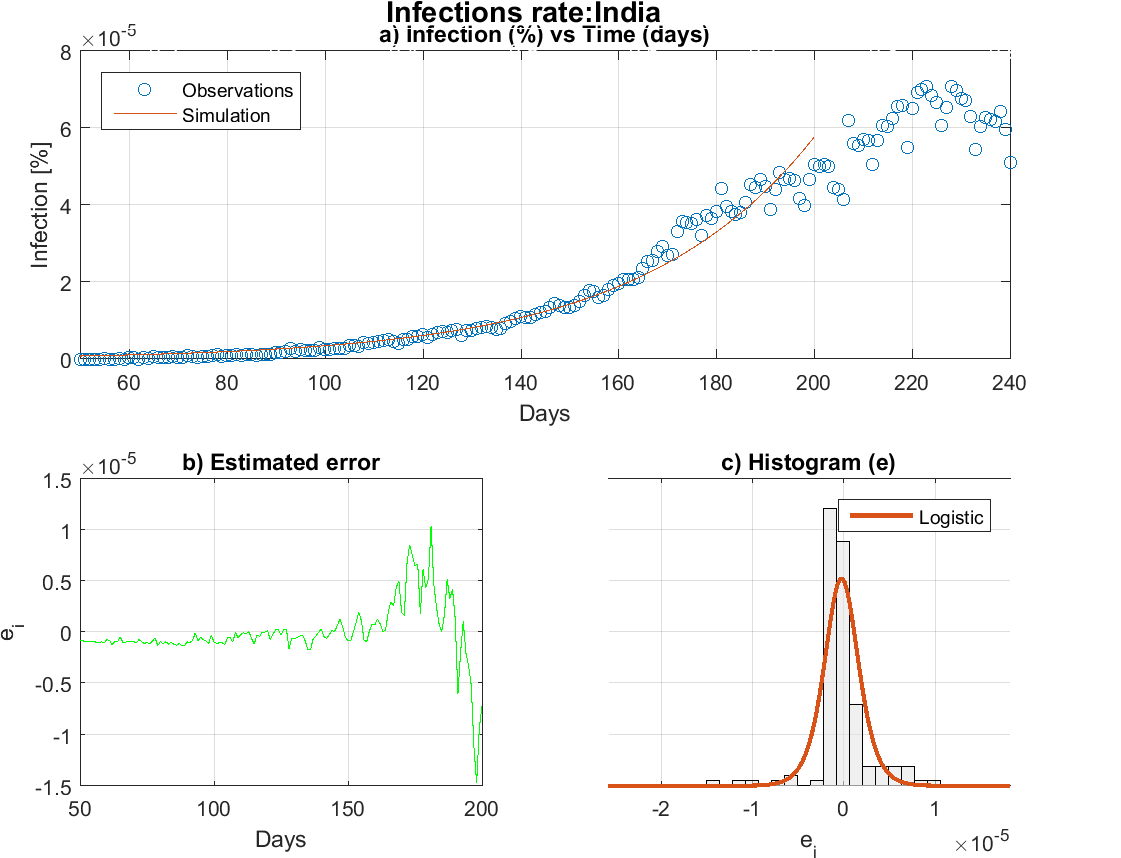


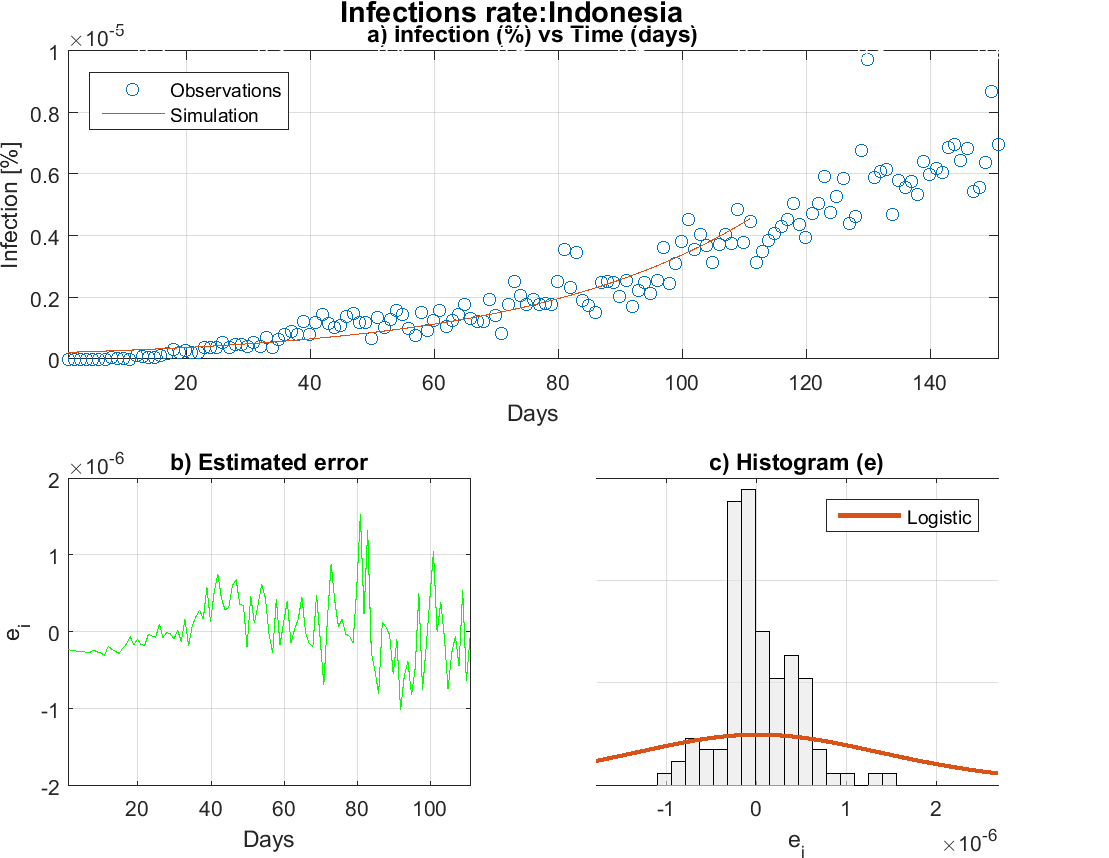


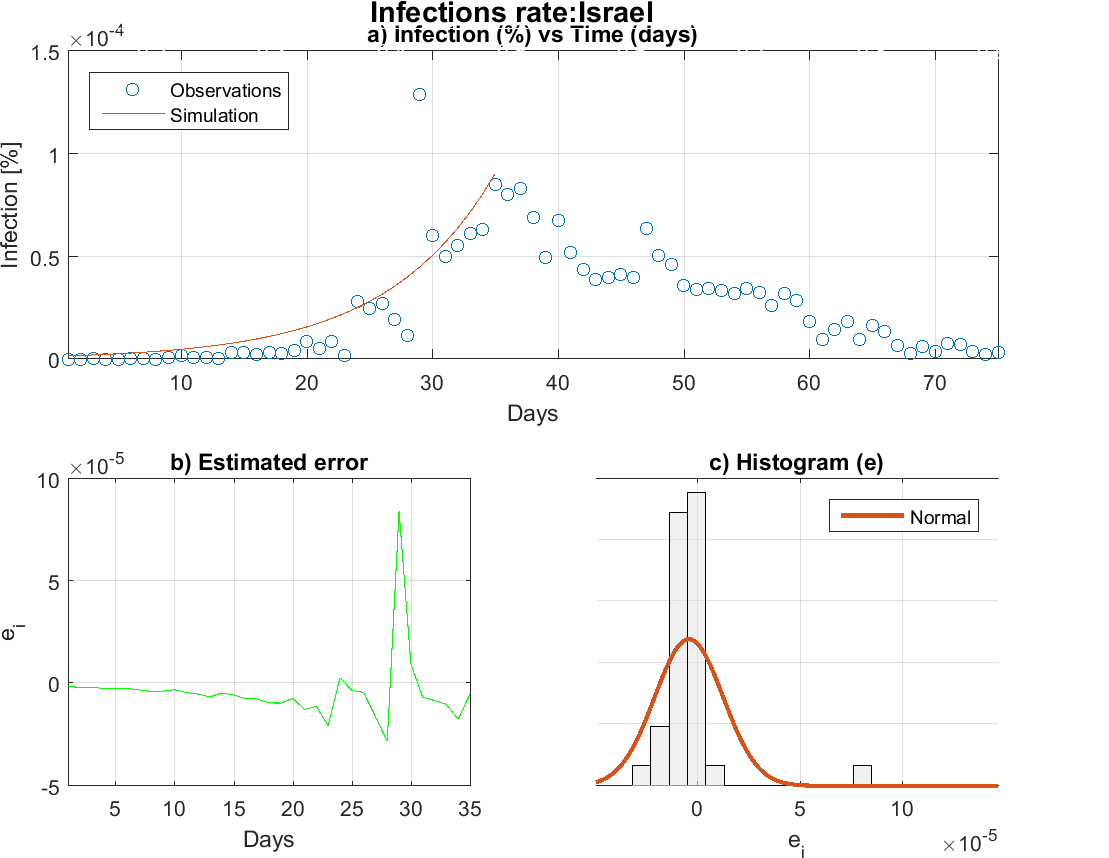


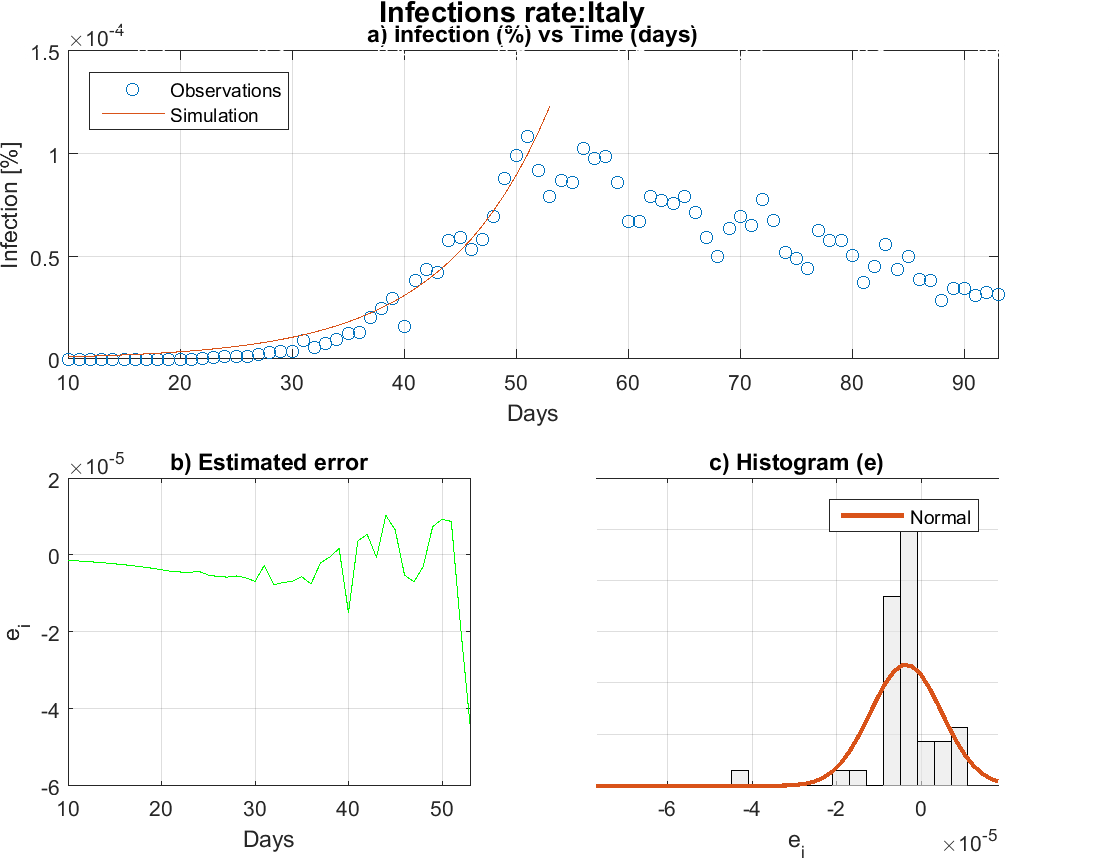


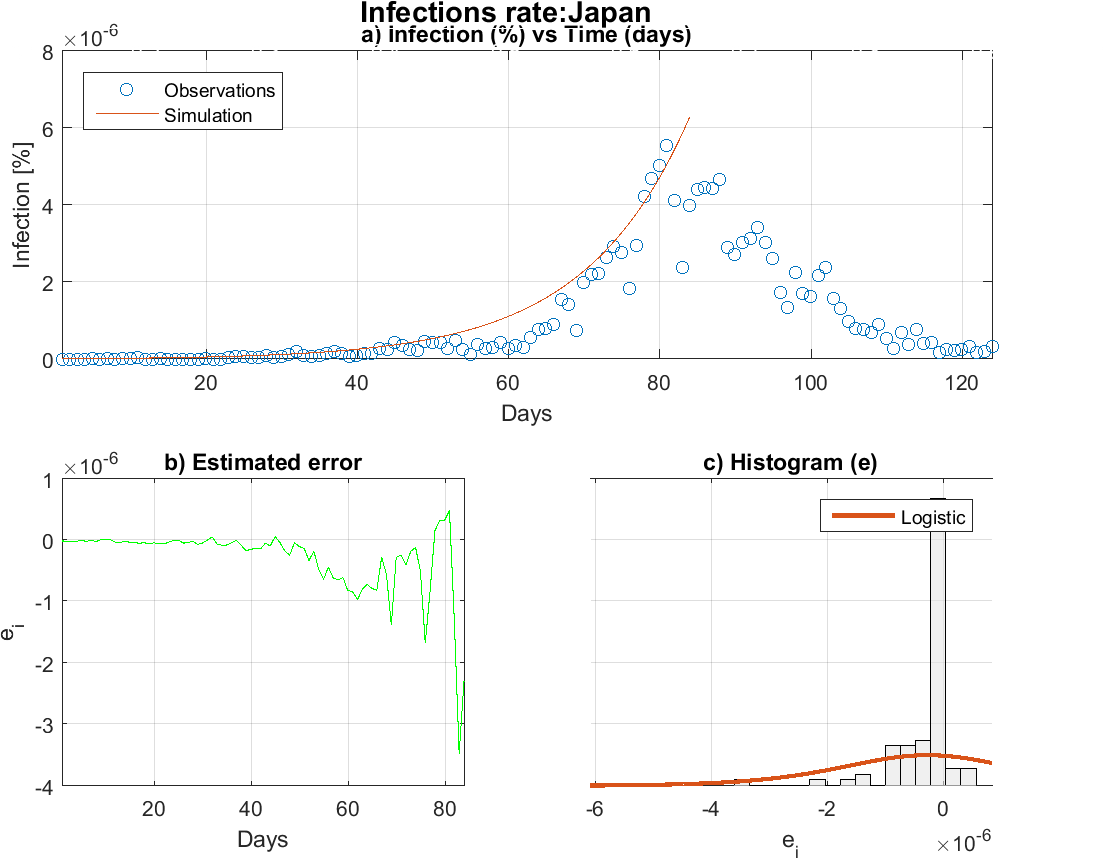


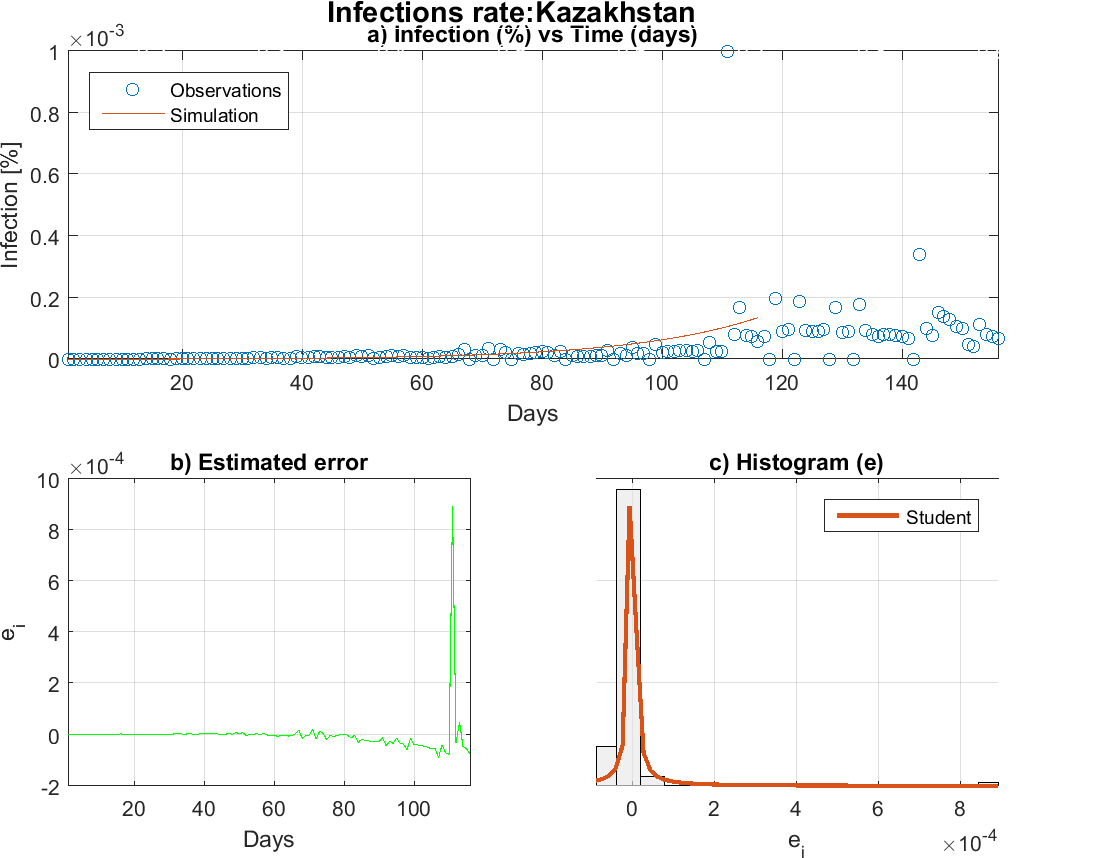


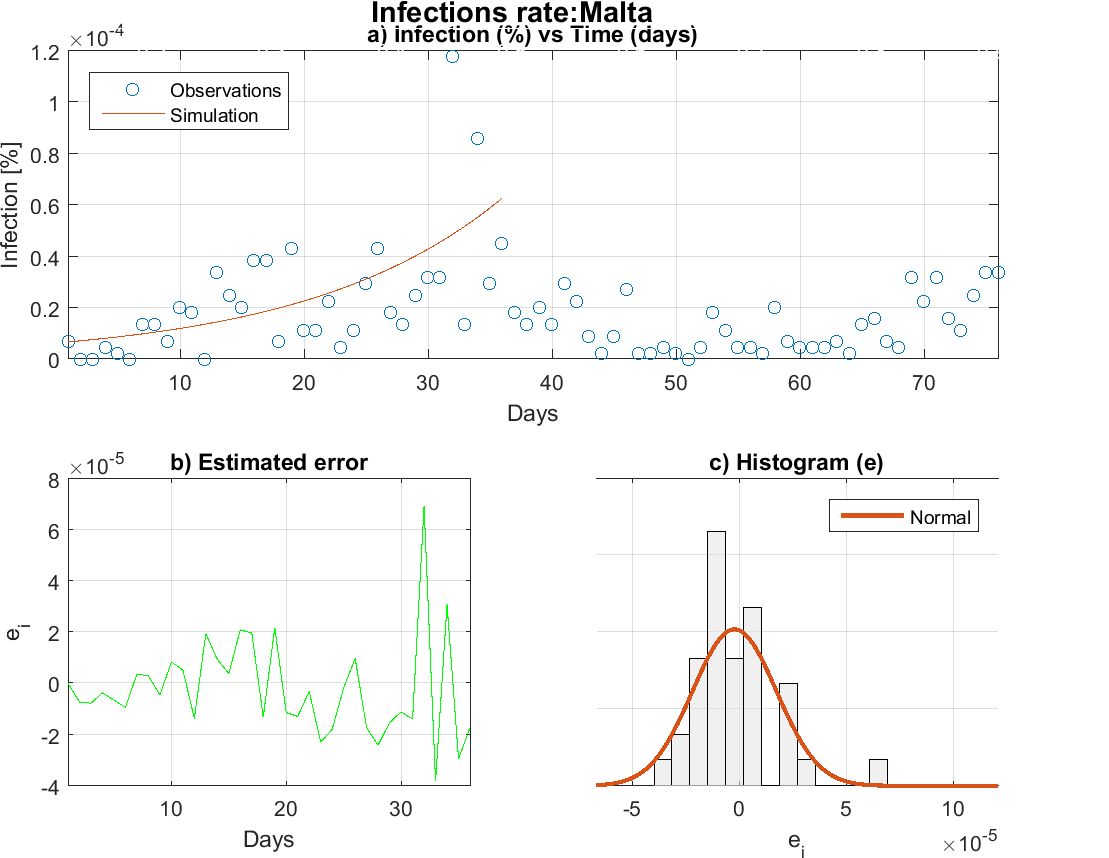


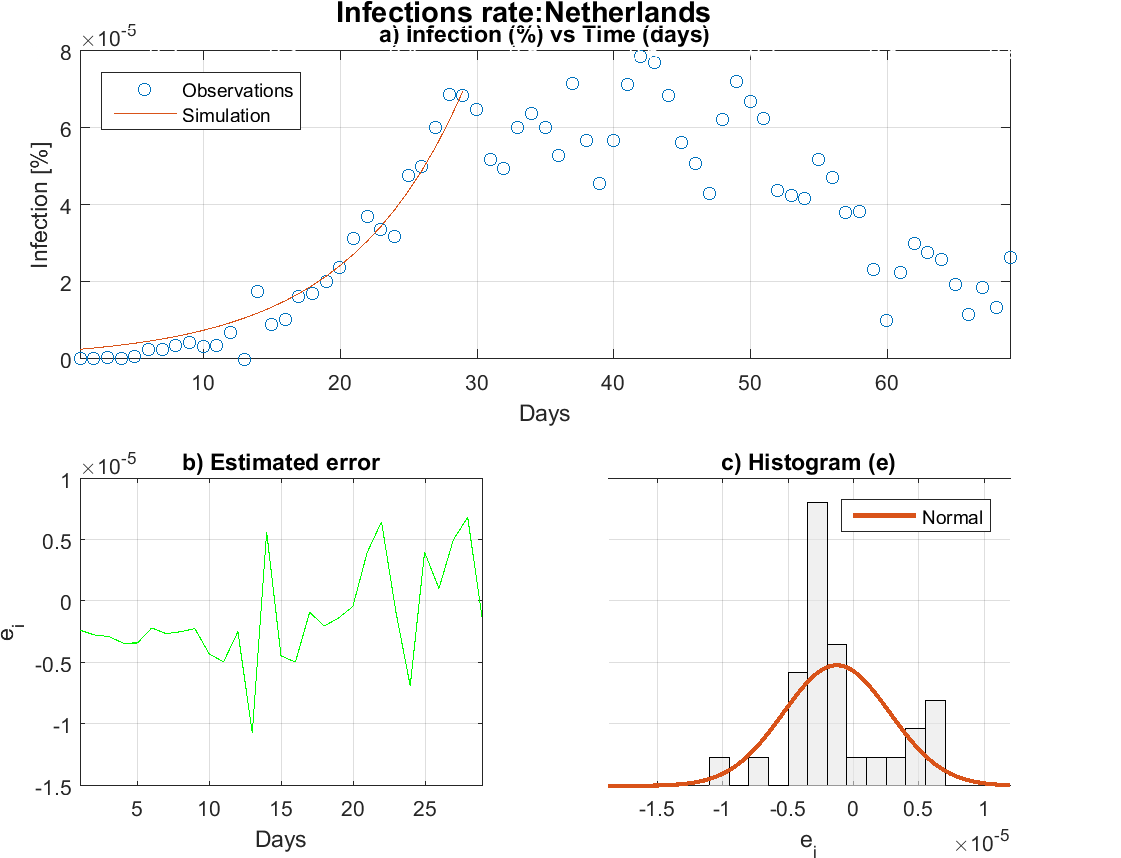


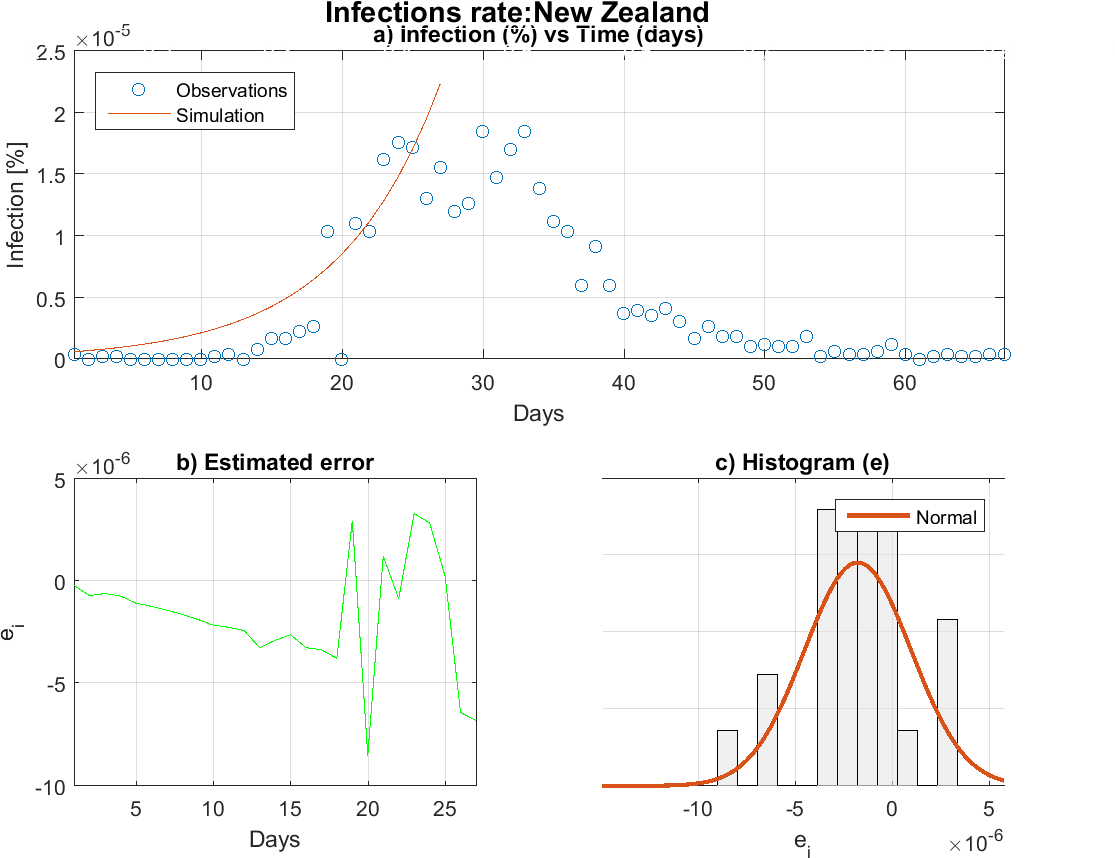


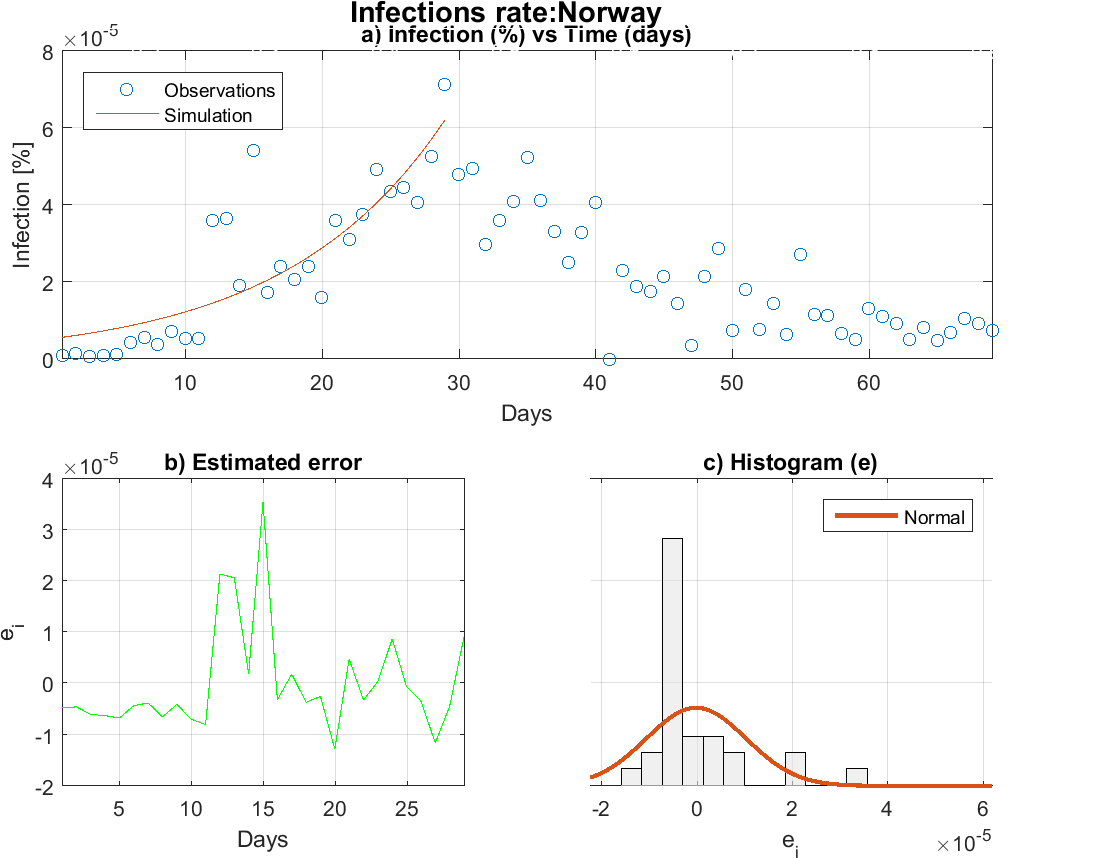


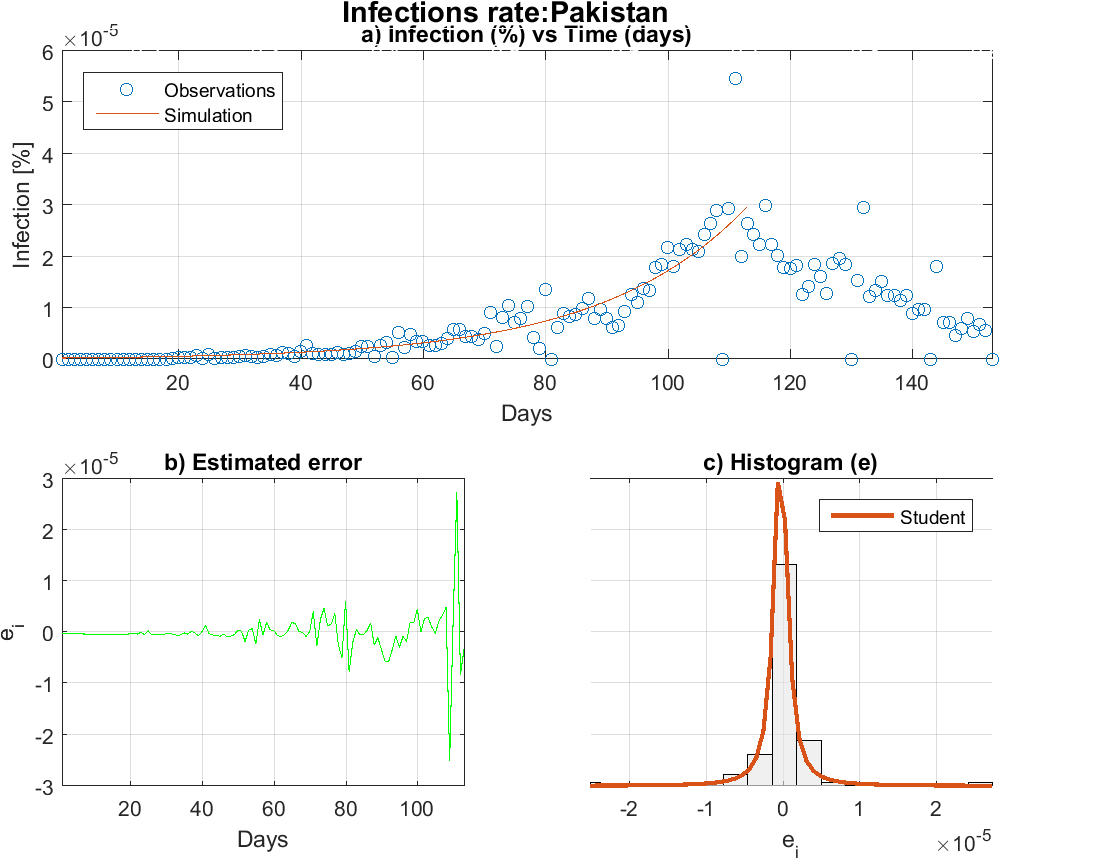


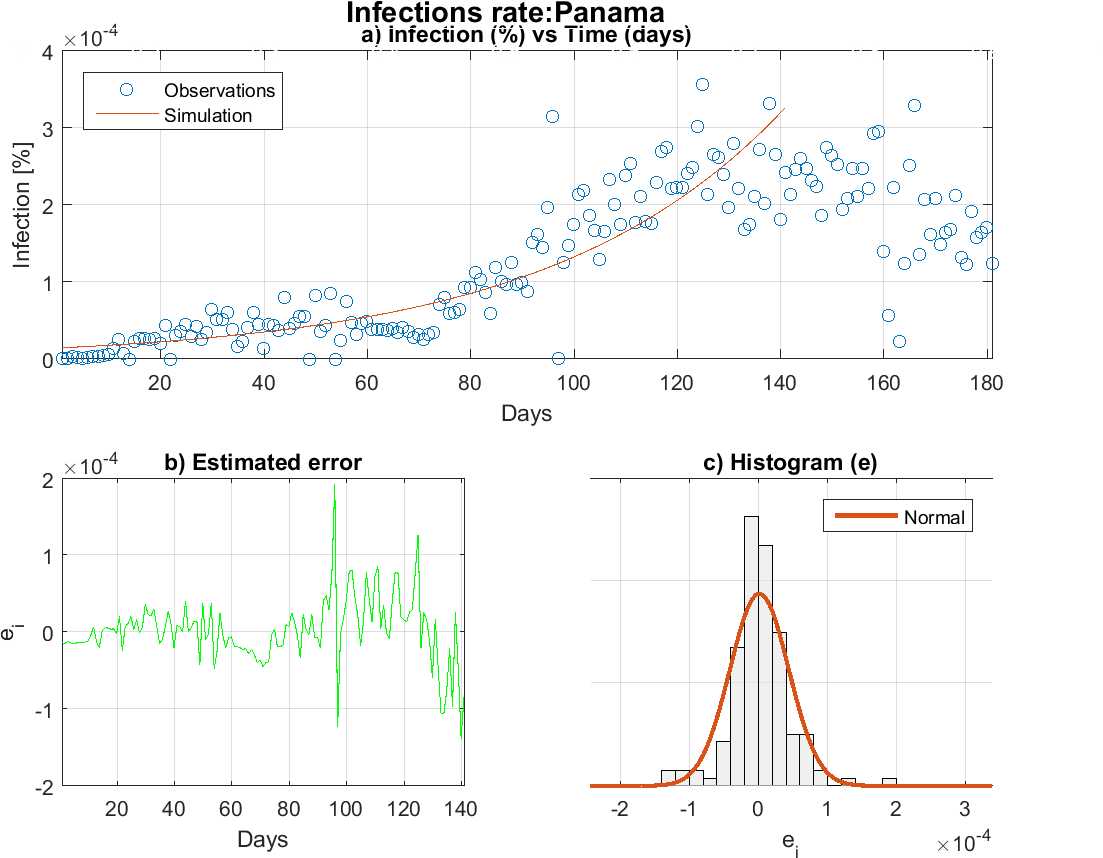


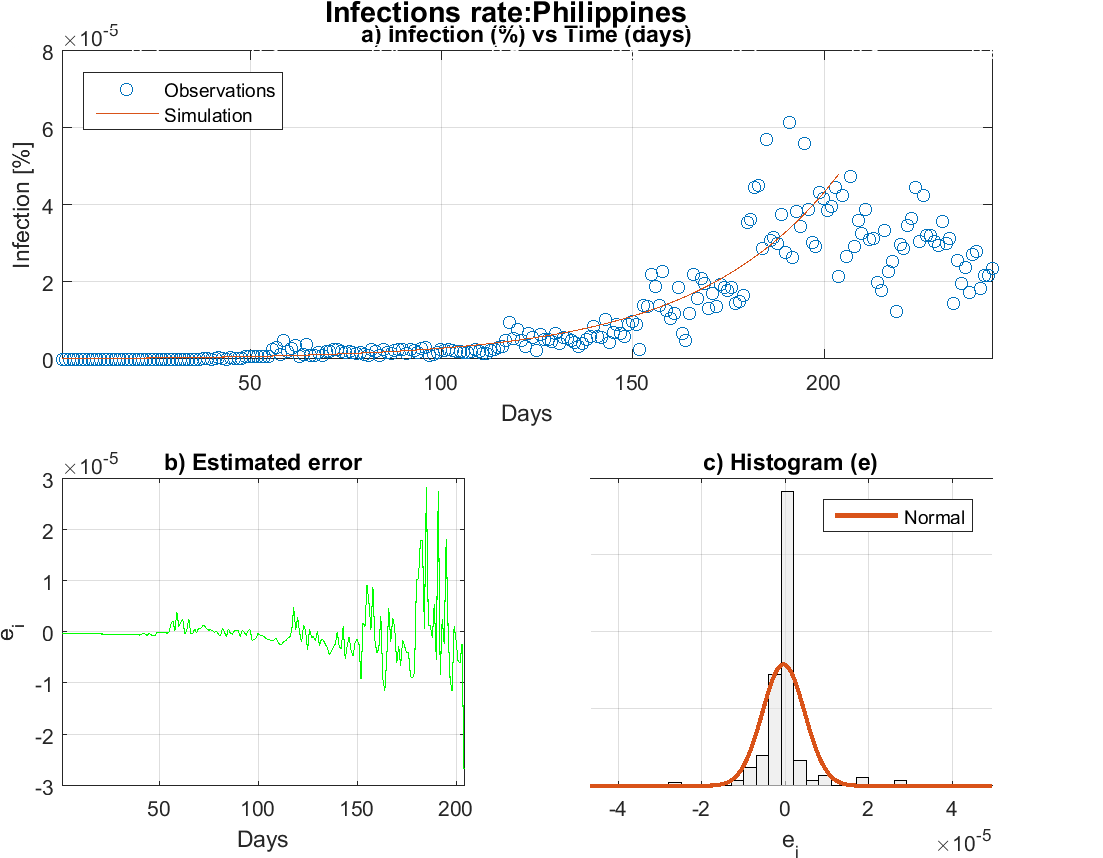


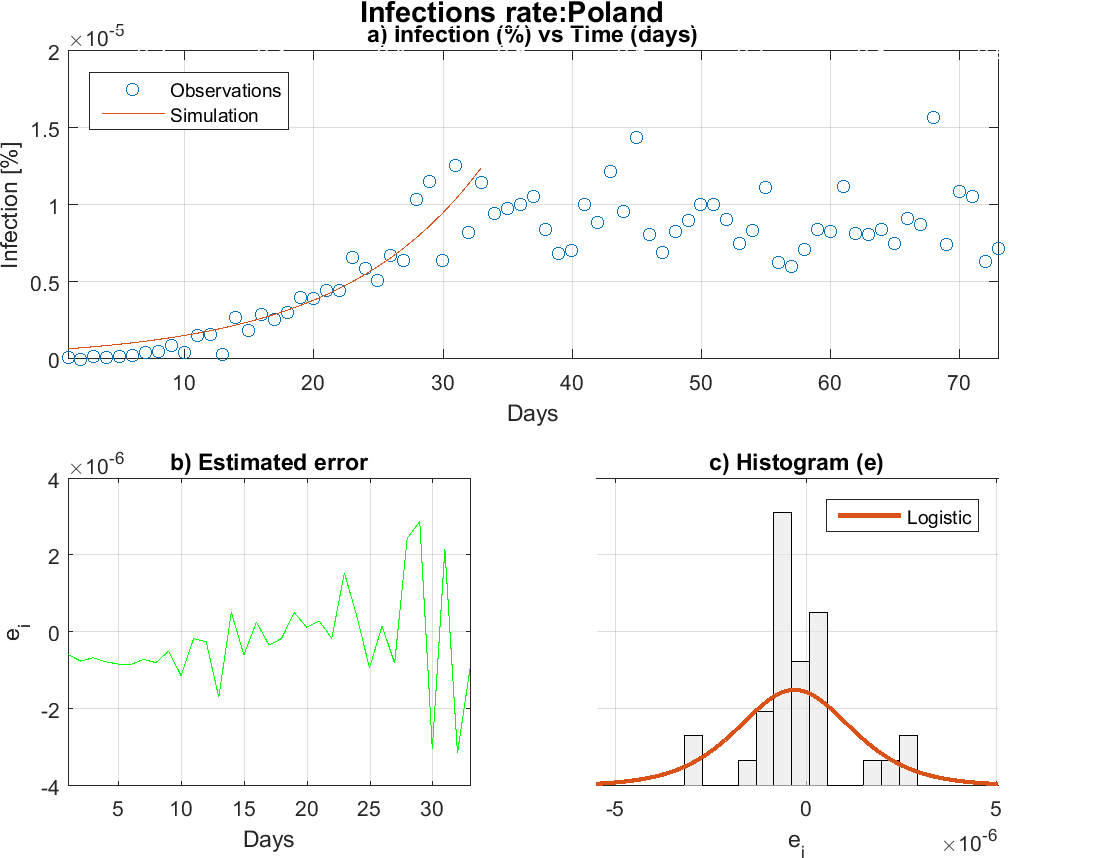


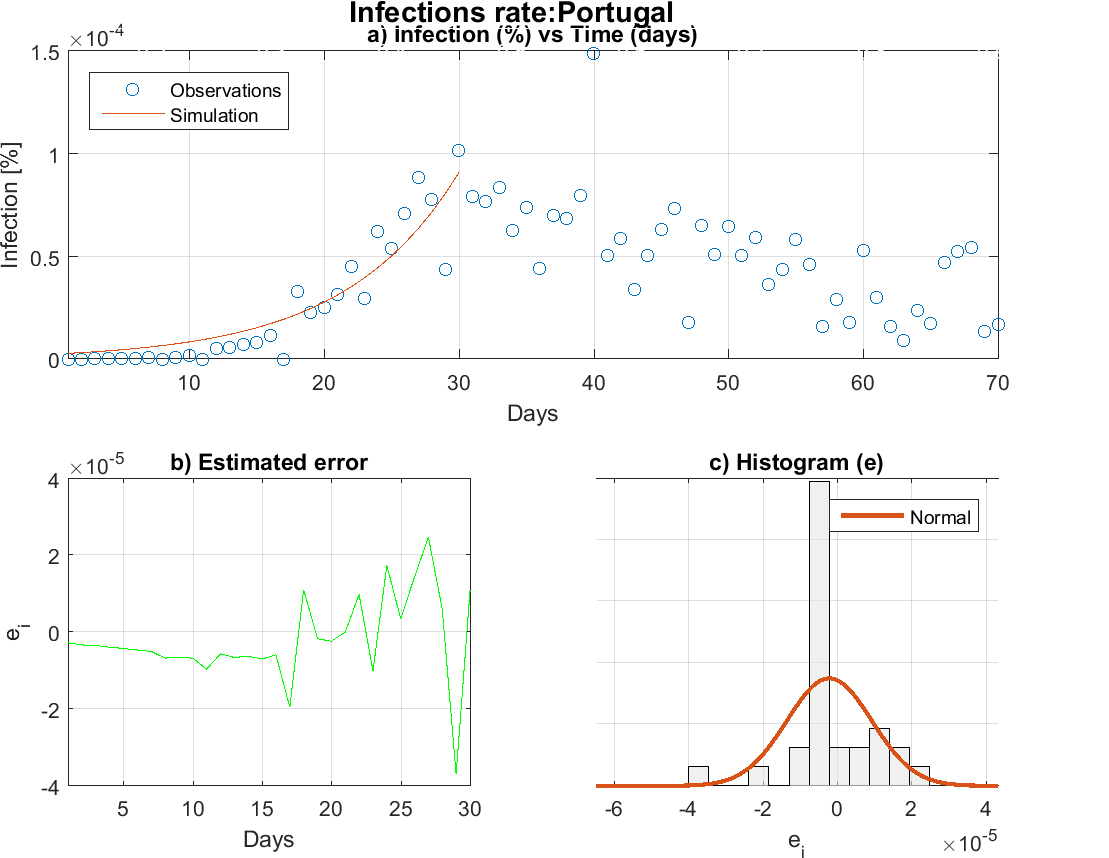


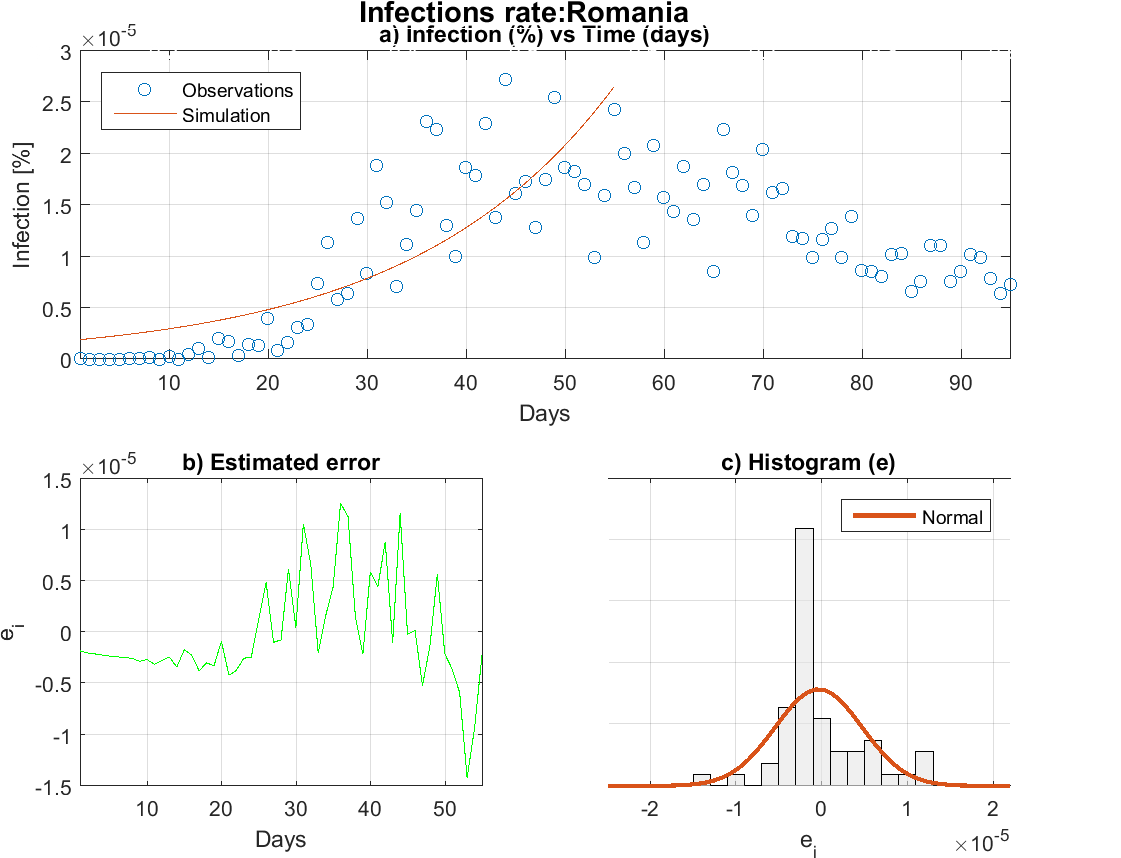


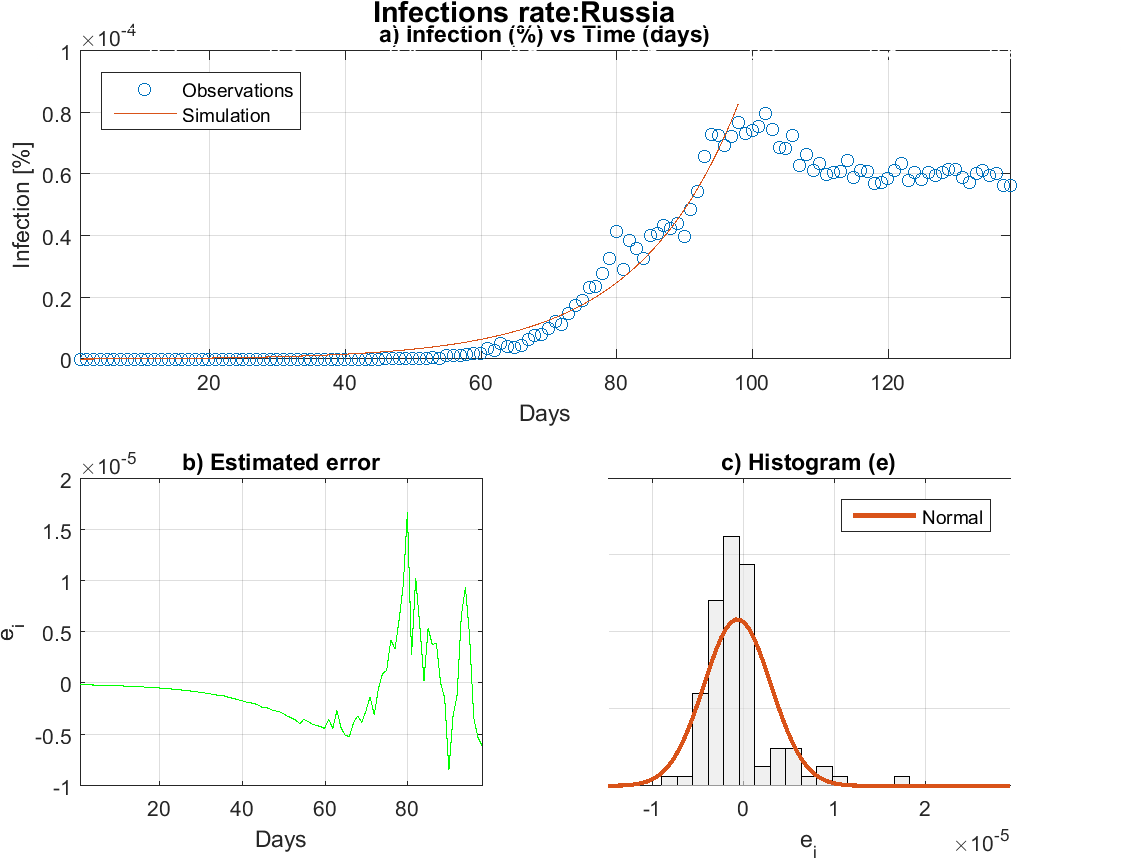


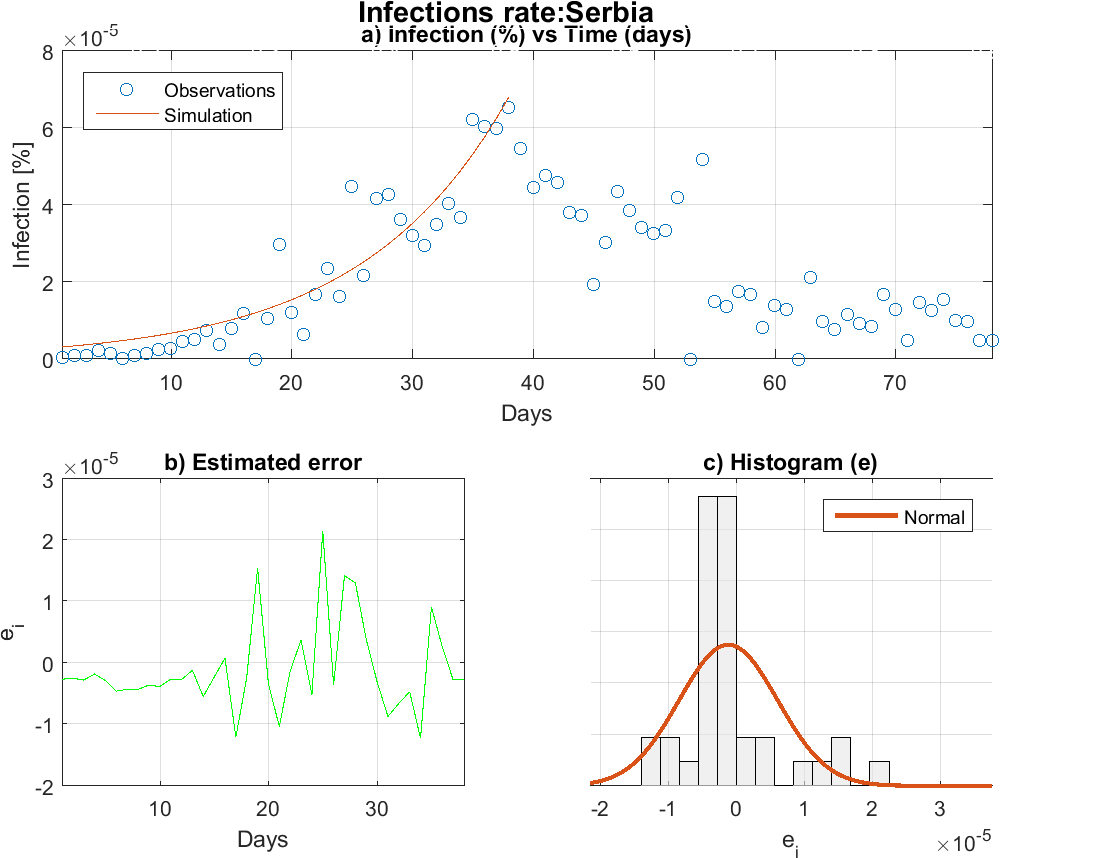


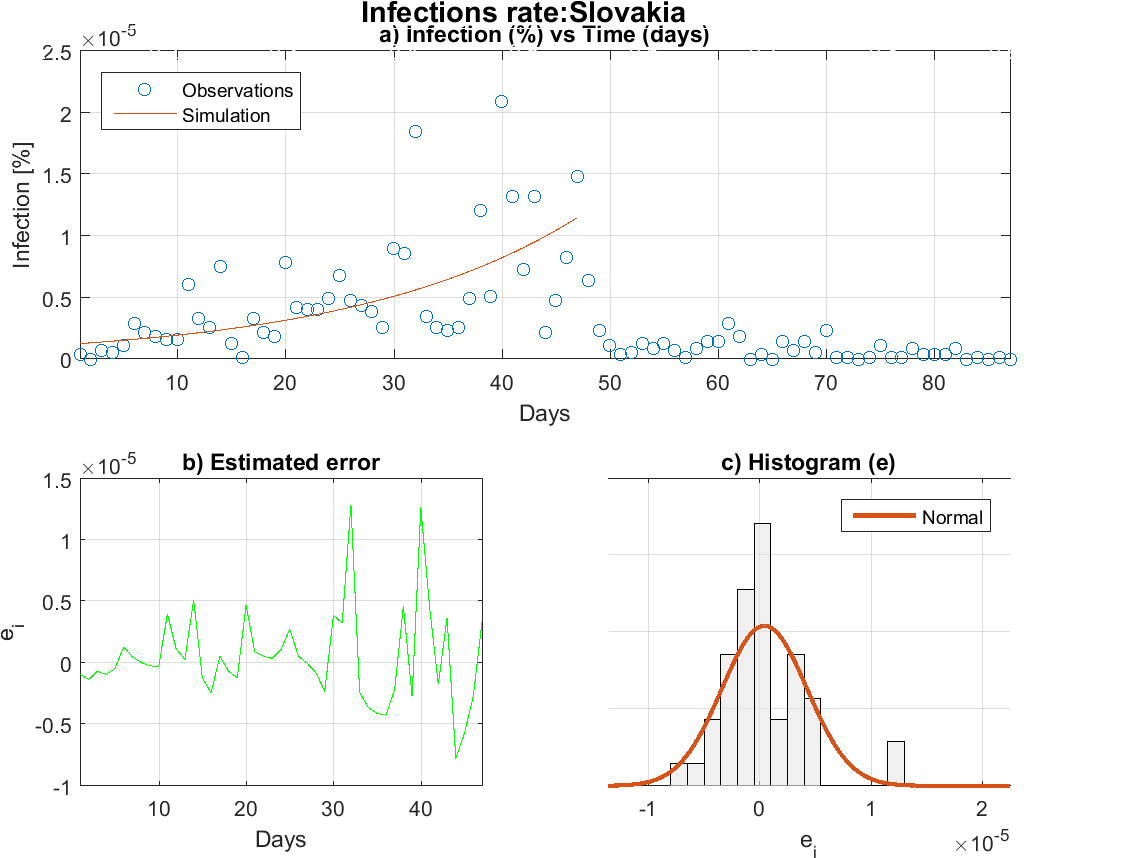


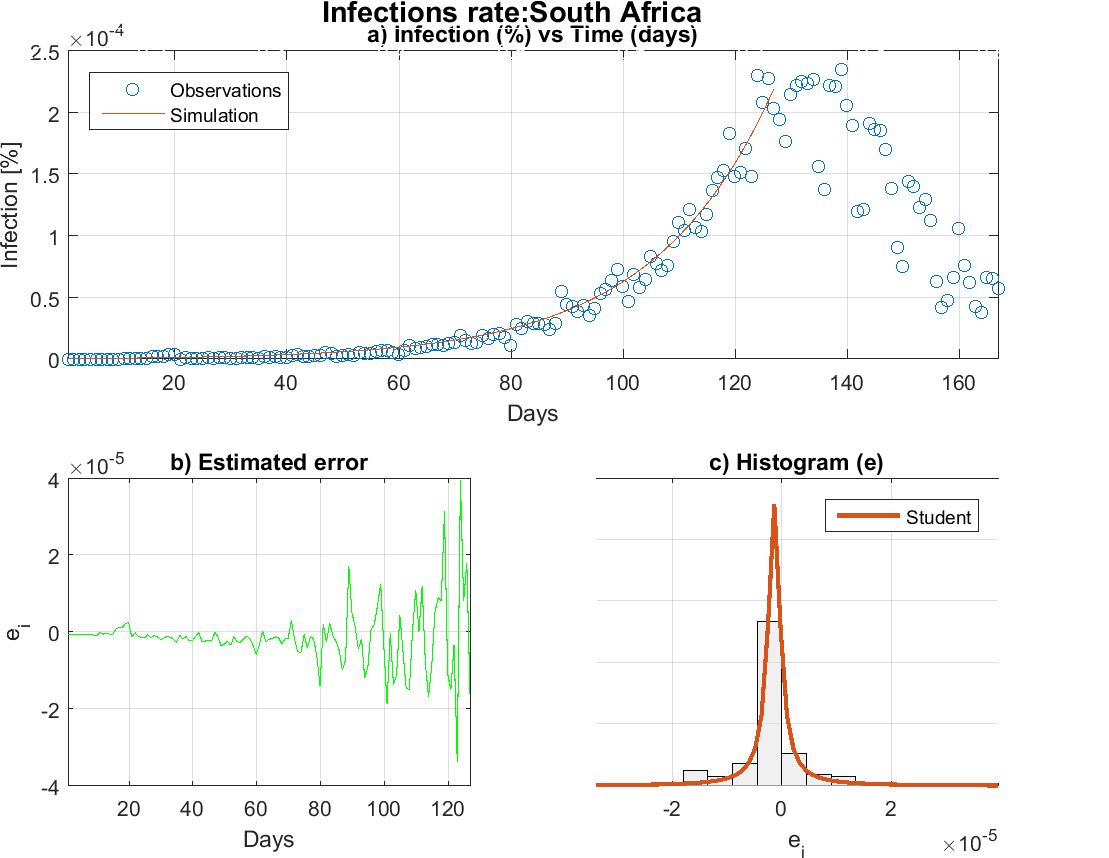


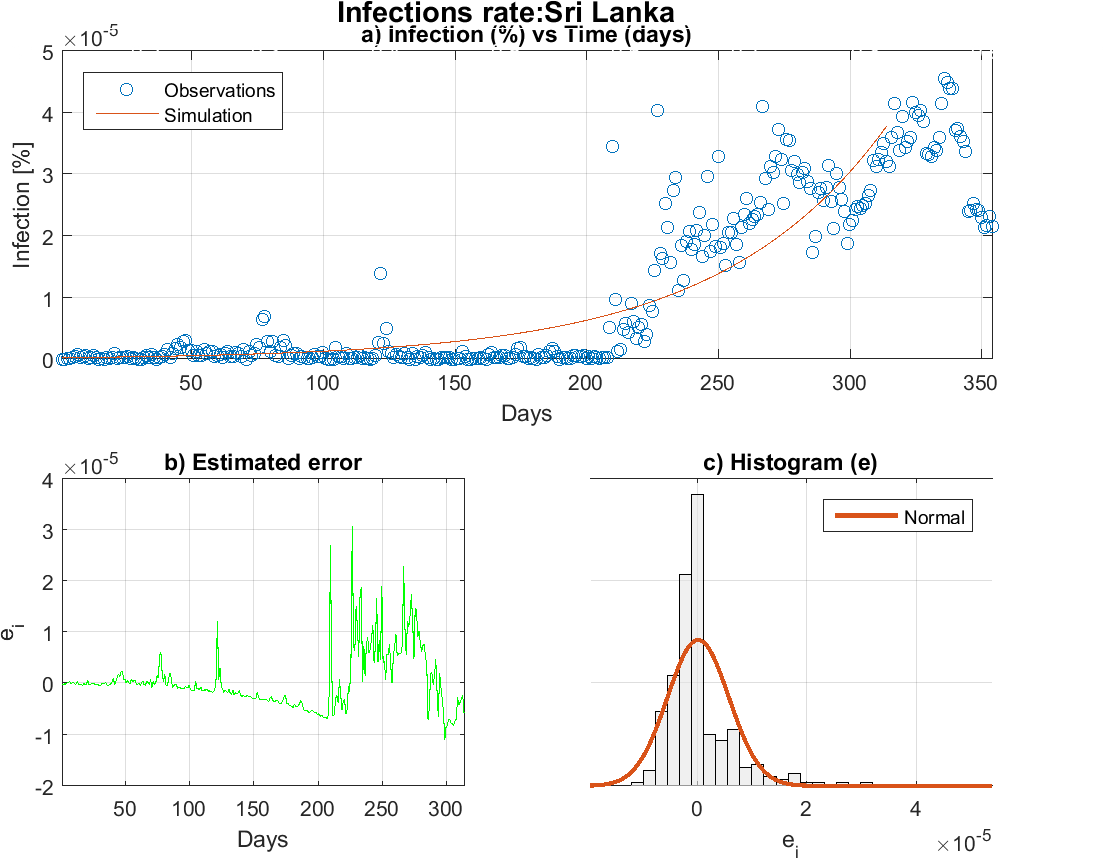


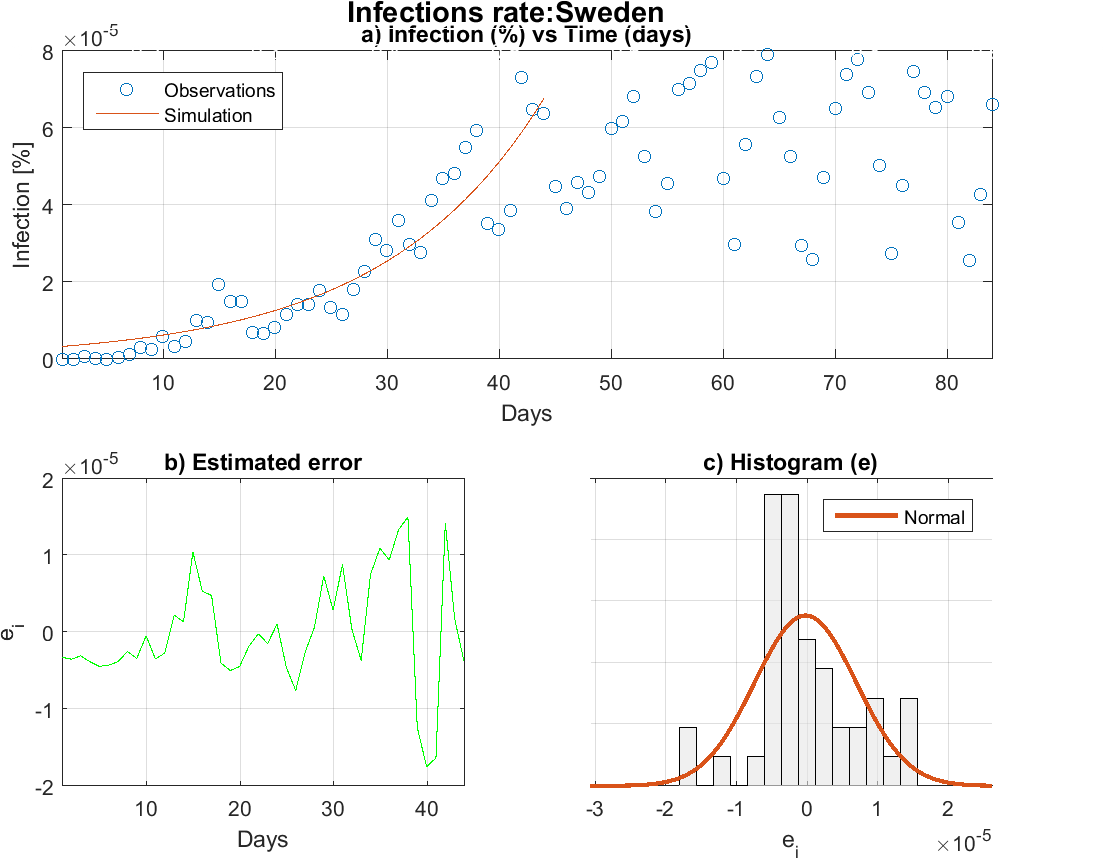


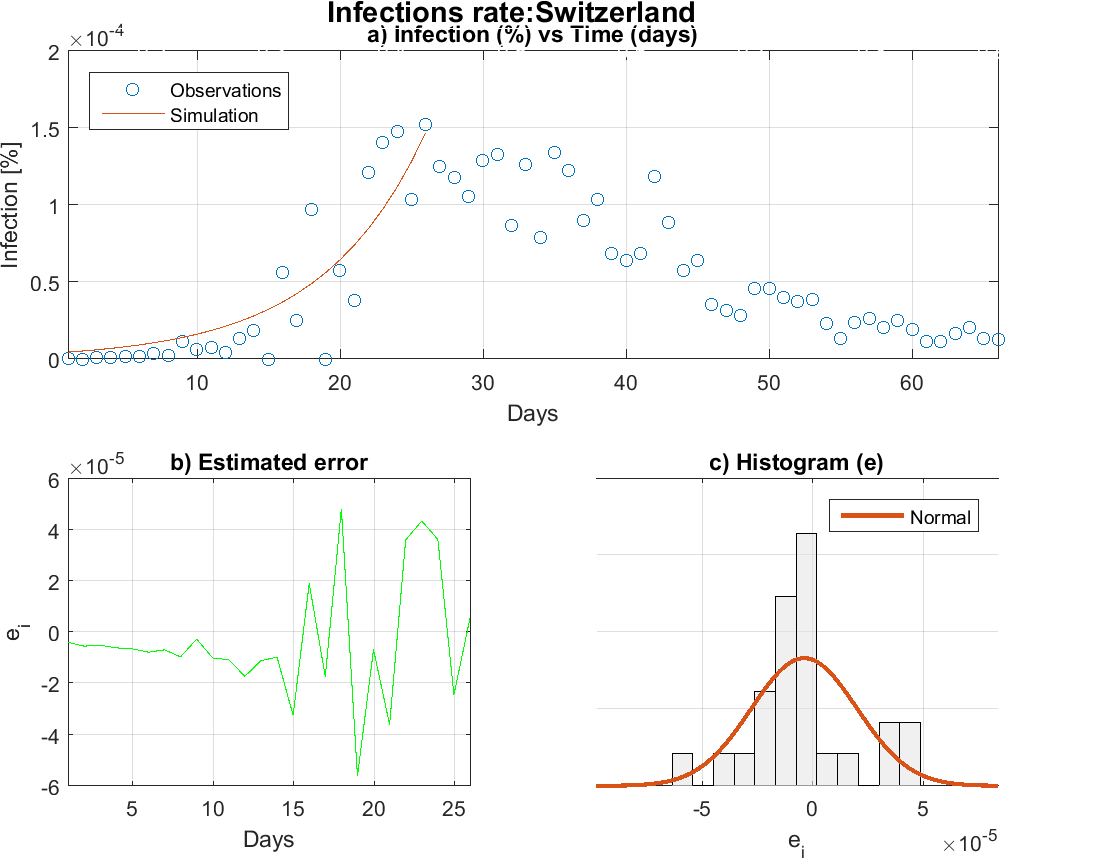


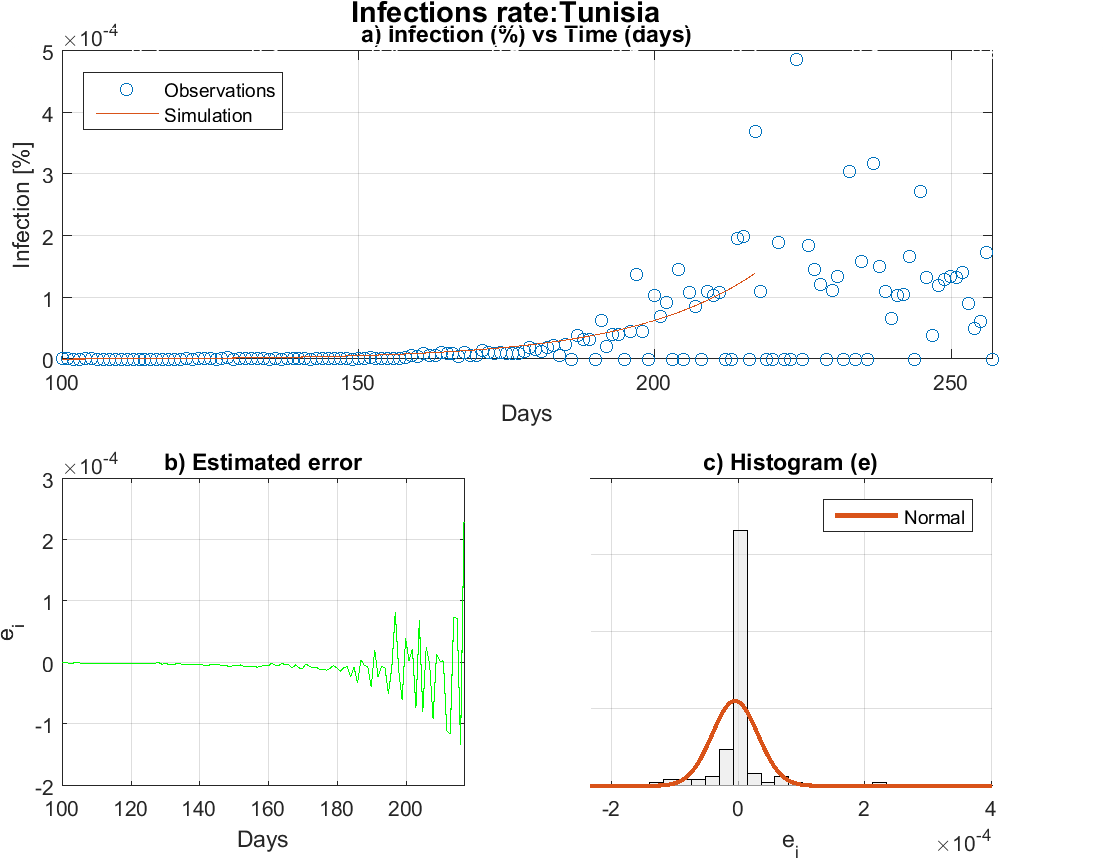


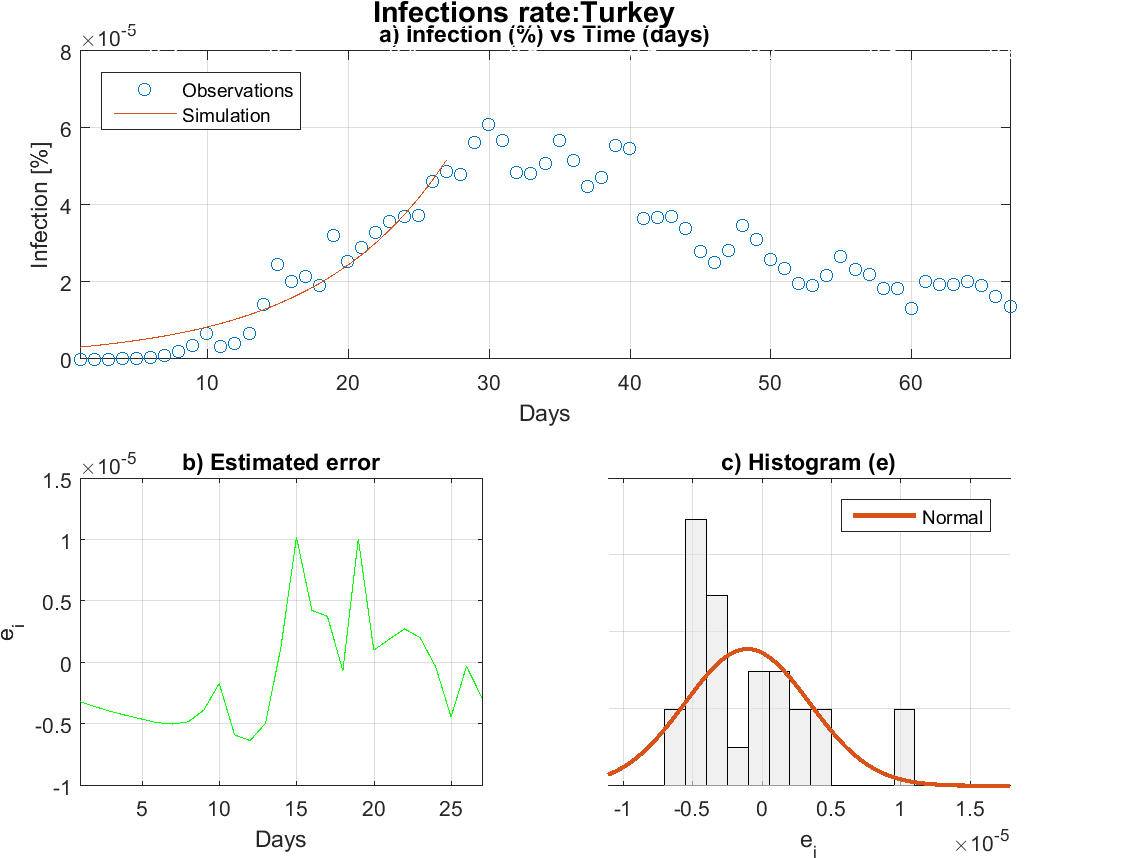


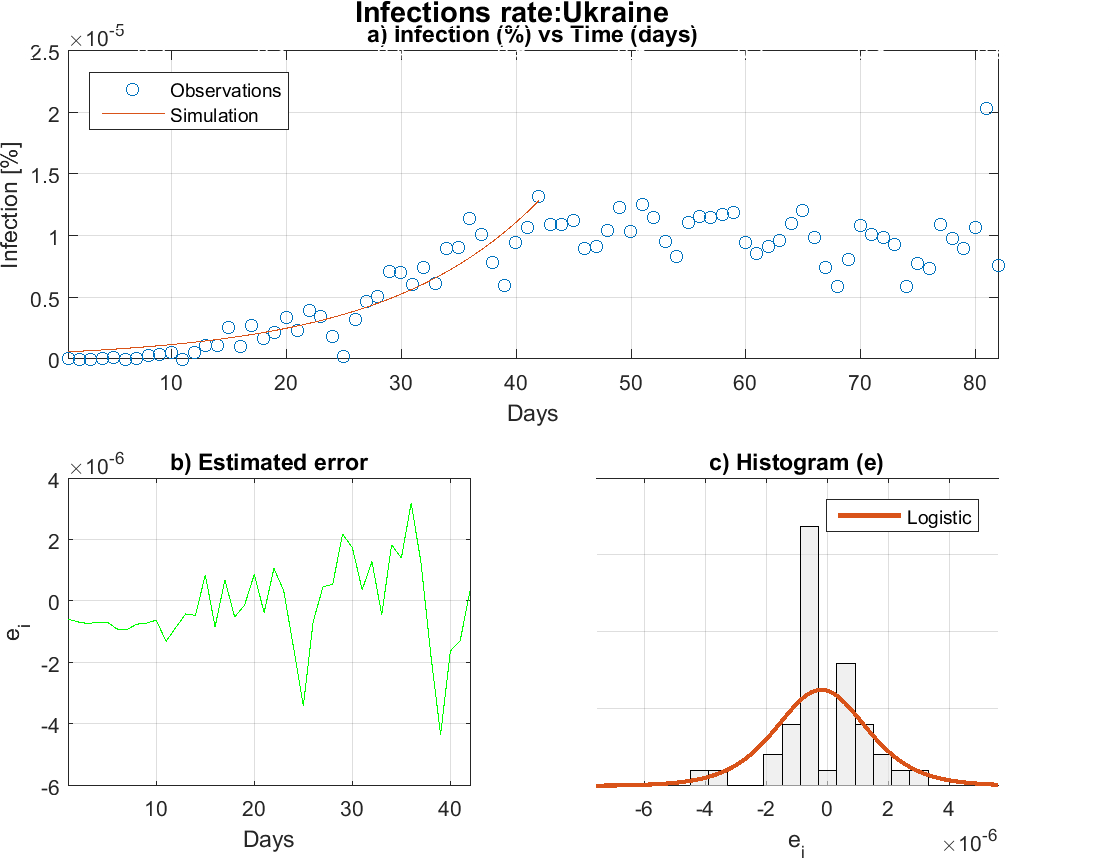


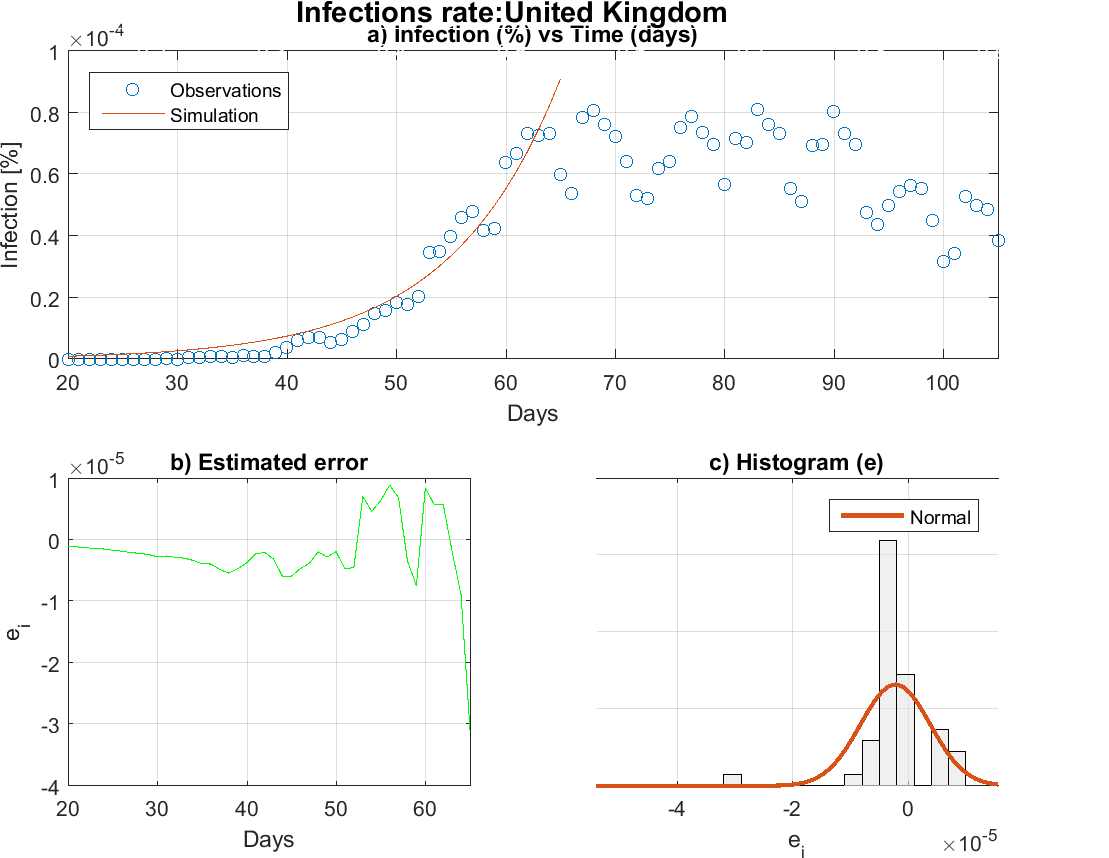

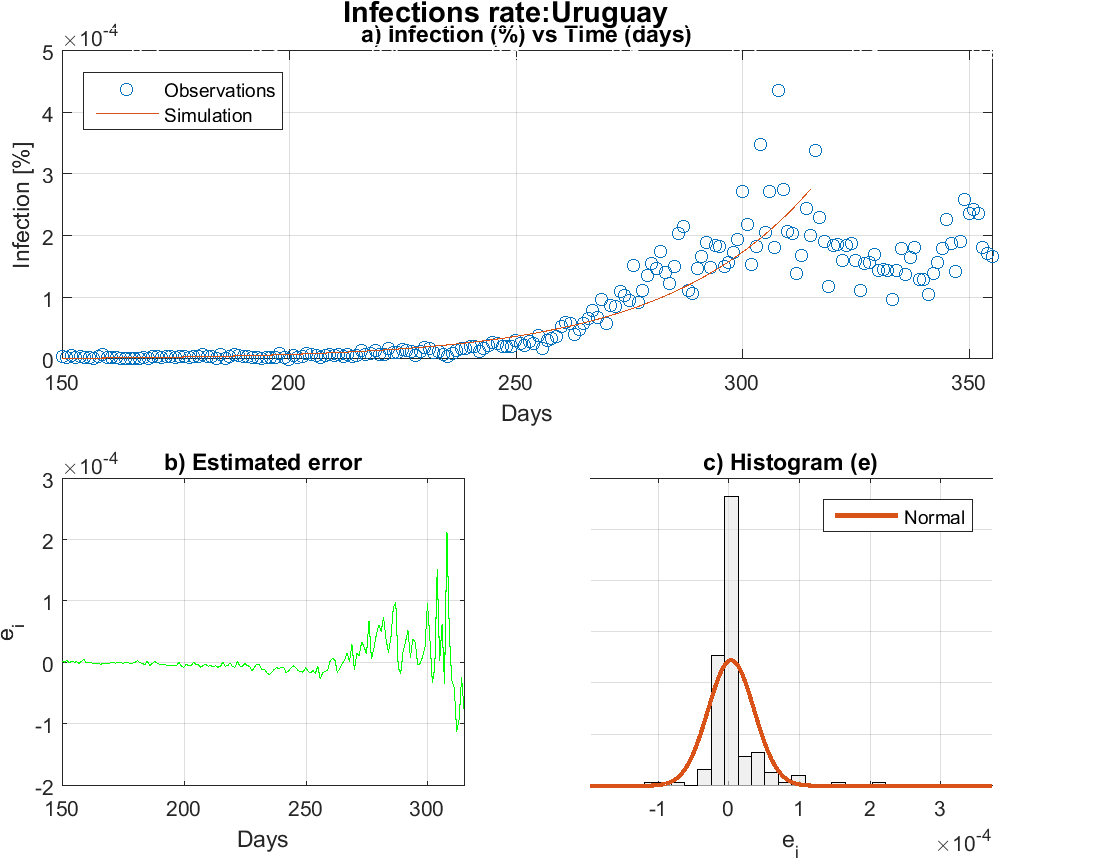


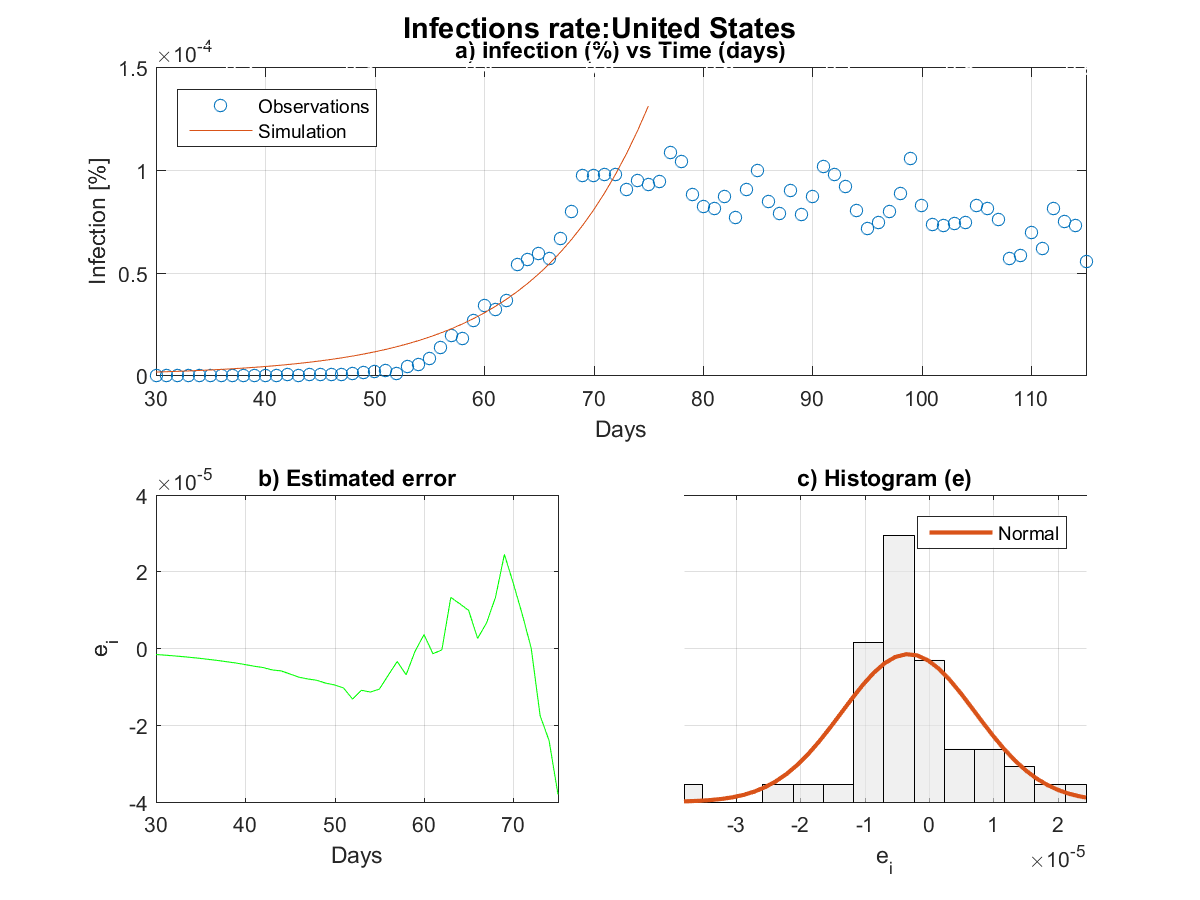

Supplement: Supplementary file 2 — Additional file 2. Plot of the observed and the simulated infection rates. [file 12889_2022_13788_MOESM2_ESM.docx]
